# Exploring Photoswitchable Properties of Two Nitro Nickel(II) Complexes with (*N*,*N*,*O*)-Donor Ligands and Their Copper(II) Analogues

**DOI:** 10.1021/acs.inorgchem.2c00526

**Published:** 2022-04-16

**Authors:** Patryk Borowski, Sylwia E. Kutniewska, Radosław Kamiński, Adam Krówczyński, Dominik Schaniel, Katarzyna N. Jarzembska

**Affiliations:** † Department of Chemistry, University of Warsaw, Żwirki i Wigury 101, 02-089 Warsaw, Poland; ‡ Université de Lorraine, CNRS, CRM^2^, 54000 Nancy, France

## Abstract

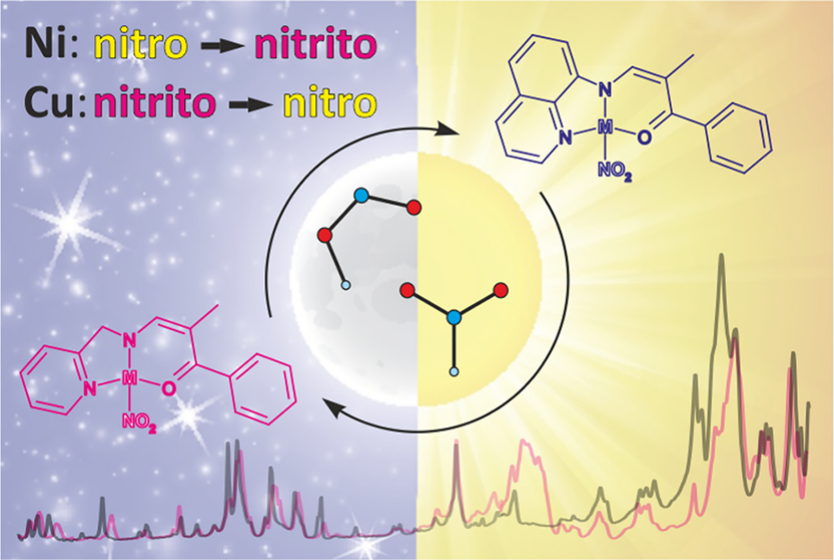

Two photoswitchable nickel(II) nitro coordination compounds and their copper(II) analogues are reported. In all these systems, the metal center is chelated by (*N*,*N*,*O*)-donor ligands containing either 2-picolylamine or 8-aminoquinoline fragments. The studied compounds were thoroughly investigated using crystallographic and spectroscopic techniques supplemented by computational analysis. They are easy to synthesize and stable, and all compounds undergo the nitro group isomerization reaction. Nevertheless, there are significant differences between the copper and nickel systems regarding their structural and switchable properties. According to the solid-state IR spectroscopy results, 400–660 nm light irradiation of the ground-state (η^2^-*O*,*O*′)-κ-nitrito copper(II) complexes at 10 K induces a rather moderate conversion to a metastable linkage isomer, which is visible only up to approximately 60–80 K. In turn, upon visible light irradiation (ca. 530 nm excitation wavelength), the ground-state nitro isomers of the examined nickel(II) complexes transform into the *endo*-nitrito forms. It was possible to achieve about 35% conversion for both nickel(II) systems and to determine the resulting crystal structures at 160 K in the case of single crystals after 30–45 min of exposure to LED light (crystals decayed with longer irradiation), and roughly 95% conversion was achieved for thin-film samples as indicated by the IR spectroscopy results. Traces of the *endo*-nitrito linkage isomers remained up to 200–220 K, and the isomerization reaction was proven to be fully reversible.

## Abstract 2

A pair of photoswitchable nickel(II) nitro complexes and their copper(II) analogues in which the metal center is chelated by (*N*,*N*,*O*)-donor ligands containing either 2-picolylamine or 8-aminoquinoline fragments are reported. The compounds are easy to synthesize and stable. Furthermore, as indicated by thorough crystallographic, spectroscopic, and computational analyses, the examined nickel systems appear to exhibit the desired reversibility and high conversion of the nitro group isomerization reaction in the solid state.

## 1 Introduction

Molecular switches triggered by UV–vis light may find wide applications in high-capacity storage devices,^1^ optoelectronics,^2^ medicine,^3^ as color-changing materials, etc.^4,5^ Therefore, the importance of studies dedicated to the conscious design of new functional photoswitchable systems with the desired properties cannot be overestimated.^6−11^ Transition-metal complexes in which the metal center is coordinated by molecular fragments that can exist in multiple isomeric forms are among the potential functional materials of this kind.^12^ Examples of ambidentate ligands known to display solid-state linkage isomerism include NO,^13,14^ NO_2_,^8,12,15−25^ SO_2_,^26−29^ SCN,^30^ and N_2._^31^ Transition-metal complexes containing nickel,^12,22,24,25^ cobalt,^32−34^ iron,^35^ ruthenium,^13,36^ osmium,^37^ palladium, or platinum^19−21,23^ centers are the most representative examples of such systems. Light-induced structural changes in crystals can be investigated using photocrystallographic methods.^9,38−40^ As far as copper complexes are concerned, to date, no multi-temperature X-ray diffraction or photocrystallographic studies of the ONO isomerization reaction have been reported; however, mixtures of various linkage isomers were observed in the solid state.^41,42^

**Scheme 1:**
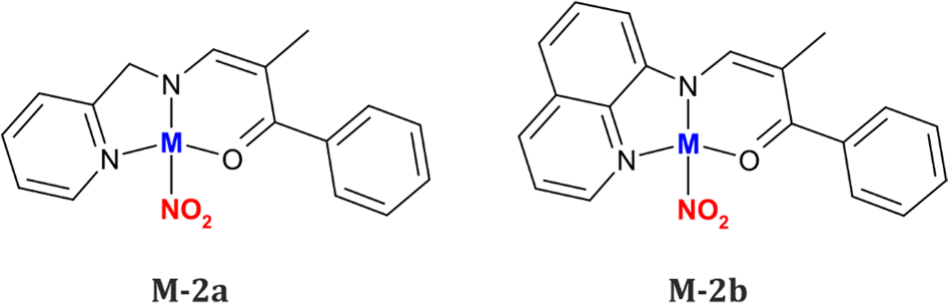
Schematic Representation of the Studied Nickel(II) and Copper(II) Complexes (M = Cu or Ni)

In view of the above, two new nitro Ni^II^ complexes and their Cu^II^ analogues, namely **2a** ((*N*,*N*,*O*)-(2-methyl-1-phenyl-3-(2'-pikoliloamido)prop-2-en-1-one) nickel(II)/copper(II) nitrite complex) and **2b** ((*N*,*N*,*O*)-(2-methyl-1-phenyl-3-(8'-(quinolin)ami-do)methylene)-prop-2-en-1-one) nickel(II)/copper(II) nitrite complex), were designed and synthesized (Scheme 1). The metal centers in these compounds are chelated by (*N*,*N*,*O*)-donor ligands and the ambidentate nitro moiety.

Their structural and switchable properties in the solid state, including the effects of the metal centers and modifications of the aromatic ligand fragments, were thoroughly investigated and analyzed. It is worth noting that the first solid-state experiments confirming the photoisomerization of the nitro group in copper systems are reported in this contribution. The current study constitutes a part of our wider project dedicated to photoswitchable fourth-row transition-metal nitro complexes.^15,16^

## 2 Experimental Section

### 2.1 Synthesis

All solvents and substrates were purchased from chemical companies and used without further purification. The synthesis is analogous to that described in our previous contribution^15^ (the reaction scheme is shown in Scheme 1S). To a mixture of 1.0 mmol propiophenone in 2.0 mmol ethyl formate was added 1.0 mmol sodium in 20 mL of Et_2_O, and the reaction mixture was stirred for 12 h. After solvent evaporation, the obtained hydroxymethyleneketone sodium salt was dissolved in 30 mL of AcOH. To the solution was then added 1.0 mmol 2-picolylamine or 8-aminoquinoline in 10 mL of AcOH. The obtained intermediate product **1a** or **1b**(43) (Supporting Information) was not isolated; after the solution was brought to a boil, 1.2 mmol Ni(OAc)_2_ or Cu(OAc)_2_ in 10 mL of MeOH, respectively, was added. Then, the solution was stirred for 2 h without heating. The dark brown mixture was purified by filtration. To the mixture was then added ca. 2.0 mmol LiNO_2_ in MeOH. The resulting mixture containing the final product (**M-2a** or **M-2b**, M = Ni or Cu) was cooled using an ice bath, and the product was filtered. Small brownish crystals, in the case of nickel(II) compounds, and green crystals, in the case of the copper(II) analogues, suitable for single-crystal X-ray diffraction experiments were grown via the vapor diffusion crystallization method using *n*-hexane and MeOH as solvents.

Nuclear magnetic resonance (NMR) spectra (collected only for the nickel complexes) were recorded with an Agilent NMR 400 MHz Varian spectrometer; ^1^H chemical shifts are given relative to TMS using residual solvent resonances. The ^1^H NMR spectra in CDCl_3_ show broadened signals, which may attributed to a reversible NO_2_ group dissociation in the presence of small amounts of water. Elemental analyses were carried out with an Elementar Vario EL III analyzer.

#### 2.1.1 **Ni-2a**

Yield: 0.228 g (64%). ^1^H NMR (CDCl_3_, 400 MHz): δ 7.75 (m, 2H), 7.31 (m, 6H), 7.15 (m, 1H), 7.01 (m, 1H), 4.79 (bs, 2H, NCH_2_), 1.93 (bs, 3H, CH_3_) ppm. Elemental analysis: C_16_H_15_N_3_Ni_1_O_3_ (356.00). Calculated: C 53.98%, H 4.25%, N 11.80%. Found: C 54.22%, H 4.40%, N 11.70%.

#### 2.1.2 **Ni-2b**

Yield: 0.231 g (59%). ^1^H NMR (CDCl_3_, 400 MHz): δ 8.29 (m, 1H), 8.02 (m, 1H), 7.65 (bd, *J* = 8.0 Hz, 2H), 7.55 (t, *J* = 8.0 Hz, 1H), 7.49–7.29 (m, 8H), 2.08 (bs, 3H, CH_3_). Elemental analysis: C_19_H_15_N_3_Ni_1_O_3_ (392.04). Calculated: C 58.21%, H 3.86%, N 10.72%. Found: C 58.30%, H 3.99%, N 10.59%.

#### 2.1.3 **Cu-2a**

Yield: 0.209 g (58%). Elemental analysis: C_16_H_15_N_3_Cu_1_O_3_ (360.86). Calculated: C 53.26%, H 4.19%, N 11.64%. Found: C 53.11%, H 4.37%, N 11.44%.

#### 2.1.4 **Cu-2b**

Yield: 0.206 g (52%). Elemental analysis: C_19_H_15_N_3_Cu_1_O_3_ (396.89). Calculated: C 57.50%, H 3.81%, N 10.59%. Found: C 57.91%, H 3.98%, N 10.41%.

### 2.2 X-ray Diffraction

All X-ray diffraction experiments (including the preliminary ones) were carried out on a Rigaku Oxford Diffraction SuperNova single-crystal diffractometer equipped with a CCD detector, a copper microfocus X-ray source, a low-temperature nitrogen gas flow Oxford Cryosystems device, and our homemade light-delivery device,^44^ which allows in situ photocrystallographic experiments. The optimal data-collection strategy took into account the mounted light-delivery device and was prepared using the native diffractometer software. For photocrystallographic experiments, the same strategy (in which only the exposure time was adjusted for various temperatures) was used for all data collected for a given crystal. All data collections were carried out in complete darkness (the sample mounting and centering was done at room temperature prior to any further data collection, and all experiments were performed with all the diffractometer lights permanently switched off). The overall procedure used during photocrystallographic experiments for the **Ni-2a** and **Ni-2b** crystals was as follows: (i) The crystal was mounted at room temperature and cooled to 140 or 160 K at 360 K·s^–1^. (ii) Light irradiation (45 and 30 min for **Ni-2a** and **Ni-2b**, respectively) was performed using the previously selected LED with central wavelengths of 590 and 530 nm, respectively (Thorlabs fiber-coupled LEDs M590F2 and M530F2, respectively; light was delivered through the 400 μm core multimode solarization-resistant fiber optics); during light irradiation, the crystal was continuously rotated to ensure the most uniform exposure. (iii) X-ray diffraction experiments were performed at various temperatures; the **Ni-2a** data sets were collected at temperatures ranging from 140 to 200 K with steps of 20 K, and the **Ni-2b** data sets were collected only at 160 and 240 K due to crystal degradation upon temperature changes. No color changes were observed for crystals upon exposure to LED light. For exact photocrystallographic data collection codes and the measurement sequence for each sample, see the Supporting Information (Table 1S). Further data processing (i.e., unit-cell determination, raw diffraction-frame integration, absorption correction, and scaling) was the same for all data collected. All structures were solved using an intrinsic phasing method as implemented in the *SHELXT* program^45^ and refined with the *JANA* package^46^ within the independent atom model (IAM) approximation.^47,48^ The disordered structures were modeled using a standard splitting model in which the initial positions of the metastable linkage isomer atoms were determined from the residual or photodifference maps.^47,48^ All the final refinement statistics are summarized in Table 2S. The CIF files can be retrieved from the Cambridge Structural Database (CSD) (deposition numbers CCDC 2110808–2110835).^49,50^

All powder X-ray diffraction (PXRD) measurements were carried out on a Bruker AXS D8 Discover powder diffractometer equipped with a VÅNTEC detector and a copper X-ray tube. All data were collected in a parallel-beam geometry (locked-couple experiment mode) in the 2θ range from 3 to 40° with scan speed set to 1°·min^–1^. Le Bail refinements^51^ of the unit-cell and powder-profile parameters were accomplished with the *JANA* program.^52^

### 2.3 Spectroscopy

All infrared (IR) measurements were performed using a Nicolet 5700 FT-IR spectrometer (spectral resolution of 2 cm^–1^ in the range of 360–4000 cm^–1^) equipped with a closed-cycle cryostat (Oxford Optistat V01). The sample was ground, mixed with spectroscopic-grade KBr, pressed into pellets, and glued to the coldfinger of the cryostat using a silver-paste thermal adhesive. During measurements, the sample was kept in a vacuum inside the cryostat. Irradiation of the sample was achieved through the cryostat window using various LEDs (Thorlabs L and LP series), the central wavelengths of which covered the range from violet to red (from 385 to 735 nm).

Room-temperature UV–vis spectroscopic measurements were performed with a Shimadzu UV-2600i spectrometer (300–800 nm wavelength range in 0.5 nm intervals) in the transmission mode. The KBr pellets were prepared in the same way as those used for the solid-state IR measurements. The obtained spectra are presented in Figure 20S in the Supporting Information.

### 2.4 Computational Analysis

All computations were carried out using the *GAUSSIAN* package (*GAUSSIAN 16*).^53^ In the case of the quantum mechanics–molecular mechanics (QM/MM) method, the crystal environment was modeled by cutting out a shell with a radius of 15 Å around the central molecule from the examined experimental crystal structure,^54^ the C–H distances of which were set to the neutron-normalized values.^55,56^ Density functional theory (DFT) at the DFT(B3LYP)/6-311++G** level of theory was applied for the optimization of the central molecule,^57−62^ whereas the molecular shell was kept fixed and approximated with the Universal Force Field (UFF)^63^ using Hirshfeld atomic charges^64^ initially derived at the same level of theory, including both the functional and the basis set. Dimer interaction energies, isolated-molecule geometry optimizations, and normal-mode frequencies were also calculated at the DFT(B3LYP)/6-311++G** level of theory. For harmonic-mode computations, no imaginary frequencies were found. In the case of interaction energy calculations, the Grimme empirical dispersion correction^65,66^ modified by the Becke–Johnson damping function^67,68^ and a correction for basis set superposition error^69,70^ were applied. The automatic generation of input files was accomplished with the *CLUSTERGEN* program.^71^

### 2.5 Calorimetry

The differential scanning calorimetry (DSC) measurements were performed using a Mettler-Toledo DSC1 STAR^e^ system at a heating rate of 10 °C·min^–1^ under a dry N_2_ atmosphere at a constant flow rate (50 mL·min^–1^) over temperature ranges from +25 to −150, −150 to +25, and +25 to +500 °C. The obtained data were analyzed using the STAR^e^ software provided by Mettler Toledo. The total weight of a sample was accurately evaluated in a standard 40 μL aluminum crucible using a Mettler-Toledo XS105 DualRange balance.

## 3 Results and Discussion

### 3.1 Crystal Structures of Ni and Cu Complexes

**Figure 1:**
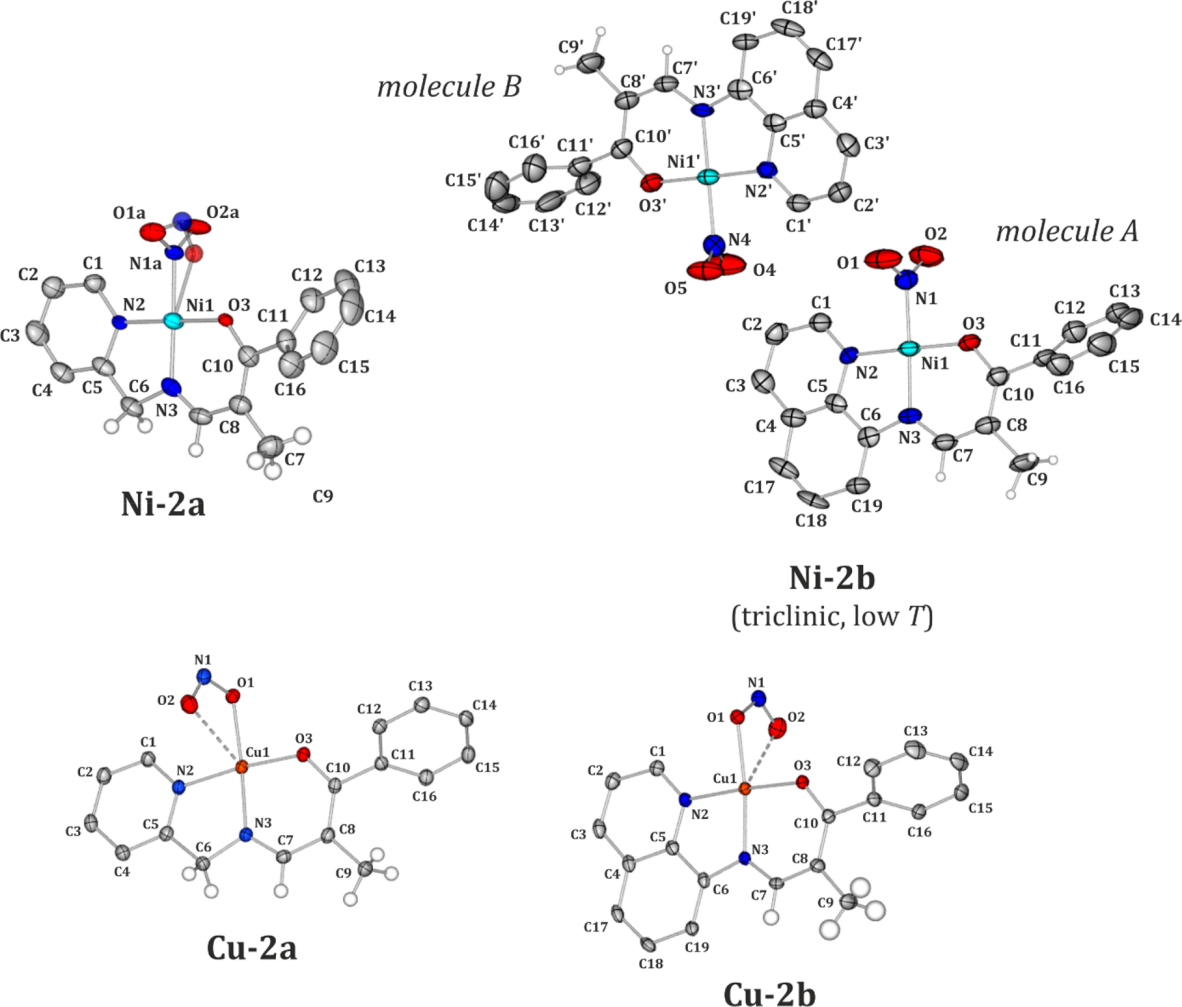
Molecular structures of all studied compounds derived from the X-ray diffraction data collected in complete darkness. Atomic thermal motion is represented as ellipsoids at the 50% probability level, selected hydrogen atoms are omitted for clarity, and the disorder is shown as a semitransparent fragment. For the **Ni-2a** complex, only the nitro isomer is labeled for clarity; for the **Ni-2b** complex, the atom labeling is shown for the low-temperature triclinic structure (the labeling of the high-temperature monoclinic structure follows that of the molecule A).

Three crystal structures of the studied coordination compounds exhibit the triclinic *P*1̅ space group symmetry, with one molecule comprising the asymmetric unit (ASU) in the 100–300 K temperature range. The **Ni-2b** crystals constitute an exception. At room temperature (RT), it appears that the crystal structure of **Ni-2b** belongs to the *P*2_1_/*c* space group with one molecule in the ASU, whereas at 160 K it is described by the *P*1̅ space group with two symmetry-independent species comprising the ASU. Further DSC analysis (Figure 1S) and multi-temperature structural studies revealed that this system indeed undergoes a monoclinic-to-triclinic phase transition at around 275 K, while the crystals degrade notably along with temperature decrease (the crystal diffraction becomes very poor below 160 K despite the cooling rate applied). Selected crystal-structure parameters and data collection and refinement details are summarized in Table 1 and in the Supporting Information (Table 2S).

**Table 1:** Selected Crystal Structure Parameters for All Studied Complexes^a^

compound	**Ni-2a**^b^	**Ni-2b**^c^ (monoclinic, high *T*)	**Ni-2b**^d^ (triclinic, low *T*)	**Cu-2a**	**Cu-2b**
moiety formula	C_16_H_15_N_3_Ni_1_O_3_	C_19_H_15_N_3_Ni_1_O_3_	C_19_H_15_N_3_Ni_1_O_3_	C_16_H_15_N_3_Cu_1_O_3_	C_19_H_15_N_3_Cu_1_O_3_
moiety formula mass, *M*_r_ (a.u.)	356.00	392.04	392.04	360.86	396.89
crystal system	triclinic	monoclinic	triclinic	triclinic	triclinic
space group	*P*1̅ (no. 2)	*P*2_1_/*c* (no. 14)	*P*1̅ (no. 2)	*P*1̅ (no. 2)	*P*1̅ (no. 2)
*Z*	2	4	4	2	2
*F*_000_	368	808	808	370	406
crystal color and shape	orange plate	brown plate	brown plate	brown plate	green plate
*T* (K)	100	290	160	100	100
*a* (Å)	7.4023(7)	14.1406(7)	14.1083(10)	8.435(2)	6.804(2)
*b* (Å)	8.1523(7)	6.8044(3)	6.7505(3)	9.513(2)	9.088(2)
*c* (Å)	13.6139(12)	17.9675(7)	17.8681(8)	10.739(2)	13.866(3)
α (°)	100.305(7)	90	88.724(3)	69.07(3)	75.87(3)
β (°)	101.078(8)	103.816(4)	104.156(5)	69.65(3)	83.43(3)
γ (°)	102.856(8)	90	94.061(4)	72.48(3)	84.76(3)
*V* (Å^3^)	764.64(13)	1678.78(13)	1645.88(16)	739.0(3)	824.2(4)
*d*_calc_ (g·cm^–3^)	1.5462	1.5511	1.5821	1.6216	1.5992
*R*[*F*] (*I* > 3σ(*I*))	5.07%	4.36%	11.73%	3.33%	5.46%
*R*[*F*] (all data)	8.09%	6.54%	17.23%	3.60%	6.43%
ϱ_res_^min/max^ (e·Å^–3^)	–0.47/+0.65	–0.53/+0.42	–1.15/+3.36	–0.38/+0.48	–0.78/+0.98
CCDC code	2110815	2110827	2110828	2110808	2110809

a For more details, see the Supporting Information.

b From the **Ni-2a-100 K-dark-cooling-xtal-1** data set.

c From the **Ni-2b-RT-dark-xtal-2** data set.

d From the **Ni-2b-160 K-dark-xtal-2** data set.

Molecular structures of the studied compounds are illustrated in Figure 1. Both the 2-picolylamine-based (**P**) and 8-aminoquinoline-based (**Q**) ligands coordinate to the metal center via all the electron-donating atoms they possess, i.e., the N3 and bridging N2 nitrogen atoms and the O3 oxygen atom from the carbonyl group. The main difference between the Ni and Cu analogues that is due to the metal center type is the preferred binding mode of the nitro group. The analyzed nickel complexes exist majorly as the nitro form (η^1^-N(O)_2_), whereas their copper equivalents exist exclusively as the κ-nitrito linkage isomer (η^2^-O,ON) in the ground-state crystal structure. These findings are in agreement with the previous literature reports.^15,25,41,72,73^ It should also be noted here that solely in the case of the **Ni-2a** crystal structure do two isomeric forms, namely nitro and *endo*-nitrito (η^1^-ONO), coexist with 92(1)% and 8(1)% occupancies at 100 K, respectively (keeping in mind that no 100 K data were collected for **Ni-2b** due to crystal degradation). In order to verify the impact of temperature on the relative populations of both linkage isomers, multi-temperature X-ray diffraction experiments were performed. The obtained results are gathered in Table 2. It appears that the *endo*-nitrito form is detectable at 180 K and below, while its population reaches a maximum (ca. 11–12%) around 120–160 K. Comparable results were obtained while both cooling and heating the crystal. The presence of the *endo*-nitrito isomer at some range of low temperatures was already observed by us for some other nickel nitro complexes.^15,16^

**Table 2:** *endo*-Nitrito Isomer Populations (*P*) and Reaction-Cavity Volumes (*V*_cav_) per Complex Molecule Calculated for the **Ni-2a** Crystal Structure during Cooling and Heating Experiments^a^

	*T* (K)	*P* (%)	*V*_cav_ (Å^3^)
cooling	290	0.0	42.4
	240	0.0	41.0
	200	0.0	39.8
	160	8(1)	40.0
	140	11(1)	40.1
	120	11(1)	38.0
	100	8(1)	38.4
heating	120	12(1)	38.1
	140	11(1)	39.3
	160	12(1)	40.7
	180	10(1)	40.0
	200	0.0	40.3

a Cavity volumes were computed with the *MERCURY* program^74^ (probe radius of 1.2 Å and grid spacing of 0.1 Å). Note that the standard deviation on *V*_cav_ was estimated to be ca. 0.5 Å^3^.^16^

**Figure 2:**
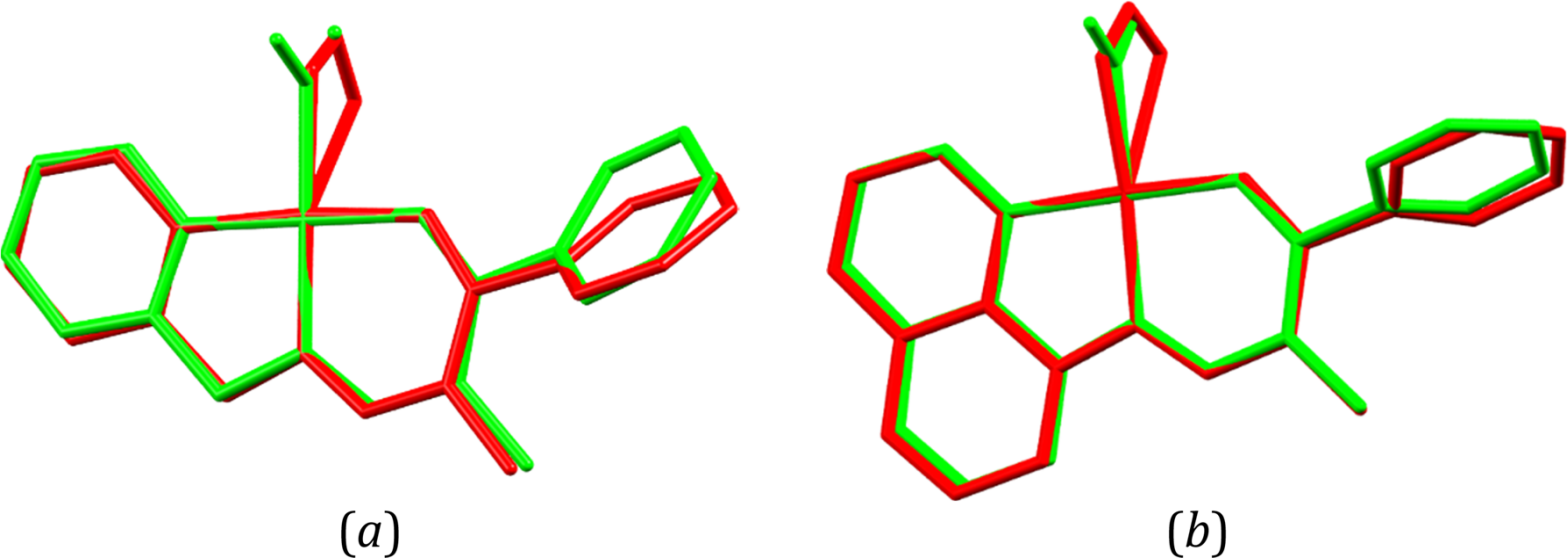
Overlay of selected experimental molecular geometries taken from (a) the crystal structures of **Ni-2a** at 100 K (green) and **Cu-2a** at 100 K (red) and (b) the crystal structures of **Ni-2b** at 290 K (green) and **Cu-2b** at 100 K (red). Hydrogen atoms and the disorder of the nitro group in the crystal structure of **Ni-2a** have been omitted for clarity. The N2, N3, O3, and metal atoms were superimposed using the least-squares procedure implemented in the *MERCURY* program.

In general, analogous molecules of Ni and Cu are similar in terms of their geometries, as indicated by the overlay of the respective moieties presented in Figure 2. Despite the NO_2_ group’s different ground-state binding modes characteristic of the Ni and Cu metal centers and its possible different orientation with respect to the stiff amine fragment’s plane, the only significant geometrical changes concern the angle between the planes based on the rotated six-membered aromatic ring of the aldehyde fragment and the amine moiety. For the **Ni-2a** and **Cu-2a** pair of compounds, the angle values are significantly different (43.37 versus 57.54°, respectively), whereas they are much more comparable for **Ni-2b** and **Cu-2b**, i.e., they adopt values between 61 and 65° for **Cu-2b**, the RT **Ni-2b** polymorph, and molecule B from the low-temperature **Ni-2b** form. In the latter case, molecule A differs the most from the **2b** group, with the discussed angle being equal to 70.04°. Nevertheless, it seems that the rotation of the phenyl ring is mainly governed by the presence of the methyl substituent and secondarily affected by the confining forces of the crystal.

**Figure 3:**
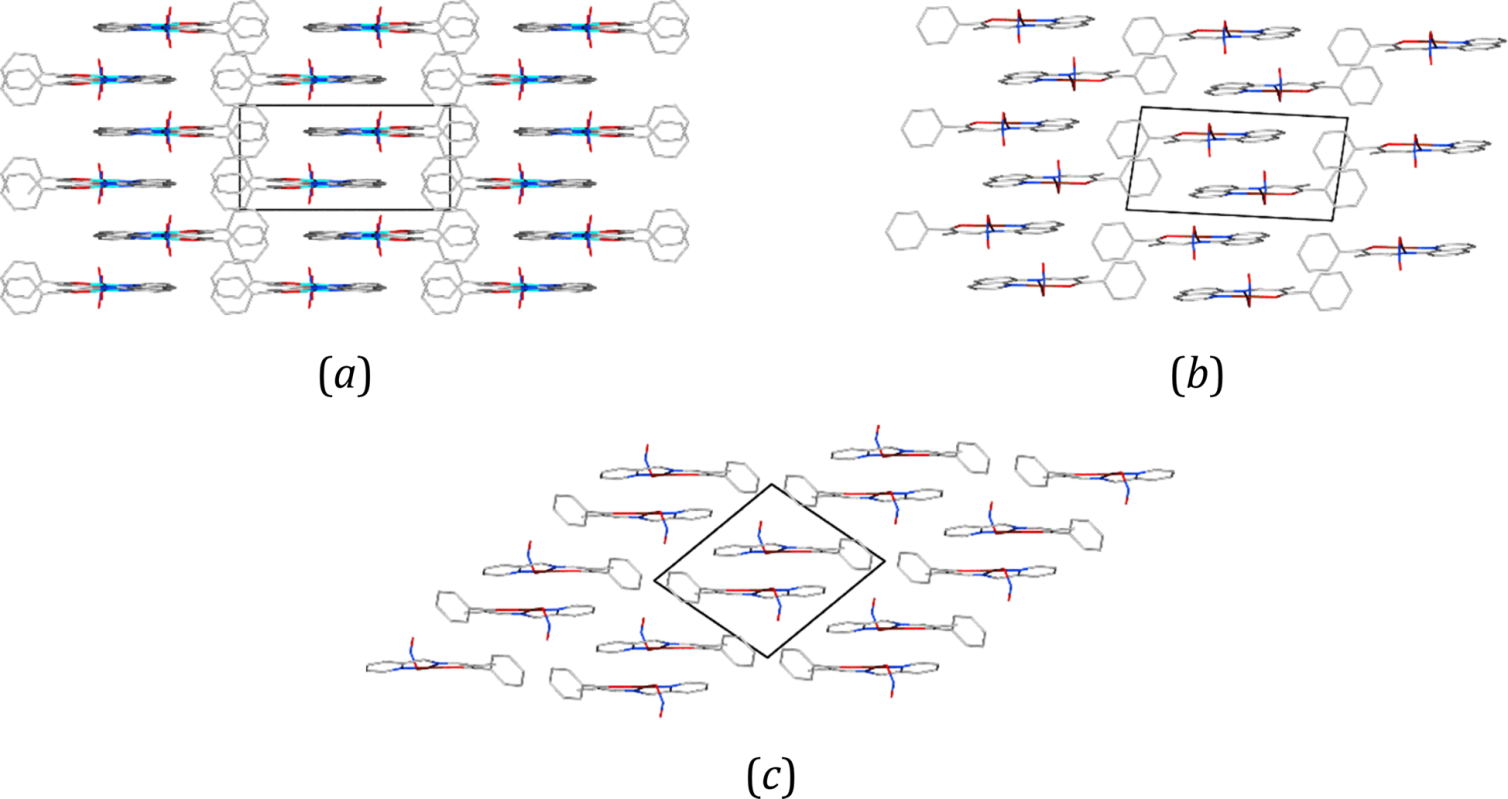
Schematic representation of the ladder-like patterns in the crystal structures of (a) **Ni-2b** (viewed along the *z*-axis), (b) **Cu-2b** (viewed along the *y*-axis), and (*c*) **Cu-2a** (viewed along the *x*-axis).

In three of the examined crystal structures, it appears that molecules with alternating orientations form characteristic ladder-like patterns. Solely in the **Cu-2a** crystal structure in which there are strongly bound centrosymmetric molecular dimers, molecules pack somewhat differently. Nevertheless, in all the systems molecules are arranged parallel one to another, as shown schematically in Figure 3. They interact mainly via effective π···π stacking interactions formed between either the **Q** or **P** aromatic rings (the distance between the ring centroids ranges from 3.6 to 3.8 Å) as well as through hydrogen-bond-like C–H···O interactions involving the NO_2_ moiety or, specific for the Cu compounds, C–H···N contacts of this kind. Additionally, some weaker π···π interactions between phenyl rings may appear. Overall, it seems that varying the metal atom type (Cu versus Ni) or the amine ligand (**P** versus **Q**) does not affect the crystal packing much. General features are very much alike, whereas the more remarkable differences are visible at the level of formed dimeric motifs and the nitro group interactions that result from the adopted binding modes.

**Figure 4:**
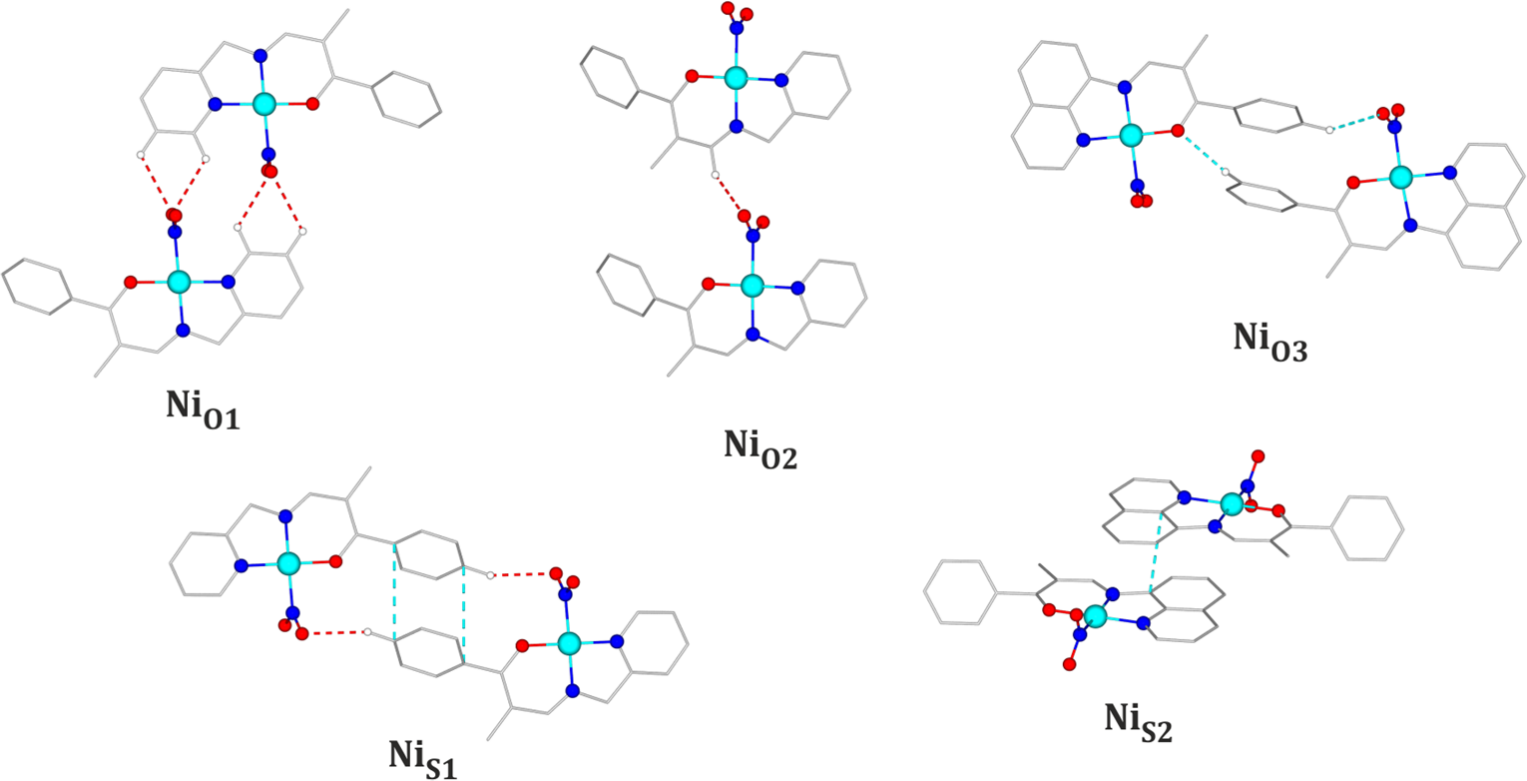
Main structural motifs encountered in the **Ni-2a** and **Ni-2b** crystal structures (for clarity, only the nitro isomer is shown).

Hence, the most significant dimeric motifs present in the analyzed crystal structures were selected, and their structures are shown in Figures 4 and 6. The corresponding geometrical parameters and interaction energy values are gathered in Table 3. As far as the **Ni-2a** crystal structure is concerned, in the case of the dominating nitro isomer, the C1–H1···O1a, C2–H2···O1a, and C7–H7···O2a hydrogen-bond-like interactions can be distinguished. The interaction energies stabilizing the corresponding molecular motifs amount to −61.0 kJ·mol^–1^ for **Ni**_**O1**_, which is held by the first two of the mentioned interactions, and −22.8 kJ·mol^–1^ for the **Ni**_**O2**_ motif, which is linked by the last contact (Figure 3). In the case of the *endo*-nitrito isomer, the nitro group coordinates to the metallic center through the O2b atom. Hence, the ONO moiety forms hydrogen-bond-like interactions mainly via the O1b atom (corresponding to the O1a atom) and N1b (new interactions C1–H1···N1b and C2–H2···N1b), which is more exposed in the *endo*-nitrito binding mode. The resulting **Ni**_**O1**_ motif is thus characterized by the relatively less beneficial interaction energy value when compared to that of its nitro analogue (Table 3). In turn, in the case of the less-populated linkage isomer, the respective dimer stabilization energy is reduced only by 3 kJ·mol^–1^ despite the fact that the C7–H7···O2a hydrogen-bond-like interaction in the **N**_**O2**_ motif is not formed. Otherwise, the crystal structure is stabilized by the previously mentioned π···π interactions, which lead to the most energetically stable dimers **Ni**_**S2**_ and **Ni**_**S2′**_ (interaction energies exceeding −118 and −136 kJ·mol^–1^, respectively) and the well-stabilized **Ni**_**S1**_ motif (Figure 3), which are additionally supported by lateral C–H···O contacts. Similar to the case of **Ni**_**O1**_ and **N**_**O2**_, these dimers, apart from **Ni**_**S2′**_, are less energetically advantageous for the *endo*-nitrito form (Table 3). Overall, as expected, the dominating nitro isomer seems to be interact more effectively in the crystal structure than its *endo*-nitrito equivalent. Despite the limitations of the computational method used, it should also be noted that due to the moderate population of the latter linkage isomer its geometry is evaluated less reliably than that of the nitro form, which may to some extent affect the computational results.

**Table 3:** Selected Dimeric Motifs Present in the Examined Crystal Structures: Geometrical Parameters and Interaction Energy Values (*E*_int_)^a^

**Ni-2a**^b^						**Ni-2b**^c^^,^^d^ (triclinic, low *T*)					
motif	*E*_int_ (kJ·mol^–1^)	selected interactions^e^	*d*_H···A_ (Å)	*d*_D···A_ or *d*_π···π_ (Å)	θ_D–H···A_ (°)	motif	*E*_int_ (kJ·mol^–1^)	selected interactions^e^	*d*_H···A_ (Å)	*d*_D···A_ or *d*_π···π_ (Å)	θ_D–H···A_ (°)
**Ni**_**O1**_	–61.0^f^/–54.0^g^	C1–H1···O1a^1^	2.55	3.196(5)	124.65	**Ni**_**O1**_	–39.6	C2–H2···O4	2.74	3.233(12)	112.70
		C2–H2···O1a^#^	2.66	3.237(6)	118.93			C2′–H2′···O2	2.64	3.139(15)	112.80
**Ni**_**O2**_	–22.8^f^/–19.6^g^^,^^h^	C7–H7···O2a^2^	2.55	3.180(6)	122.97	**Ni**_**O3**_	–44.9	C13–H13···O3′ ^6^	2.73	3.575(13)	146.41
**Ni**_**S1**_	–46.4^f^/–41.5^g^	C14–H14···O1a^3^	2.60	4.059(7)	156.88			C15–H15···O4^6^	2.76	3.586(18)	143.92
		C11(π)···C14(π)^3^		3.838(7)		**Ni**_**O3′**_	–44.6	C13′–H13′···O3^7^	2.90	3.638(16)	134.86
**Ni**_**S2**_	–126.0^f^/–118.9^g^	C4–H4···O1a^4^	2.70	3.143(6)	108.70			C15′–H15′···O2^7^	2.55	3.414(18)	148.91
		C6–H6b···O1a^4^	2.39	3.265(5)	151.32	**Ni**_**S2**_	–95.1	C5(π)···C6(π)^8^		3.362(12)	
		C1(π)···C1(π)^4^		3.561		**Ni**_**S2′**_	–94.8	C5′(π)···C6′(π)^8^		3.373(12)	
**Ni**_**S2′**_	–136.8^f^/–143.5^g^	C6–H6a···O2a^5^	2.54	3.315(6)	137.36						
		C1(π)···C8(π)^5^		3.561(6)							

**Cu-2a**						**Cu-2b**					
motif	*E*_int_ (kJ·mol^–1^)	selected interactions^e^	*d*_H···A_ (Å)	*d*_D···A_ or *d*_π···π_ (Å)	θ_D–H···A_ (°)	motif	*E*_int_ (kJ·mol^–1^)	selected interactions^e^	*d*_H···A_ (Å)	*d*_D···A_ or *d*_π···π_ (Å)	θ_D–H···A_ (°)
**Cu**_**N1**_	–35.0	C4–H4···N1^9^	2.72	3.574(4)	148.78	**Cu**_**O1′**_	–34.4	C1–H1···O2^5^	3.02	3.210(5)	92.33
**Cu**_**C1**_	–61.7	C7–H7···H12^10^		2.89^i^		**Cu**_**O1**_	–46.1	C2–H2···O1^4^	2.70	3.381(5)	128.67
**Cu**_**C2**_	–30.0	C12–H12···H12^11^		2.84^i^		**Cu**_**N1**_	–25.9	C7–H7···N1^2^	2.75	3.686(5)	164.51
**Cu**_**S3**_	–60.2	C3–H3···N1^12^	2.74	3.465(3)	132.97	**Cu**_**S1**_	–38.4	C14–H14···O2^14^	2.70	3.511(6)	142.06
		C1(π)···C1(π)^12^		3.284(3)		**Cu**_**S2**_	–100.2	C6(π)···C6(π)^8^		3.213(5)	
**Cu**_**Cu**_	–110.9	Cu–O1···N1^13^		2.582(2)^j^		**Cu**_**S3**_	–51.9	C16(π)···C17(π)^3^		3.694(7)	

a Calculated at the DFT(B3LYP)/6-311++G** level of theory with the Grimme dispersion correction applied. Geometries with the X–H neutron-normalized distances were used for computations.

b From the **Ni-2a-100 K-dark-cooling-xtal-1** data set.

c From the **Ni-2b-160 K-dark-xtal-2** data set.

d The A–B dimers are referred to with the base symbols, while the respective B–A dimers are referred to with the primed symbols.

e It should be noted that the given interaction energy values describe the total interaction between the two molecules comprising the structural motif, i.e., they include not only the presented hydrogen-bond(-like) contacts but also some other weak interactions that stabilize the system.

f Values for homodimers consisting of both molecules in the nitro form.

g Values for homodimers consisting of both molecules in the *endo*-nitro form.

h The same motif consisting of two MS (metastable state) linkage isomers, though in this case the C7–H7···O2a interaction is not present.

i H···H contact.

j The Cu–O coordination bond length. Symmetry operations are indicated as superscripts and defined as follows: (1) −*x* + 2, −*y* + 3, −*z* + 1; (2) *x*, *y* – 1, *z*; (3) −*x* + 1, −*y* + 2, −*z*; (4) −*x* + 2, −*y* + 2, −*z* + 1; (5) −*x* + 1, −*y* + 2, −*z* + 1; (6) *x* + 1, *y* + 1, *z*; (7) *x* + 1, *y*, *z*; (8) −*x* + 1, −*y* + 1, −*z* + 1; (9) *x* – 1, *y*, *z*; (10) −*x* + 1, −*y* + 1, −*z*; (11) −*x*, −*y* + 1, −*z*; (12) −*x*, −*y* + 2, −*z* + 1; (13) −*x*, −*y* + 1, −*z* + 1; and (14) *x*, *y*, *z* – 1.

**Figure 5:**
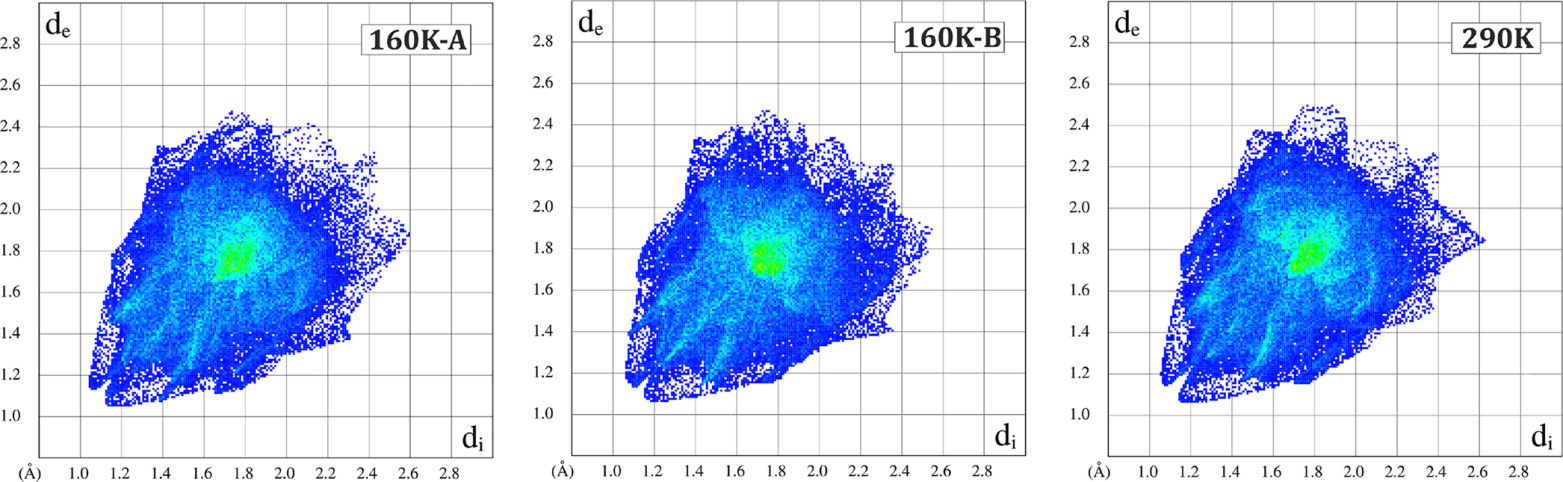
Hirshfeld surface fingerprint plots^75^ generated for both the 160 K **Ni-2b** crystal structure for molecules A (left panel) and B (middle panel) and the RT **Ni-2b** crystal structure (right panel). Plots were generated using the *CRYSTALEXPLORER* software.^76^

In turn, in the **Ni-2b** crystal structure, the nitro group adopts only the nitro binding mode throughout the 160–290 K temperature range. Furthermore, it should be noted that no significant differences were observed between various contact types and their percentage contributions when both molecules in the asymmetric unit of the **Ni-2b** structure were compared (Tables 3 and 6S) in addition to the two **Ni-2b** polymorphs (Table 6S), which is well illustrated by the very similar interatomic contact patterns in the respective fingerprint plots in Figure 5 (Table 7S). This shows that the phase transition does not change the nature and number of the interatomic contacts in the crystal structure very much and thus should not affect the NO_2_ isomerization potential. The crystal structure is again stabilized by effective π···π interactions (motifs **Ni**_**S2**_ and **Ni**_**S2′**_) encountered for both molecules in the ASU (the stabilization energies amount to −95.1 and −94.8 kJ·mol^–1^, respectively). Regarding the nitro moiety, the centrosymmetric **Ni**_**O1**_ motif (−39.6 kJ·mol^–1^) linked via the C2–H2···O4 and C2′–H2′···O2 hydrogen-bond-like interactions should be distinguished. In this view, it is also worth mentioning that the **Ni**_**O2**_ motif is not formed in the **Ni-2b** crystal structure and the the π···π stacking contacts between the phenyl rings are not observed (**Ni**_**S1**_ in **Ni-2a**). Instead, the **Ni**_**O3**_ motif, which is stabilized by the C15–H15···O4 and C13′–H13′···O3 interactions, and its **Ni**_**O3′**_ analogue are created. **Ni**_**O3**_, **Ni**_**O3′**_, and **Ni**_**S1**_ are characterized by very comparable interaction energy values (around −45 kJ·mol^–1^). Nonetheless, it seems that the nitro group in **Ni-2b** forms somewhat weaker intermolecular interactions than that in **Ni-2a**.

**Figure 6:**
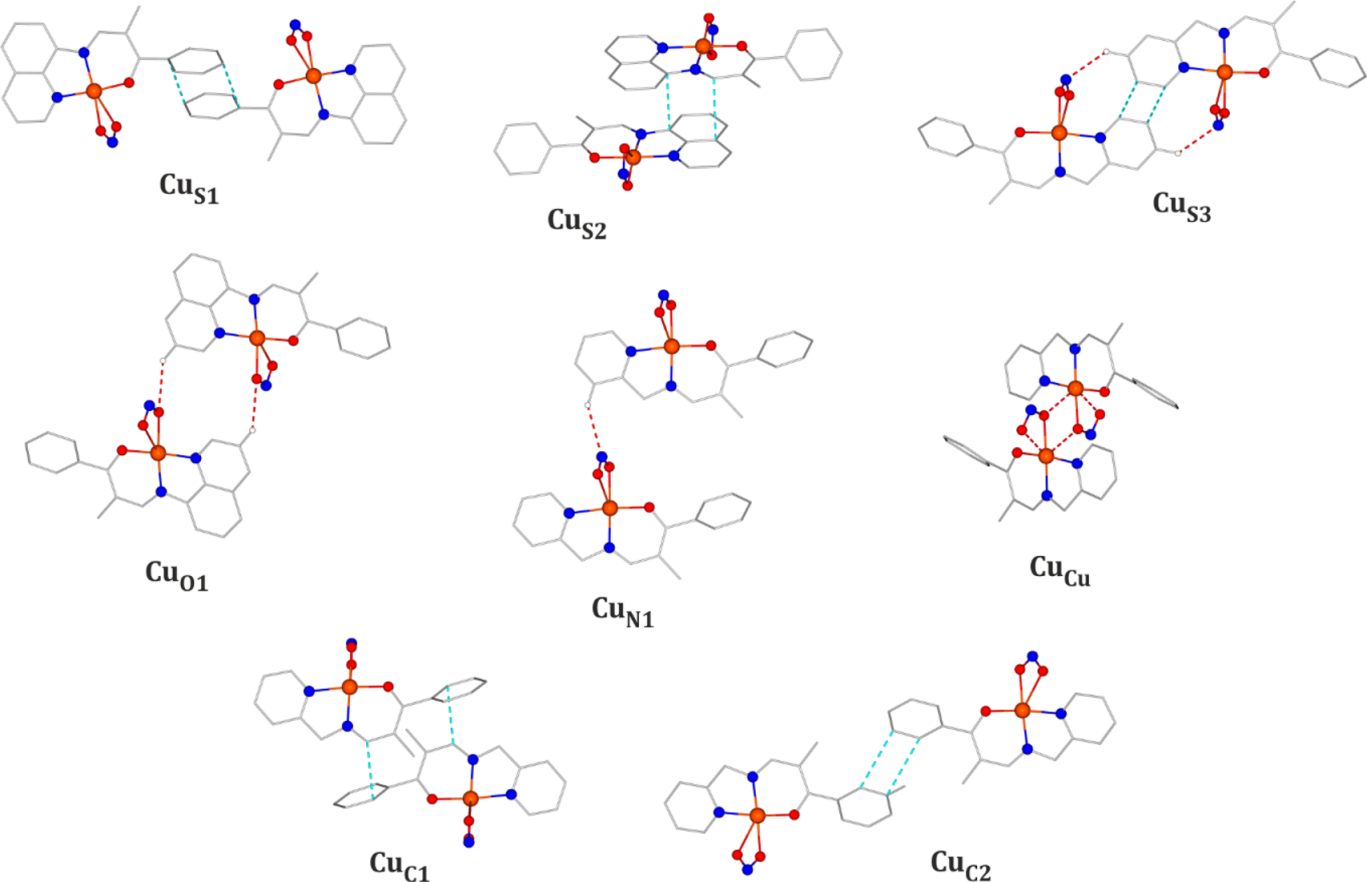
Main structural motifs encountered in the **Cu-2a** and **Cu-2b** crystal structures.

Although the intermolecular interactions in the crystal structures seem to be rather similar for all the studied compounds, several remarkable differences were noticed for the **Cu-2a** complex. First, the complex molecules here form a strongly stabilized **Cu**_**Cu**_ dimeric motif (Figure 6) in which the two moieties are bound together via the Cu1···O1 coordination-bond-like interactions. The dimer stabilization energy reaches −110.9 kJ·mol^–1^. Other than that, the crystal structure of **Cu-2a** is stabilized mainly by multiple weak hydrogen-bond-like interactions, such as C4–H4···N1 (**Cu**_**N1**_ motif), and a centrosymmetric pair of C3–H3···N1 (**Cu**_**S1**_ motif) contacts; the total interaction energies of the components of the motifs are −35.0 and −60.2 kJ·mol^–1^, respectively. Interestingly, the **Cu-2a** complex is distinguished by having the highest percentage contribution of C–H contacts to the Hirshfeld surface (Table 6S) among the studied systems. This is a result of the mutual orientation of the complex molecules leading to favorable edge-to-face-like interactions (C–H···π) between the C7–H7 bond dipole and the phenyl ring. Such interactions link the complex molecules in the **Cu**_**C1**_ centrosymmetric motif, which is characterized by a stabilization energy of −61.7 kJ·mol^–1^. Finally, it is worth mentioning that the remaining motif, **Cu**_**C2**_, with the total interaction energy of −30.0 kJ·mol^–1^ is stabilized mainly by weak dispersive interactions between the phenyl rings.

In turn, concerning the **Cu-2b** system, the C1–H1···O2, C2–H2···O1, and C7–H7···N1 hydrogen-bond-like interactions involving the nitro group should be noted. The total interaction energies of motifs stabilized mainly by these contacts amount to −34.4 (**Cu**_**O2**_ motif), −46.1 (**Cu**_**O1**_ motif), and −25.9 kJ·mol^–1^ (**Cu**_**N1**_ motif). The **Cu**_**S1**_ motif constitutes the most energetically stable dimer (−51.9 kJ·mol^–1^) among those supported by interactions engaging the NO_2_ group. It is stabilized by a centrosymmetric pair of C14–H14···02 interactions along with the π···π stacking contacts between the phenyl rings. It is also worth considering the best-stabilized **Cu**_**S2**_ motif (−100.0 kJ·mol^–1^ total energy) with a significant contribution of the π···π stacking interaction between the **Q** ligand rings. Here, no edge-to-face-like interactions were identified.

As far as the photoactivity of the nitro group is concerned, two factors regarding the crystal packing are of great importance. These are the cavity volume and the intermolecular interactions involving the ambidentate ligand. Indeed, it is essential to ensure the space necessary for the NO_2_ group to undergo light-induced linkage isomerization.^8,15−18,22^ Naturally, not only the size of the cavity but also its shape are relevant so as to ensure the smallest possible changes in the unit cell upon the molecular transformation.^19^ Hence, the reaction cavity volumes were calculated for all examined crystal structures using the *MERCURY* software (with the rolling-probe method with a probe radius of 1.2 Å and a grid spacing of 0.1 Å)^74^ and analyzed. The respective results are shown in Table 4. It appears that there is no strict correlation between the metallic center type and reaction cavity volume. The largest values were obtained for the **Ni-2a** crystal structure in which two isomers are already present at 100 K and for the **Cu-2a** system, which forms a strongly stabilized dimer in the crystal structure via interactions between two ONO species (Figure 6). Provided that in the case of **Ni-2a** a mixture of the nitro and *endo*-nitrito forms exists at temperatures below 200 K, it is highly probably that this system is converted to the nitrito linkage isomer upon light irradiation to a higher degree. Importantly, all the obtained values are comparable to those derived for the previously reported crystal structures of nickel complexes exhibiting nitro-to-nitrito photoisomerism.^15,16,21^

**Table 4:** Reaction-Cavity Volumes (*V*_cav_) per Complex Molecule Calculated for the Ground-State Crystal Structures^a^

compound	*V*_caav_ (Å^3^)
**Ni-2a**	38.4^b^
**Ni-2b**	27.7^c^/32.7^d^/34.9^e^
**Cu-2a**	37.1^b^
**Cu-2b**	33.6^b^

a Computed with the *MERCURY* program (probe radius of 1.2 Å and grid spacing of 0.1 Å). Note that the standard deviation on *V*_cav_ was estimated to be ca. 0.5 Å.^16^

b Data were collected at 100 K.

c Symmetry-independent molecule A (data were collected at 160 K).

d Symmetry-independent molecule B (data were collected at 160 K).

e Data were collected at room temperature (only one molecule in the ASU).

Concerning the interactions involving the nitro moiety, to facilitate the desired chemical transformation, they should contribute to the crystal stability but should not be too strong. This can be to some extent resolved using relatively bulky chelating ligands, which should not form strong hydrogen bonds with the ambidentate species.^8,20,77^ In the studied case, the developed amine fragment seems to fulfill such criteria, whereas the ONO group is usually involved in a few medium-strength hydrogen-bond-like interactions. For the examined nickel coordination compounds, these are solely the C–H···O type contacts, whereas C–H···N interactions additionally appear in the case of the κ-nitrito copper complexes. As described above, and indicated in Table 3, these hydrogen-bond-like interactions are in general characterized by the energy values ranging from about −25 to about −30 kJ·mol^–1^, which on average are slightly less advantageous for copper systems. Additionally, it should be noted here that the more volumetric **Q** ligands in the case of the **2b** systems indeed contribute to some weakening of the interactions involving the ONO fragment with respect to their **2a** analogues.

Overall, it seems that the **2a** group is characterized by greater cavity volumes but slightly stronger interactions engaging the ONO moiety in the crystal structure compared to those of the **2b** anaolgues. Nevertheless, based on these findings, all the examined compounds have the potential to give a positive response to the laser light and undergo the isomerization reaction under some specific conditions.

### 3.2 Solid-State IR Spectroscopy

**Figure 7:**
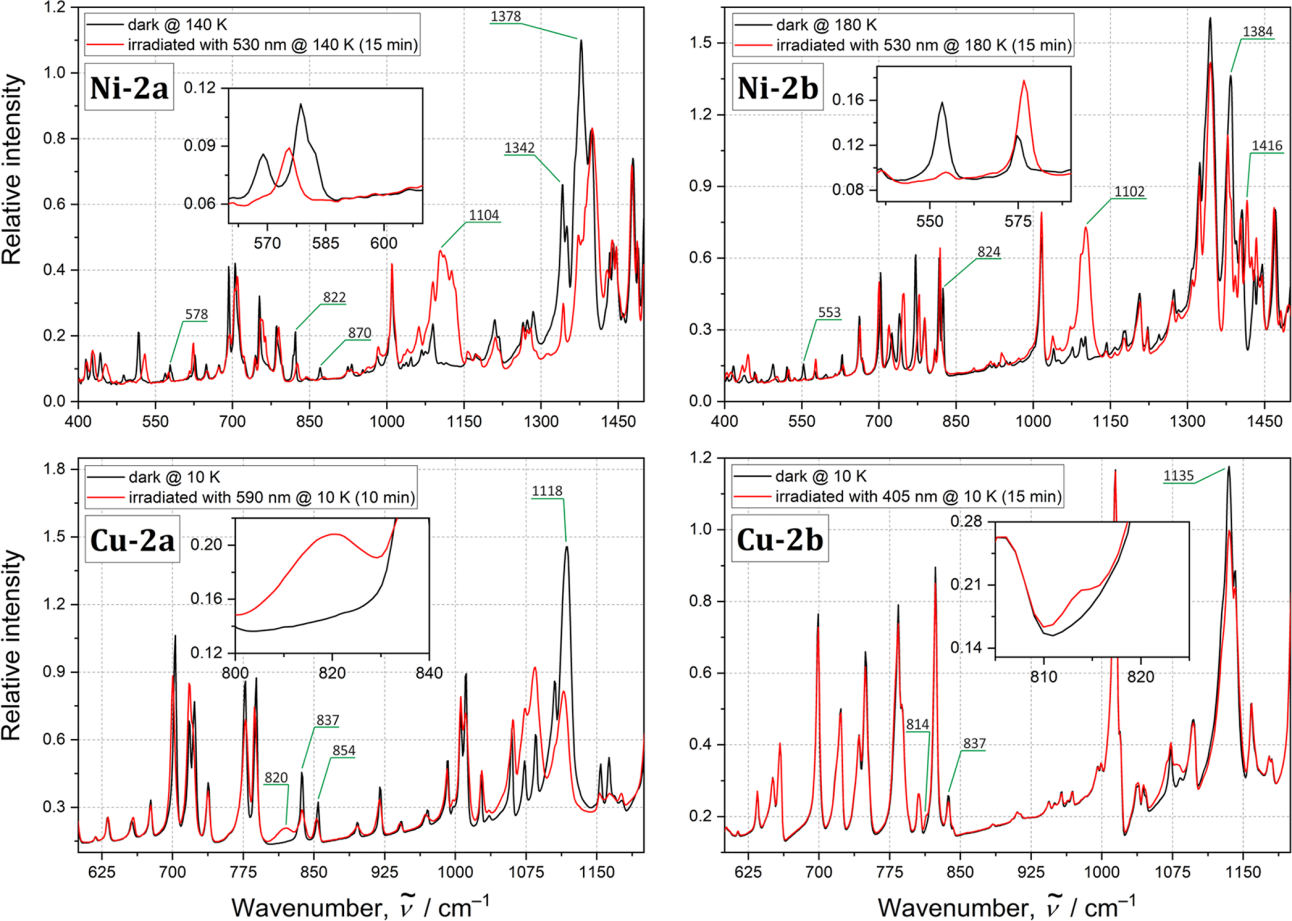
IR absorption spectra for all studied complexes in the solid state. Black lines denote the ground-state spectra, while red lines denote spectra recorded after 10 min of LED irradiation. For more information and difference spectra, see the Supporting Information.

Solid-state infrared (IR) multi-temperature spectroscopic experiments, also performed after the sample was irradiated with LED light, were conducted to determine the optimal isomerization reaction conditions for each of the four systems under consideration and to estimate the achievable conversion in the case of thin-film samples (their purity and composition were confirmed by elemental analyses and PXRD patterns; for the latter, see the Supporting Information). Special attention was paid to vibrational bands present in the 500–850 and 1000–1500 cm^–1^ spectral ranges, as according to literature crucial vibrations of the ONO species should appear in these regions.^15,73,78−84^ The obtained experimental results were supported by theoretically predicted normal-mode frequencies computed at the DFT(B3LYP)/6-311++G** level of theory (Figures 6S and 7S and Table 8S).

**Figure 8:**
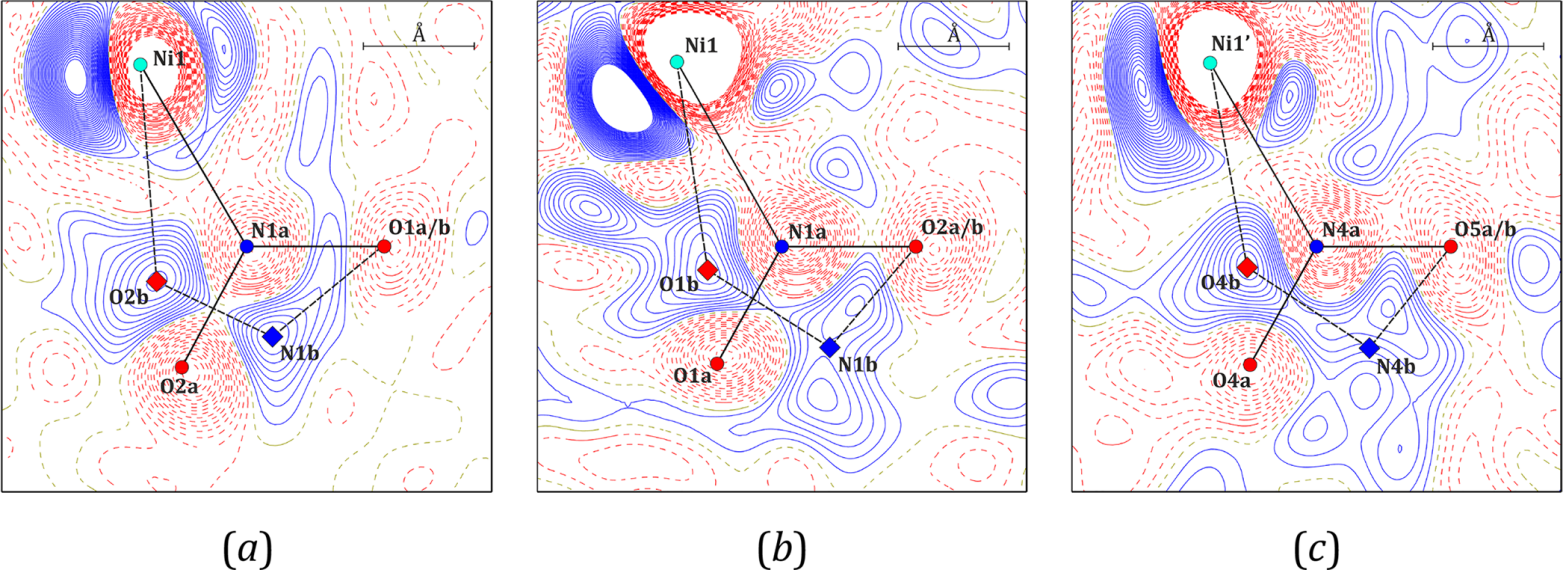
Photodifference Fourier maps (*F*^ON^–*F*^OFF^) indicating the coexistence of both nitro (−N(O)_2_) and *endo*-nitrito (−ONO) linkage isomers for (a) **Ni-2a** and two symmetry-independent molecules of **Ni-2b**, namely (b) molecule A and (c) molecule B. Solid blue lines represent positive values, dashed red lines represent negative values, and contours are at ±0.2 e·Å^–3^.

**Figure 9:**
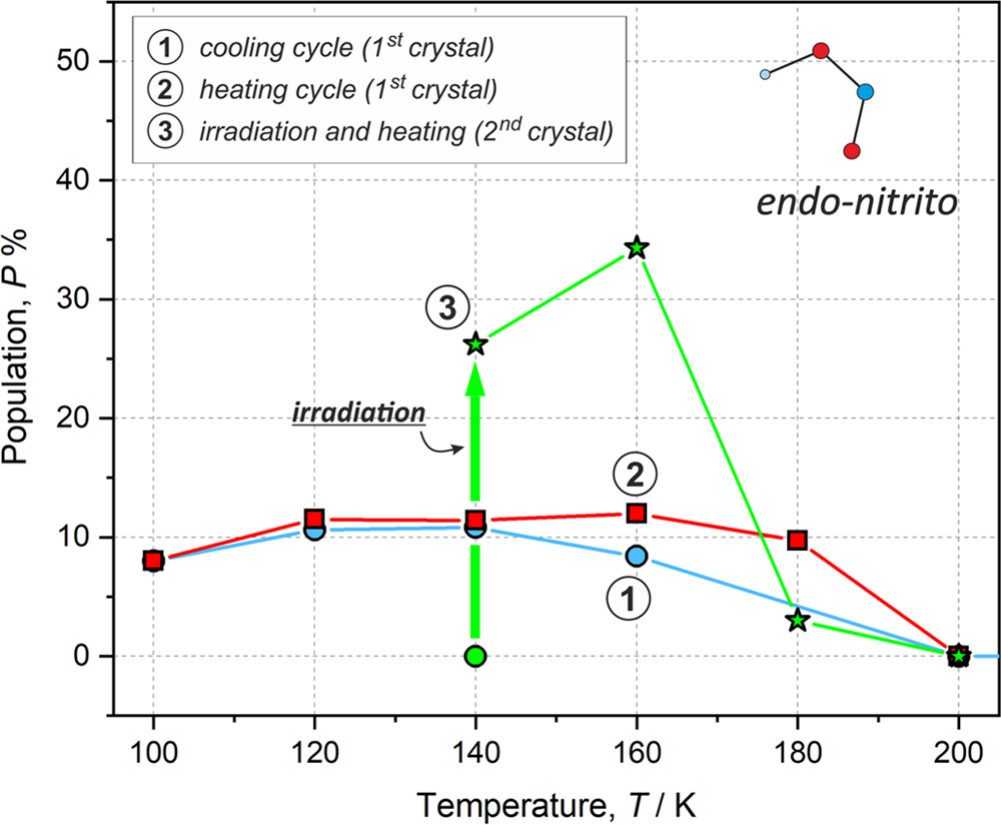
Populations (*P*) of the metastable linkage isomer (*endo*-nitrito form) for the **Ni-2a** complex observed during the multi-temperature (cooling and heating) and photocrystallographic experiments. Blue circles denote data from the cooling experiment, red squares denote data from the heating experiment, and green stars denote data from the heating experiment after LED irradiation (denoted schematically with a thick green arrow; the 140 K structure before irradiation is shown as a green circle). Note that a different crystal was used for the photocrystallographic experiment than that used in the multi-temperature measurements.

When the temperature was decreased from RT to 10 K, only some minor spectral changes were detected for the examined samples (Figures 8S and 9S), indicating the low efficiency of the thermally induced nitro-to-nitrito isomerization reaction. As expected, the most visible, but still rather moderate, temperature effects were noted for the **Ni-2a** thin-film sample, which is in agreement with the single-crystal X-ray diffraction experimental findings.

In turn, significant spectral changes were observed upon UV–vis light irradiation, as illustrated in Figure 7, and in the difference spectra in the Supporting Information (Figures 12S–19S). As far as the nickel systems are concerned, the most noticeable effects in the IR spectrum were generated by the LED light in the 405–530 nm wavelength range (at 10–200 K) for **Ni-2a,** while for **Ni-2b** the effective wavelength range was red-shifted. After the sample was exposed to the 530 nm LED light at 140 K, the 569–578 cm^–1^ band for **Ni-2a** (553 cm^–1^ for **Ni-2b** at 180 K, in parentheses hereafter) assigned to the ω(NO_2_) wagging vibration mode almost completely disappeared. Furthermore, the intensity of the 822 (824) cm^–1^ band, which can be attributed to the δ(NO_2_) scissoring vibration, decreases for **Ni-2a** (the band remains almost unshifted at 826 cm^–1^) and nearly vanishes for **Ni-2b**. In the case of **Ni-2a**, however, a further band at 870 cm^–1^ almost disappears upon irradiation, so it is not obvious which of the 822 and 870 cm^–1^ bands corresponds to the δ(NO_2_) mode. The identification of new bands that arise along with the generation of the *endo*-nitrito form in the 400–1000 cm^–1^ spectral region is not unambiguous either due to the significant shifts of several bands. These shifts are caused by the overall structural adaptation of the molecules induced by the NO_2_ isomerization. In the spectral 1000–1550 cm^–1^ spectral region, one can observe the intensity decrease of the symmetric ν_s_(NO_2_) and asymmetric ν_as_(NO_2_) stretching vibrations, located at 1342 (1333) and 1378 (1384) cm^–1^, respectively, upon isomerization. In turn, new bands arise at 1104 (1102) and around 1408 (1416) cm^–1^ that correspond to the ν(N–O) and ν(N=O) stretching vibrations of the nitrito binding mode, respectively. Such spectral behavior is characteristic of the nitro-to-nitrito transformation,^15,85−87^ which has been confirmed by the theoretical computation of vibrational frequencies for the respective linkage isomers (Table 8S). On the basis of the observed spectral changes, the highest achieved level of conversion for the **Ni-2a** and **Ni-2b** complexes can be estimated to about 95–100% and 90%, respectively, whereas the nitrito form was present in the samples below 220 K. The optimal isomerization reaction conditions are presented in Table 5.

**Table 5:** Optimal Isomerization Reaction Conditions for the Studied Systems in the Solid State Based On the IR Spectroscopy Results^a^

compound	λ_irr_ (nm)	*T*_irr_ (K)	*T*_rev_ (K)
**Ni-2a**	470–530	10–140	220
**Ni-2b**	530	180	220
**Cu-2a**	590	10	60
**Cu-2b**	405	10	80

a Symbols are defined as follows: λ_irr_, the most efficient LED central wavelength regarding the generation of the metastable linkage isomer; *T*_irr_, the temperature at which the metastable linkage isomer’s population is the highest; and *T*_rev_, the temperature at which the system reverts back to the ground state.

In the case of the copper complexes, after the 405–660 nm LED light irradiation at 10 K, the 837–854 cm^–1^ bands for the **Cu-2a** complex (405–590 nm LED light; 837 cm^–1^ band for **Cu-2b**, in parentheses hereafter) partially disappear. This band is assigned to the δ(ONO) mode of the κ-nitrito configuration. Furthermore, a new band emerges around 820 (815) cm^–1^, which is associated with the scissoring δ(NO_2_) vibration characteristic of the nitro binding mode. Additionally, some shifts and intensity decreases of the bands located at 1118 (1135) and 1402 (1380–1400 range) cm^–1^, corresponding to the ν(N–O) and ν(N=O) stretching vibrations, respectively, were observed. This may result from the decrease of the population of the ground-state κ-nitrito isomer for the benefit of the light-induced nitro linkage isomer. The presumed partial nitrito-to-nitro transformation is supported by the theoretical computations (Table 8S). The effect seems to be rather moderate yet visible. A stronger light-induced effect was observed for the **Cu-2a** thin-film sample, indicating its more efficient isomerization when compared to that of **Cu-2b**. The generated metastable linkage isomer was present up to about 60–80 K.

Importantly, no noticeable sample fatigue was observed and all important spectroscopic features were reproducible, confirming the reversibility of the transformation. The experimentally determined optimal conditions for further photocrystallographic investigations are indicated in Table 5.

### 3.3 Photocrystallography

Since the copper systems work moderately and only below 80 K, while nickel complexes in the solid state are more effective and undergo the isomerization reaction above 100 K, only the nickel compounds were further investigated photocrystallographically. Irradiation wavelengths were initially set to 530 nm (LED central wavelength), and the photoisomerization reaction temperature was set to 140 K for **Ni-2a** and 160 K for **Ni-2b** based on the IR spectroscopy and multi-temperature X-ray diffraction results. However, preliminary photocrystallographic experiments showed that single crystals of **Ni-2a** degraded rather easily under such conditions. It appeared that changing the excitation wavelength to 590 nm facilitates full data collection. Importantly, using the respective LED, relatively efficient photoisomerization was also observed by the solid-state IR spectroscopy technique (conversion at the 70% level versus 95% at 10 K for the 590 and 530 nm LED light irradiation, respectively; based on data from solid-state UV–vis spectroscopy performed at RT, the 590 nm wavelength matches well with the absorption tail of the **Ni-2a** sample). In turn, in the case of **Ni-2b**, the photocrystallographic experiment was conducted at 160 K rather than 180 K to better facilitate disorder-model refinement. It should be stressed here that the IR spectroscopy results show only minor differences in the photoisomerization reaction efficiency between 140 and 180 K. Detailed information on the photocrystallographic experiment strategy is given in the section 2.

In the cases of both **Ni-2a** and **Ni-2b**, when the target temperature was reached before irradiation, only the nitro isomer was present in the crystal structure (for **Ni-2a** a “frozen” ambient structure was obtained due to faster cooling). However, exposing the single crystals of **Ni-2a** and **Ni-2b** to the specified LED light for about 30 and 45 min, respectively, caused a partial transformation from the nitro binding mode to *endo*-nitrito binding mode as shown in Figure 8, which contains the respective photodifference Fourier maps (they show the electron density difference betweenthe light-ON and light-OFF crystal structures). It should be noted that the longer irradiation of both samples caused their degradation. Presumably, non-uniform metastable-state (MS) population build-up leads to significant tensions, thus resulting in the sample decay. Furthermore, no traces of the *exo*-nitrito isomer (η^1^-ONO) reported for similar nickel(II) nitro complexes were detected.^16,20,79^ The population evolution of the linkage isomer along with temperature increase for **Ni-2a** is shown in Figure 9. Additionally, Table 6 gathers the corresponding numerical data, including also the cavity volume changes during the whole experiment for both **Ni-2a** and **Ni-2b**.

**Table 6:** *endo*-Nitrito Linkage Isomer Populations (*P*) and Reaction-Cavity Volumes (*V*_cav_) per Complex Molecule Calculated for the **Ni-2a** Complex during Photocrystallographic Experiments^a^

compound	*T* (K)	*P* (%)	*V*_cav_ (Å^3^)
**Ni-2a**	140^b^	0.0	39.4
	140	26.2(2)	38.8
	160	34.3(3)	33.5
	180	≈3^c^	31.7
	200	0.0	41.09
**Ni-2b**^b^	160^b^	0.0	27.7^d^/32.7^e^
	160	36(1)^d^/37(1)^e^	28.1^d^/30.9^e^
	240	0.0	30.5^d^/34.4^e^

a Calculated before and after irradiation during structure thermal relaxation. Cavity volumes were computed with the *MERCURY* program (probe radius of 1.2 Å and grid spacing of 0.1 Å). Note that several values are provided for the **Ni-2b** crystal structure. Note that the standard deviation on *V*_cav_ was estimated to be ca. 0.5 Å^3^.^16^

b Before irradiation.

c Roughly estimated based on the generated residual density maps.

d Symmetry-independent molecule A.

e Symmetry-independent molecule B.

Based on the refinement of the observed structural disorder, the isomerization reaction conversion level was determined to be ca. 26% for **Ni-2a** and around 36% for both symmetry-independent molecules A and B in **Ni-2b**, respectively. Interestingly, it appeared that in the case of **Ni-2a** it was possible to further increase the population of the *endo*-nitrito form to around 34% by increasing the temperature of the system by 20 to 160 K. The temperature effect is much more pronounced in the photocrystallographic data when compared to that in the multi-temperature results. As far as the **Ni-2a** crystal structure is concerned, the photoinduced isomer was observed below 200 K. Such a result is in agreement with the multi-temperature experiments, which also indicated the full relaxation of the isomerization reaction product at 200 K (Table 6). Similarly, the solid-state IR spectra showed some traces of the metastable isomer up to 220 K at best. The changes of the *endo*-nitrito isomer population with temperature may also suggest that the effect of irradiation vanishes between 160–180 K and solely the temperature effect is further observed. Otherwise, these findings and our further tests show that the process is fully reversible and repeatable.

In the case of the **Ni-2b** crystal structure, the *endo*-nitrito state population achieved after irradiation of the sample at 160 K is comparable to that obtained for **Ni-2a** at the same temperature. Full relaxation of the **Ni-2b** sample to the ground-state (GS) nitro isomer at 240 K was confirmed crystallographically. A detailed examination of the crystal was not possible due to its degradation due to temperature changes and irradiation.

As far as the cavity volume is concerned, there is no strict correlation between its size and the level of conversion. In the case of **Ni-2a**, a decrease in the cavity volume occurs when the metastable form appears. Although this change just after crystal irradiation at 140 K is rather moderate, when the temperature is elevated to 160 K, a significant shrinking of the cavity volume is noted, i.e., from around 39 to 33.5 Å^3^, along with a further 8% increase of the population of the *endo*-nitrito form. The cavity size remains small at 180 K (taking into account both its absolute volume and the size of the unit cell), at which point only the residual metastable state population can be detected. In turn, at 200 K, where the system reverts fully back to the nitro form, the cavity volume expands again, reaching 41 Å^3^. The observed changes seem somewhat delayed with respect to the *endo*-nitrito isomer (MS) population and temperature changes, which might be attributed to the isomerization reaction kinetics and slower geometrical changes of the whole system toward achieving the thermodynamic equilibrium, among others. The shrinkage of the reaction cavity volume with the generation of the MS linkage isomer species constitutes behavior opposite of that earlier reported in the literature.^15−18,20^ Importantly, in these previous studies, the *exo*-nitrito form was observed prior to the *endo*-nitrito isomer. Since the two isomers have different shapes, they pack in the cavity in different ways; thus, they may impose some different structural response when they form. The formation of the more spatially extended *exo*-nitro binding mode may require some more significant structural changes or more space in its closest environment than is the case for a more compact *endo*-nitrito linkage isomer, which further affects the size of the reaction cavity. It should also be noted that for **Ni-2a** the reaction cavity volume changes are much more pronounced after single-crystal sample irradiation than during the multi-temperature X-ray diffraction experiments. It seems that in the latter case the temperature has the primary effect on the reaction cavity volume changes, as these are strongly correlated with thermal changes (expansion and contraction) of the size of the unit cell as a whole (thus, the MS linkage isomer population increase has almost no impact).

In the case of the very alike A and B molecules (both regarding their geometry and intermolecular interactions) in **Ni-2b**, although their respective cavity sizes are different (greater by a few angstroms cubed for molecule B), the increased metastable linkage isomer populations are statistically equal. It should be noted that the cavity volume changes are in general less notable for **Ni-2b** than those observed for **Ni-2a**. After the irradiation of the sample at 160 K, the cavity volume at the A site almost does not change (it moderately increases by about 0.5 Å^3^, which is within the estimated error^16^), while that at the B site decreases by 1.75 Å^3^, which is similar to the cavity volume change noted for **Ni-2a** upon irradiation at 140 K. At 240 K, at which no metastable state is present, the cavity volume is again greater but not to such an extent as that in **Ni-2a**.

### 3.4 Computational Analysis of Nickel(II) Systems

**Figure 10:**
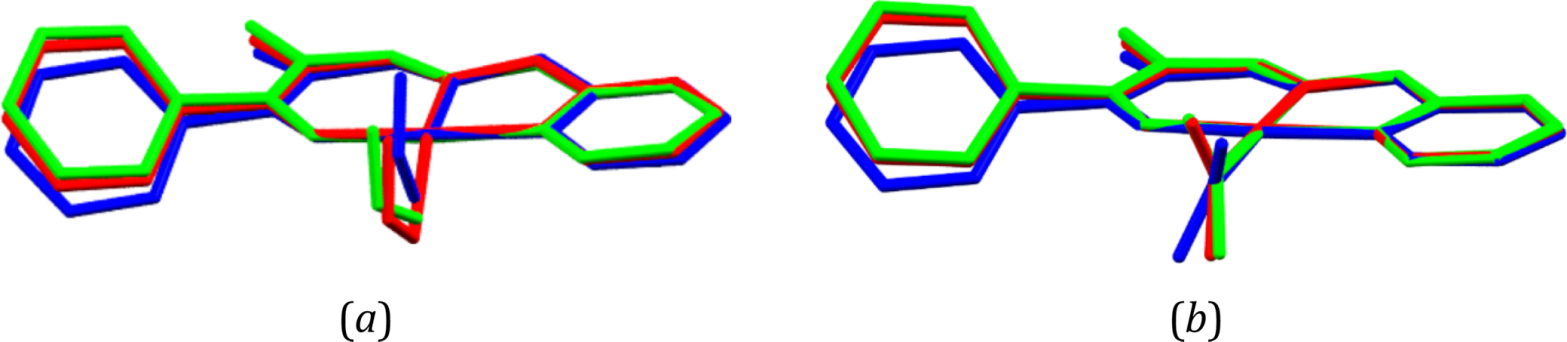
Overlay of molecular geometries obtained from the experiment at 140 K (red), isolated-molecule calculations (blue), and QM/MM calculations (green) for the (a) nitro and (*b*) *endo*-nitrito isomers of the **Ni-2a** complex. Hydrogen atoms have been omitted for clarity. The N2, N3, O3, and metal atoms have been superimposed using the least-squares procedure implemented in the *MERCURY* program.

To gain some more insights into the examined processes and support the experimental observations, theoretical computations were conducted. Therefore, geometry optimizations of the nitro and *endo*-nitrito isomers of **Ni-2a** and **Ni-2b** were performed both for the isolated molecules and using the QM/MM approach, which reflects crystal confining effects well.^15,16,54,88−92^ The latter optimizations were based on the crystal structures after irradiation (collected at 140 K for **Ni-2a** and at 160 K for **Ni-2b**). A comparison of the experimental and optimized molecular geometries is presented in Figure 10, whereas the molecular energy values computed for each geometry complemented by the hypothetical *exo*-nitrito isomer are gathered in Table 7.

**Table 7:** Energy Differences (Δ*E*_rel_) between the Ground (Nitro) and Metastable (*endo*-Nitrito) Linkage Isomers Computed Using the QM/MM Approach and the Optimized Isolated-Molecule Geometries^a^

		Δ*E*_rel_/kJ·mol^–1^	
complex	form	QM/MM	isolated molecule
**Ni-2a**	nitro	0.0	0.0
	*endo*-nitrito	48.4	5.9
	*exo*-nitrito	73.8	14.3
**Ni-2b** (molecule A)	nitro	0.0	0.0
	*endo*-nitrito	15.5	8.2
	*exo*-nitrito	60.3	8.6
**Ni-2b** (molecule B)	nitro	0.0	0.0
	*endo*-nitrito	21.2	8.2
	*exo*-nitrito	41.2	8.6

a Computations were performed at the DFT(B3LYP)/6-311++G** level of theory.

Based on the computational results, the nitro isomer constitutes the most energetically stable form in the case of the studied nickel(II) complexes. In contrast, the *exo*-nitrito isomer is generally the least energetically favorable one. It should be stressed here that, considering solely the isolated-molecule optimization results, it appears that the *endo-* and *exo*-nitrito forms in **Ni-2b** are characterized by almost the same molecular energies. In **Ni-2a**, the difference is more pronounced, but it still does not exceed 10 kJ·mol^–1^. The situation looks diametrically different when the crystal environment is considered. As far as the QM/MM modeling is concerned, the *exo*-nitrito form is less energetically advantageous by ca. 40–75 kJ·mol^–1^ when compared to its nitro equivalent in the crystal structure and by at least 20 kJ·mol^–1^ when compared to the *endo*-nitrito analogue. Such results justify the absence of the *exo*-nitrito species in the crystal structure under the examined conditions.

**Figure 11:**
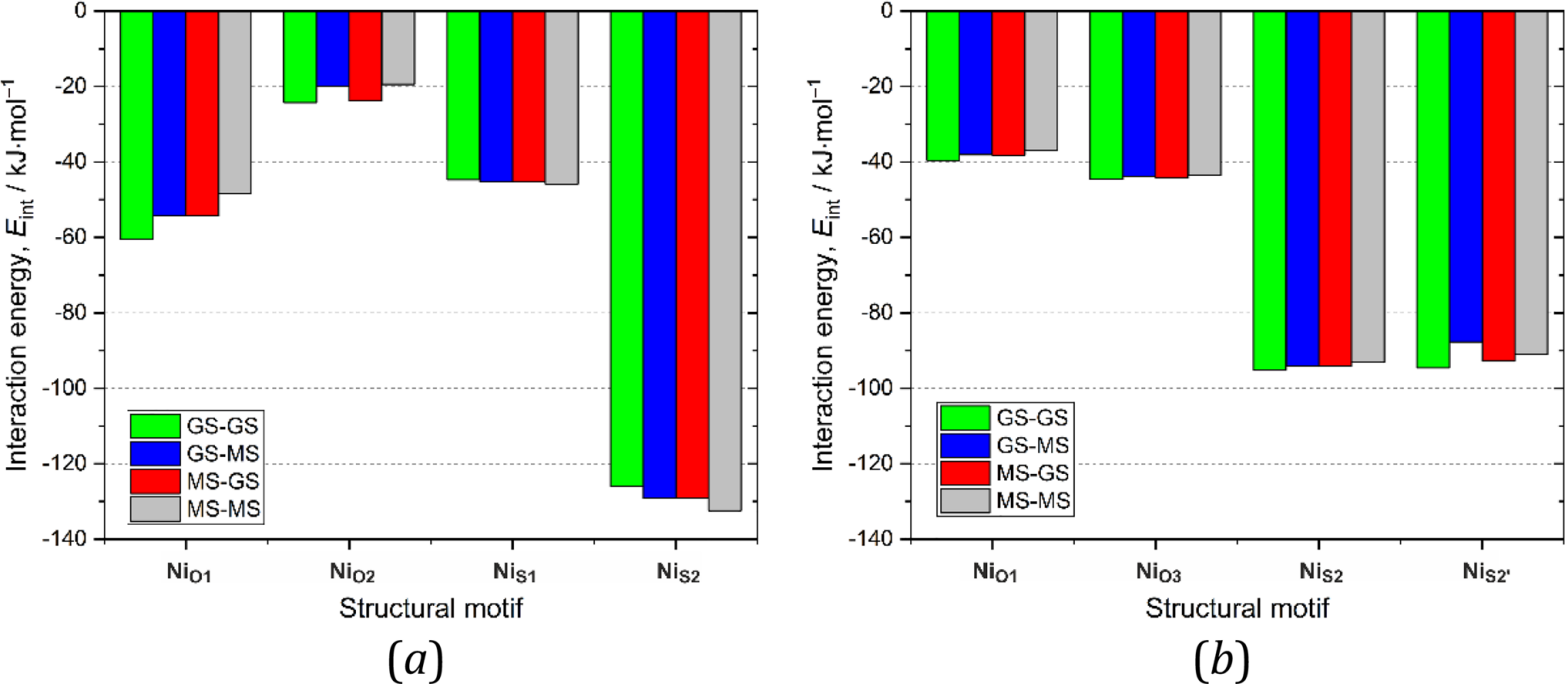
Motif interaction energies for selected (a) **Ni-2a** and (b) **Ni-2b** dimers containing one or two metastable (MS) *endo*-nitrito forms compared with ground-state (GS) dimers. For numerical data, see the Supporting Information. GS-MS means that the first molecule is in the ground state and the second molecule in the excited state; in the case of asymmetric motifs, the first molecule is always the left one, as shown in Figure 4.

Apart from the advantageous energies calculated for the respective molecules, intermolecular interactions formed by the GS and MS species are also very important. Those formed by GS species break during the isomerization process, whereas those formed by MS speciesstabilize the final product. Thus, various types of dimers consisting of the *endo*-nitrito metastable state or the nitro ground-state form were considered and subjected to interaction-energy calculations. Figure 11 shows the interaction energy trends when the nitro isomers are substituted with their *endo*-nitrito equivalents for the selected representative dimers. In the case of **Ni-2a**, it appears that dimers formed by the nitro and *endo*-nitrito isomers are characterized by rather comparable stabilization energy values. As shown in Figure 11a, it seems that the **Ni**_**O1**_ and **Ni**_**O2**_ motifs are best stabilized when both components exist in the nitro form (GS–GS-type dimers), whereas the least-stabilized motif is composed of two *endo*-nitrito isomers (MS–MS-type dimers). The differences between the extreme values do not exceed 20% of the GS–GS interaction energy. The opposite trend is present for the **Ni**_**S**_-type motifs dominated by the π···π stacking interactions. However, in this case the differences between the extreme interaction energy values are significantly smaller (reaching a few percent at maximum). These results show that the nitro isomer is indeed better stabilized on average in the **Ni-2a** crystal structure than the *endo*-nitrito form, but the difference is rather moderate. In turn, for **Ni-2b**, changing the nitro form to the its *endo*-nitrito linkage isomer weakens the dimer stabilization energy in all the cases, but the energy differences are less pronounced than those in the case of **Ni-2a**. Considering solely the molecular energies of various analyzed isomers and the strengths of the intermolecular interaction they form in the crystal structure, **Ni-2b** should be slightly more eager to undergo the isomerization reaction. On the other hand, the reaction cavity volume is significantly greater for **Ni-2a**, which is beneficial regarding potential chemical transformations.

## 4 Summary and Conclusions

In the current contribution, two promising photoswitchable nickel(II) nitro coordination compounds and their copper(II) analogues were synthesized and comprehensively investigated using (photo)crystallographic, spectroscopic, and computational techniques. In all these systems, the metal center is chelated by (*N*,*N*,*O*)-donor ligands containing either 2-picolylamine or 8-aminoquinoline fragments and coordinated by the nitro group as an ambidentate photoactive moiety. The obtained compounds are easy to synthesize and stable under ambient conditions. The main difference between the nickel complexes and the analogous copper systems is the preferred binding mode of the NO_2_ fragment. The nickel systems exist preferably in the nitro form, whereas the obtained copper compounds exist as the κ-nitrito linkage isomers. Otherwise, the crystal packing is, in general, similar in all the studied crystal structures. It seems that the nitro group has slightly stronger interactions with the surrounding species in the case of the **2a** complexes than in the case of **2b**, which possess larger (*N*,*N*,*O*)-donor ligands. In all the systems, the reaction cavity volumes are comparable to the literature-reported values for photoswitchable systems of these kinds.

Two facts are worth mentioning regarding the behavior of the samples regarding the temperature changes in the 100–300 K range. First, the monoclinic-to-triclinic transition of **Ni-2b** single crystals was observed at ca. 275 K. Second, the thermally governed partial transformation of the nitro isomer to its *endo*-nitrito equivalent takes place in **Ni-2a**. In the former case, the phase transition has only a minor influence on the crystal packing and almost does not affect the type and strength of the interatomic interactions. In the latter case, the *endo*-nitrito isomer appears in the crystal structure at temperatures between 100 and 180 K (upon both the cooling and the heating of the crystal); however, its population does not exceed 12%. Regarding the reaction cavity volume, its changes are caused mainly by the temperature factor and the effect of the increased population of the *endo*-nitrito linkage isomer can be neglected.

The solid-state IR spectroscopy results indicated that close to 100% LED-light-induced (530 nm) isomerization of the thin-film samples of nickel compounds was achieved at 10–140 K for **Ni-2a** and 140–180 K for **Ni-2b**. The metastable linkage isomer was present up to 220 K, which is still a relatively high temperature compared to the most effectively working nitro complexes of transition metals.^8,12,15,16,19−21,23,24,93,94^ The experiments also confirmed the full reversibility of the process. In turn, as far as the copper complexes are concerned, they are active only at very low temperatures and the best results are achieved at 10 K. The light-induced changes are rather moderate in this case, while some traces of the metastable forms last until 60–80 K at best. IR experiments suggest that the initial κ-nitrito isomer transforms partially to the nitro form. Such a switching could be expected, since the nitro binding mode is the second most common linkage isomer as far as the copper complexes are concerned. Regarding the thermally induced isomerization, it should be noted that for all the samples the temperature effect itself is rather small. The most visible changes were observed for **Ni-2a**, which is in agreement with the multi-temperature X-ray diffraction results.

The conducted photocrystallographic experiments confirmed the photoswitching properties of the studied nickel complexes. However, the achieved maximum light-induced metastable state populations in the single crystals reached about 35% in both cases. As indicated by the IR experiments on thin films, the achievable conversion could potentially be higher; however, longer irradiation caused the single crystal to decay. Another interesting observation, not reported before, is the fact that the cavity volume shrinks along with the photogeneration of the *endo*-nitrito isomer. Nevertheless, it should be noted that in all the other cases in which cavity volume changes due to crystal irradiation were reported, the *exo*-nitrito binding mode was formed prior to the *endo*-nitrito species. In both the **Ni-2a** and **Ni-2b** crystal structures, no traces of this former isomer were detected. This could be the explanation for the trend, as the *exo*-nitrito linkage isomer may cause more significant structural changes in its closest surroundings due to its shape. The reluctance of the examined systems to exist in the *exo*-nitrito form was supported by the computational analysis. Computations also showed that the *endo*-nitrito isomer is well-stabilized in the analyzed crystal structures.

## Supporting Information Available

Comprehensive synthesis information, compound characterization data, crystal packing figures, supporting spectroscopic plots, cavity volume data, and additional computational data (PDF)
GIFs showing the structures and vibrational modes of **Cu-2a** and **Na-2a** (ZIP)

## Accession Codes

CCDC 2110808–2110835 contain the supplementary crystallographic data for this paper. These data can be obtained free of charge via www.ccdc.cam.ac.uk/data_request/cif, or by emailing data_request@ccdc.cam.ac.uk, or by contacting The Cambridge Crystallographic Data Centre, 12 Union Road, Cambridge CB2 1EZ, UK; fax: +44 1223 336033.

## References

[ref1] Mustroph H.; Stollenwerk M.; Bressau V. Current developments in optical data storage with organic dyes. Angew. Chem., Int. Ed. 2006, 45, 2016–2035. 10.1002/anie.200502820. 16518782

[ref2] Kamtekar K. T.; Monkman A. P.; Bryce M. R. Recent advances in white organic light-emitting materials and devices (WOLEDs). Adv. Mater. 2010, 22, 572–582. 10.1002/adma.200902148. 20217752

[ref3] Holick M.; MacLaughlin J.; Clark M.; Holick S.; Potts J.; Anderson R.; Blank I.; Parrish J.; Elias P. Photosynthesis of previtamin D3 in human skin and the physiologic consequences. Science 1980, 210 (4466), 203–205. 10.1126/science.6251551. 6251551

[ref4] Yildiz I.; Deniz E.; Raymo F. M. Fluorescence modulation with photochromic switches in nanostructured constructs. Chem. Soc. Rev. 2009, 38, 1859–1867. 10.1039/b804151m. 19551167

[ref5] Dong H.; Zhu H.; Meng Q.; Gong X.; Hu W. Organic photoresponse materials and devicesw. Chem. Soc. Rev. 2012, 41, 1754–1808. 10.1039/C1CS15205J. 22158983

[ref6] Volarić J.; Szymanski W.; Simeth N. A.; Feringa B. L. Molecular photoswitches in aqueous environments. Chem. Soc. Rev. 2021, 50 (22), 12377–12449. 10.1039/D0CS00547A. 34590636 PMC8591629

[ref7] Goulet-Hanssens A.; Eisenreich F.; Hecht S. Enlightening Materials with Photoswitches. Adv. Mater. 2020, 32 (20), 1905966 10.1002/adma.201905966. 31975456

[ref8] Hatcher L. E.; Skelton J. M.; Warren M. R.; Raithby P. R. Photocrystallographic studies on transition metal nitrito metastable linkage isomers: manipulating the metastable state. Acc. Chem. Res. 2019, 52, 1079–1088. 10.1021/acs.accounts.9b00018. 30916544 PMC7005940

[ref9] Coppens P.; Fomitchev D. V.; Carducci M. D.; Culp K. Crystallography of molecular excited states. Transition-metal nitrosyl complexes and the study of transient species.. J. Chem. Soc., Dalton Trans. 1998, 865–872. 10.1039/a708604k.

[ref10] Stepanenko I.; Zalibera M.; Schaniel D.; Telser J.; Arion V. B. Ruthenium-nitrosyl complexes as NO-releasing molecules, potential anticancer drugs, and photoswitches based on linkage isomerism. Dalton Trans. 2022, 51, 5367–5393. 10.1039/D2DT00290F. 35293410

[ref11] Vittal J. J.; Quah H. S. Photochemical reactions of metal complexes in the solid state. Dalton Trans. 2017, 46 (22), 7120–7140. 10.1039/C7DT00873B. 28540960

[ref12] Hatcher L. E.; Raithby P. R. Solid-state photochemistry of molecular photo-switchable species: the role of photocrystallographic techniques. Acta Cryst. Sect. C 2013, 69, 1448–1456. 10.1107/S010827011303223X. 24311489

[ref13] Schaniel D.; Casaretto N.; Bendeif E.-E.; Woike T.; Gallien A. K. E.; Klüfers P.; Kutniewska S. E.; Kamiński R.; Bouchez G.; Boukheddaden K.; Pillet S. Evidence for a photoinduced isonitrosyl isomer in ruthenium dinitrosyl compounds. CrystEngComm 2019, 21, 5804–5810. 10.1039/C9CE01119F.

[ref14] Schaniel D.; Bendeif E.-E.; Woike T.; Böttcher H.-C.; Pillet S. Wavelength-selective photoisomerisation of nitric oxide and nitrite in a rhodium complex. CrystEngComm 2018, 20, 7100–7108. 10.1039/C8CE01345D.

[ref15] Kutniewska S. E.; Krówczyński A.; Kamiński R.; Jarzembska K. N.; Pillet S.; Wenger E.; Schaniel D. Photocrystallographic and spectroscopic studies of a model (N,N,O)-donor square-planar nickel(II) nitro complex: in search of high-conversion and stable photoswitchable materials. IUCrJ 2020, 7, 1188–1198. 10.1107/S205225252001307X. PMC7642791 33209329

[ref16] Kutniewska S. E.; Kamiński R.; Buchowicz W.; Jarzembska K. N. Photo- and thermoswitchable half-sandwich nickel(II) complex: [Ni(η^5^-C_5_H_5_)(IMes)(η^1^-NO_2_)]. Inorg. Chem. 2019, 58, 16712–16721. 10.1021/acs.inorgchem.9b02836. 31773953

[ref17] Nakamura I.; Sumitani R.; Mochida T. Nitro-nitrito Photoisomerization of cationic platinum(II) complexes in the solid state: reactivity in polymorphic crystals and glassy state. Cryst. Growth Des. 2021, 21 (3), 1861–1868. 10.1021/acs.cgd.1c00004.

[ref18] Nakamura I.; Funasako Y.; Mochida T. Nitro-nitrito photoisomerization of platinum(II) complexes with Pt(NO_2_)_4_^2–^ and (FSO_2_)_2_N^–^ anions: correlation between isomerization ratio and reaction cavity. Cryst. Growth Des. 2020, 20 (12), 8047–8052. 10.1021/acs.cgd.0c01294.

[ref19] Hatcher L. E.; Skelton J. M.; Warren M. R.; Stubbs C.; da Silva E. L.; Raithby P. R. Monitoring photo-induced population dynamics in metastable linkage isomer crystals: A crystallographic kinetic study of [Pd(Bu_4_dien)NO_2_]BPh_4_. Phys. Chem. Chem. Phys. 2018, 20, 5874–5886. 10.1039/C7CP05422J. 29417100

[ref20] Hatcher L. E.; Raithby P. R. The impact of hydrogen bonding on 100% photo-switching in solid-state nitro-nitrito linkage isomers. CrystEngComm 2017, 19, 6297–6304. 10.1039/C7CE01366C.

[ref21] Hatcher L. E. Raising the (metastable) bar: 100% photoswitching in [Pd(Bu_4_dien))η^1^-NO_2_)]^+^ approaches ambient temperature. CrystEngComm 2016, 18, 4180–4187. 10.1039/C5CE02434J.

[ref22] Hatcher L. E.; Bigos E. J.; Bryant M. J.; MacCready E. M.; Robinson T. P.; Saunders L. K.; Thomas L. H.; Beavers C. M.; Teat S. J.; Christensen J.; Raithby P. R. Thermal and photochemical control of nitro-nitrito linkage isomerism in single-crystals of [Ni(medpt)(NO_2_)(η^2^-ONO)]. CrystEngComm 2014, 16, 8263–8271. 10.1039/C4CE00675E.

[ref23] Warren M. R.; Brayshaw S. K.; Hatcher L. E.; Johnson A. L.; Schiffers S.; Warren A. J.; Teat S. J.; Warren J. E.; Woodall C. H.; Raithby P. R. Photoactivated linkage isomerism in single crystals of nickel, palladium and platinum di-nitro complexes - a photocrystallographic investigation. Dalton Trans. 2012, 41, 13173–13179. 10.1039/c2dt30314k. 22996434

[ref24] Hatcher L. E.; Warren M. R.; Allan D. R.; Brayshaw S. K.; Johnson A. L.; Fuertes S.; Schiffers S.; Stevenson A. J.; Teat S. J.; Woodall C. H.; Raithby P. R. Metastable linkage isomerism in [Ni(Et_4_dien)(NO_2_)_2_]: A combined thermal and photocrystallographic structural investigation of a nitro/nitrito interconversion. Angew. Chem., Int. Ed. 2011, 50, 8371–8374. 10.1002/anie.201102022. 21780258

[ref25] Hatcher L. E.; Christensen J.; Hamilton M. L.; Trincao J.; Allan D. R.; Warren M. R.; Clarke I. P.; Towrie M.; Fuertes S.; Wilson C. C.; Woodall C. H.; Raithby P. R. Steady-state and pseudo-steady-state photocrystallographic studies on linkage isomers of [Ni(Et_4_dien)(η^2^-O,ON)(η^1^-NO_2_)]: Identification of a new linkage isomer. Chem. Eur. J. 2014, 20, 3128–3134. 10.1002/chem.201304172. 24519880

[ref26] Kovalevsky A. Y.; Bagley K. A.; Cole J. M.; Coppens P. Light-induced metastable linkage isomers of ruthenium sulfur dioxide complexes. Inorg. Chem. 2003, 42, 140–147. 10.1021/ic025997g. 12513088

[ref27] Kovalevsky A. Y.; Bagley K. A.; Coppens P. The first photocrystallographic evidence for light-induced metastable linkage isomers of ruthenium sulfur dioxide complexes. J. Am. Chem. Soc. 2002, 124, 9241–9248. 10.1021/ja026045c. 12149030

[ref28] Cole J. M.; Velazquez-Garcia J. d. J.; Gosztola D. J.; Wang S. G.; Chen Y.-S. η^2^-SO_2_ Linkage photoisomer of an osmium coordination complex. Inorg. Chem. 2018, 57, 2673–2677. 10.1021/acs.inorgchem.7b03032. 29461811

[ref29] Sylvester S. O.; Cole J. M.; Waddell P. G.; Nowell H.; Wilson C. SO_2_ phototriggered crystalline nanomechanical transduction of aromatic rotors in tosylates: rationalization via photocrystallography of [Ru(NH_3_)_4_SO_2_X]tosylate_2_ (X = pyridine, 3-Cl-pyridine, 4-Cl-pyridine). J. Phys. Chem. C 2014, 118, 16003–16010. 10.1021/jp503711h.

[ref30] Snow M. R.; Boomsma R. F. The crystal structures and isomerization of the linkage isomers thiocyanato- and isothiocyanato-pentaamminecobalt(III) dichloride, [Co(SCN)(NH_3_)_5_]Cl_2_.H_2_O, and [Co(NCS)(NH_3_)_5_]Cl_2_. Acta Cryst. Sect. B 1972, 28 (6), 1908–1913. 10.1107/S0567740872005217.

[ref31] Schaniel D.; Woike T.; Delley B.; Boskovic C.; Gudel H. U. Photogeneration of metastable side-on N2 linkage isomers in [Ru(NH3)5N2]Cl2, [Ru(NH3)5N2]Br2 and [Os(NH3)5N2]Cl2. Phys. Chem. Chem. Phys. 2008, 10, 5531–5538. 10.1039/b806933f. 18956087

[ref32] Boldyreva E. V. Crystal-Structure Aspects of Solid-State Inner-Sphere Isomerization in Nitro(nitrito)pentaamminecobalt(III) Complexes. Russ. J. Coord. Chem. 2001, 27 (407), 297–323. 10.1023/A:1011392613014.

[ref33] Kubota M.; Ohba S. Nitro-nitrito linkage photoisomerization in crystals of pentaamminenitrocobalt(III) dichloride. Acta Cryst. Sect. B 1992, 48, 627–632. 10.1107/S010876819200404X.

[ref34] Ahmed E.; Chizhik S.; Sidelnikov A.; Boldyreva E.; Naumov P. Relating Excited States to the Dynamics of Macroscopic Strain in Photoresponsive Crystals. Inorg. Chem. 2022, 61 (8), 3573–3585. 10.1021/acs.inorgchem.1c03607. 35170305

[ref35] Novozhilova I.; Coppens P.; Lee J.; Richter-Addo G. B.; Bagley K. A. Experimental and density functional theoretical investigations of linkage isomerism in six-coordinate {FeNO}6Iron porphyrins with axial nitrosyl and nitro ligands. J. Am. Chem. Soc. 2006, 128 (6), 2093–2104. 10.1021/ja0567891. 16464112

[ref36] Casaretto N.; Pillet S.; Bendeif E.-E.; Schaniel D.; Gallien A. K. E.; Klüfers P.; Woike T. Photocrystallography and IR spectroscopy of light-induced linkage NO isomers in [RuBr(NO)_2_(PCyp_3_)2]BF_4_. Acta Cryst. B 2015, 71, 788–797. 10.1107/S2052520615018132. 26634736

[ref37] Fomitchev D. V.; Novozhilova I.; Coppens P. Photo-induced linkage isomerism of transition metal nitrosyl and dinitrogen complexes studied by photocrystallographic techniques. Tetrahedron 2000, 56, 6813–6820. 10.1016/S0040-4020(00)00503-2.

[ref38] Coppens P. The dramatic development of X-ray photocrystallography over the past six decades. Struct. Dyn. 2017, 4, 032102 10.1063/1.4975301. 28191481 PMC5291789

[ref39] Cole J. M. Photocrystallography. Acta Cryst. 2008, A64, 259–271. 10.1107/S0108767307065324. 18156690

[ref40] Coppens P. The new photocrystallography. Angew. Chem., Int. Ed. 2009, 48, 4280–4281. 10.1002/anie.200900910. 19378302

[ref41] Woollard-Shore J. G.; Holland J. P.; Jones M. W.; Dilworth J. R. Nitrite reduction by copper complexes. Dalton Trans. 2010, 39, 1576–1585. 10.1039/B913463H. 20104320

[ref42] Pajunen A.; Pajunen S. Crystal structure of diethylenetriamine-N,N’,N″-nitrito-O,O’-nitrocopper(II), C_4_H_13_CuN_5_O_4_.. Z. Kristallogr. 1998, 213, 619–620. 10.1524/ncrs.1998.213.14.619.

[ref43] Dabrowski J.; Krówczyński A. Ni(II) complexes with bis(β-acylvinyl)amines and (8-quinolyl-β-acylvinyl)amines. Z. Naturforsch. 1977, 32b, 62–67. 10.1515/znb-1977-0115.

[ref44] Kamiński R.; Jarzembska K. N.; Kutyła S. E.; Kamiński M. A portable light-delivery device for in situ photocrystallographic experiments at home laboratory. J. Appl. Crystallogr. 2016, 49, 1383–1387. 10.1107/S1600576716008128.

[ref45] Sheldrick G. M. SHELXT - integrated space-group and crystal-structure determination. Acta Crystallogr., Sect. A 2015, 71, 3–8. 10.1107/S2053273314026370. PMC4283466 25537383

[ref46] Petříček V.; Dušek M.; Palatinus L. Crystallographic computing system JANA2006: general features. Z. Kristallogr. 2014, 229, 345–352. 10.1515/zkri-2014-1737.

[ref47] Fournier B.; Coppens P. On the assessment of time-resolved diffraction results. Acta Crystallogr., Sect. A 2014, 70, 291–299. 10.1107/S2053273314006305. PMC4011010 24815977

[ref48] Schmøkel M. S.; Kamiński R.; Benedict J. B.; Coppens P. Data scaling and temperature calibration in time-resolved photocrystallographic experiments. Acta Crystallogr., Sect. A 2010, 66, 632–636. 10.1107/S0108767310029429. 20962370

[ref49] Allen F. H. The Cambridge Structural Database: a quarter of a million crystal structures and rising. Acta Cryst. Sect. B 2002, 58, 380–388. 10.1107/S0108768102003890. 12037359

[ref50] Groom C. R.; Bruno I. J.; Lightfoot M. P.; Ward S. C. The Cambridge Structural Database. Acta Cryst. Sect. B 2016, 72, 171–179. 10.1107/S2052520616003954. PMC4822653 27048719

[ref51] Le Bail A.; Duroy H.; Fourquet J. L. Ab-initio structure determination of LiSbWO_6_ by X-ray powder diffraction. Mater. Res. Bull. 1988, 23, 447–452. 10.1016/0025-5408(88)90019-0.

[ref52] Dusek M.; Petricek V.; Wunschel M.; Dinnebier R. E.; van Smaalen S. Refinement of modulated structures against X-ray powder diffraction data with JANA2000. J. Appl. Crystallogr. 2001, 34 (3), 398–404. 10.1107/S0021889801003302.

[ref53] Frisch M. J.; Trucks G. W.; Schlegel H. B.; Scuseria G. E.; Robb M. A.; Cheeseman J. R.; Scalmani G.; Barone V.; Petersson G. A.; Nakatsuji H.; Li X.; Caricato M.; Marenich A. V.; Bloino J.; Janesko B. G.; Gomperts R.; Mennucci B.; Hratchian H. P.; Ortiz J. V.; Izmaylov A. F.; Sonnenberg J. L.; Williams; Ding F.; Lipparini F.; Egidi F.; Goings J.; Peng B.; Petrone A.; Henderson T.; Ranasinghe D.; Zakrzewski V. G.; Gao J.; Rega N.; Zheng G.; Liang W.; Hada M.; Ehara M.; Toyota K.; Fukuda R.; Hasegawa J.; Ishida M.; Nakajima T.; Honda Y.; Kitao O.; Nakai H.; Vreven T.; Throssell K.; Montgomery J. A. Jr.; Peralta J. E.; Ogliaro F.; Bearpark M. J.; Heyd J. J.; Brothers E. N.; Kudin K. N.; Staroverov V. N.; Keith T. A.; Kobayashi R.; Normand J.; Raghavachari K.; Rendell A. P.; Burant J. C.; Iyengar S. S.; Tomasi J.; Cossi M.; Millam J. M.; Klene M.; Adamo C.; Cammi R.; Ochterski J. W.; Martin R. L.; Morokuma K.; Farkas O.; Foresman J. B.; Fox D. J. Gaussian 16; Gaussian, Inc.: Wallingford, CT, 2016.

[ref54] Kamiński R.; Schmøkel M. S.; Coppens P. Constrained excited-state structure in molecular crystals by means of the QM/MM approach: toward the prediction of photocrystallographic results. J. Phys. Chem. Lett. 2010, 1, 2349–2353. 10.1021/jz100809q.

[ref55] Allen F. H.; Kennard O.; Watson D. G.; Brammer L.; Orpen A. G.; Taylor R. Tables of bond lengths determined by X-ray and neutron diffraction. Part 1. Bond lengths in organic compounds. J. Chem. Soc., Perkin Trans. 2 1987, S1–S19. 10.1039/p298700000s1.

[ref56] Allen F. H.; Bruno I. J. Bond lengths in organic and metal-organic compounds revisited: X-H bond lengths from neutron diffraction data. Acta Cryst. Sect. B 2010, 66, 380–386. 10.1107/S0108768110012048. 20484809

[ref57] Becke A. D. Density-functional exchange-energy approximation with correct asymptotic behavior. Phys. Rev. A 1988, 38, 3098–3100. 10.1103/PhysRevA.38.3098. 9900728

[ref58] Perdew J. P. Density-functional approximation for the correlation energy of the inhomogeneous electron gas. Phys. Rev. B 1986, 33, 8822–8824. 10.1103/PhysRevB.33.8822. 9938299

[ref59] Lee C.; Yang W.; Parr R. G. Development of the Colle-Salvetti correlation-energy formula into a functional of the electron density. Phys. Rev. B 1988, 37, 785–789. 10.1103/PhysRevB.37.785. 9944570

[ref60] Krishnan R.; Binkley J. S.; Seeger R.; Pople J. A. Self-consistent molecular orbital methods. XX. A basis set for correlated wave functions. J. Chem. Phys. 1980, 72, 650–654. 10.1063/1.438955.

[ref61] Clark T.; Chandrasekhar J.; Spitznagel G. W.; Schleyer P. v. R. Efficient diffuse function-augmented basis sets for anion calculations. III. The 3-21+G basis set for first-row elements, Li-F. J. Comput. Chem. 1983, 4, 294–301. 10.1002/jcc.540040303.

[ref62] McLean A. D.; Chandler G. S. Contracted Gaussian basis sets for molecular calculations. I. Second row atoms, Z = 11–18. J. Chem. Phys. 1980, 72, 5639–5648. 10.1063/1.438980.

[ref63] Rappé A. K.; Casewit C. J.; Colwell K. S.; Goddard W. A. III; Skiff W. M. UFF, a full periodic-table force-field for molecular mechanics and molecular-dynamics simulations. J. Am. Chem. Soc. 1992, 114, 10024–10035. 10.1021/ja00051a040.

[ref64] Hirshfeld F. L. Bonded-atom fragments for describing molecular charge densities. Theor. Chim. Acta 1977, 44, 129 10.1007/BF00549096.

[ref65] Grimme S. Accurate description of van der Waals complexes by density functional theory including empirical corrections. J. Comput. Chem. 2004, 25, 1463–1473. 10.1002/jcc.20078. 15224390

[ref66] Grimme S. Semiempirical GGA-type density functional constructed with a long-range dispersion correction. J. Comput. Chem. 2006, 27, 1787–1799. 10.1002/jcc.20495. 16955487

[ref67] Grimme S.; Antony J.; Ehrlich S.; Krieg H. A consistent and accurate ab initio parameterization of density functional dispersion correction (DFT-D) for the 94 elements H-Pu. J. Chem. Phys. 2010, 132, 154104 10.1063/1.3382344. 20423165

[ref68] Grimme S.; Ehrlich S.; Goerigk L. Effect of the damping function in dispersion corrected density functional theory. J. Comput. Chem. 2011, 32, 1456–1465. 10.1002/jcc.21759. 21370243

[ref69] Boys S. F.; Bernardi F. The calculation of small molecular interactions by the differences of separate total energies. Some procedures with reduced errors. Mol. Phys. 1970, 19, 553–566. 10.1080/00268977000101561.

[ref70] Simon S.; Duran M.; Dannenberg J. J. How does basis set superposition error change the potential surfaces for hydrogen bonded dimers?. J. Chem. Phys. 1996, 105, 11024–11031. 10.1063/1.472902.

[ref71] Kamiński R.; Jarzembska K. N.; Domagała S. CLUSTERGEN: a program for molecular cluster generation from crystallographic data. J. Appl. Crystallogr. 2013, 46, 540–534. 10.1107/S0021889813002173.

[ref72] Roger I.; Wilson C.; Senn H. M.; Sproules S.; Symes M. D. An investigation into the unusual linkage isomerization and nitrite reduction activity of a novel tris(2-pyridyl) copper complex. R. Soc. Open Sci. 2017, 4 (8), 170593 10.1098/rsos.170593. 28879000 PMC5579116

[ref73] Chao M. S.; Lu H. H.; Tsai M. L.; Lin C. M.; Wu M. P. Thermochromic nitro–nitrito interconversion mediated by weak-linked amide in nickel (II) diaminodiamide complexes in the solid state. Inorg. Chem. Commun. 2012, 24, 254–258. 10.1016/j.inoche.2012.07.001.

[ref74] Macrae C. F.; Bruno I. J.; Chisholm J. A.; Edgington P. R.; McCabe P.; Pidcock E.; Rodriguez-Monge L.; Taylor R.; van de Streek J.; Wood P. A. *Mercury CSD 2.0* - new features for the visualization and investigation of crystal structures. J. Appl. Crystallogr. 2008, 41, 466–470. 10.1107/S0021889807067908.

[ref75] Spackman M. A.; Jayatilaka D. Hirshfeld surface analysis. CrystEngComm 2009, 11, 19–32. 10.1039/B818330A.

[ref76] Turner M. J.; McKinnon J. J.; Wolff S. K.; Grimwood D. J.; Spackman P. R.; Jayatilaka D.; Spackman M. A. CRYSTALEXPLORER17; University of Western Australia: Crawley, Australia, 2017.

[ref77] Bajwa S. E.; Storr T. E.; Hatcher L.; Williams T. J.; Baumann C. G.; Whitwood A. C.; Allan D.; Teat S. J.; Raithby P. R.; Fairlamb I. J. S. On the appearance of nitrite anion in [PdX(OAc)L_2_] and [Pd(X)(C^∧^N)L] syntheses (X = OAc or NO_2_): photocrystallographic identification of metastable Pd(η^1^-ONO)(C^∧^N)PPh_3_. Chem. Sci. 2012, 3 (5), 1656–1661. 10.1039/c2sc01050j.

[ref78] Goodgame D. M. L.; Venanzi L. M. Diamine complexes of nickel(II). Part I. Complexes with N,N-diethylethylenediamine. J. Chem. Soc. 1963, 616–627. 10.1039/jr9630000616.

[ref79] Chattopadhyay T.; Ghosh M.; Majee A.; Nethaji M.; Das D. Linkage isomerism in 4-(2-aminoethyl)morpholine (L) complexes of nickel (II) nitrite: X-ray single crystal structure of trans-[NiL_2_(NO_2_)_2_]. Polyhedron 2005, 24, 1677–1681. 10.1016/j.poly.2005.04.039.

[ref80] Das D.; Laskar I. R.; Ghosh A.; Mondal A.; Okamoto K.-i.; Chaudhuri N. R. First structural characterisation of nitro-nitrito linkage isomers of nickel(II): Synthesis and single crystal structures of [NiL_2_(NO2)2] and [NiL2(ONO)2] [L = 1-(2-aminoethyl)piperidine]. J. Chem. Soc., Dalton Trans. 1998, 3987–3990. 10.1039/a806858e.

[ref81] Laskar I. R.; Ghosh A.; Mostafa G.; Das D.; Mondal A.; Chaudhuri N. R. Cis–trans isomerism in nickel(II)–diamine nitrite: Synthesis and single crystal structure of an unusual cis-dinitronickel(II) complex, [NiL_2_(NO_2_)_2_] (L = 1,2-diamino-2-methylpropane). Polyhedron 2000, 19, 1015–1020. 10.1016/S0277-5387(00)00365-X.

[ref82] Ribas J.; Diaz C.; Monfort M.; Vilana J. Polynuclear nickel(II) complexes with nitrito bridge and 1,3-diaminopropane as ligand. Inorg. Chim. Acta 1984, 90, L23–L25. 10.1016/S0020-1693(00)88033-0.

[ref83] Komeda N.; Nagao H.; Kushi Y.; Adachi G.-y.; Suzuki M.; Uehara A.; Tanaka K. Molecular Structure of Nitro- and Nitrito-Copper Complexes as Reaction Intermediates in Electrochemical Reduction of Nitrite to Dinitrogen Oxide. Bull. Chem. Soc. Jpn. 1995, 68 (2), 581–589. 10.1246/bcsj.68.581.

[ref84] Kujime M.; Izumi C.; Tomura M.; Hada M.; Fujii H. Effect of a Tridentate Ligand on the Structure, Electronic Structure, and Reactivity of the Copper(I) Nitrite Complex: Role of the Conserved Three-Histidine Ligand Environment of the Type-2 Copper Site in Copper-Containing Nitrite Reductases. J. Am. Chem. Soc. 2008, 130 (19), 6088–6098. 10.1021/ja075575b. 18412340

[ref85] Warren M. R.; Brayshaw S. K.; Johnson A. L.; Schiffers S.; Raithby P. R.; Easun T. L.; George M. W.; Warren J. E.; Teat S. J. Reversible 100% linkage isomerization in a single-crystal to single-crystal transformation: photocrystallographic identification of the metastable [Ni(dppe)(η^1^-ONO)Cl] isomer. Angew. Chem., Int. Ed. 2009, 48, 5711–5714. 10.1002/anie.200901706. 19565589

[ref86] Schaniel D.; Mockus N.; Woike T.; Klein A.; Sheptyakov D.; Todorova T.; Delley B. Reversible photoswitching between nitrito-N and nitrito-O isomers in trans-[Ru(py)_4_(NO_2_)_2_]. Phys. Chem. Chem. Phys. 2010, 12, 6171–6178. 10.1039/b921723a. 20390154

[ref87] Ooyama D.; Nagao N.; Nagao H.; Miura Y.; Hasegawa A.; Ando K.-i.; Howell F. S.; Mukaida M.; Tanaka K. Redox- and Thermally-Induced Nitro-Nitrito Linkage Isomerizations of Ruthenium(II) Complexes Having Nitrosyl as a Spectator Ligand. Inorg. Chem. 1995, 34 (24), 6024–6033. 10.1021/ic00128a013.

[ref88] Jarzembska K. N.; Hapka M.; Kamiński R.; Bury W.; Kutniewska S. E.; Szarejko D.; Szczęśniak M. M. On the nature of luminescence thermochromism of multinuclear copper(I) benzoate complexes in the crystalline state. Crystals 2019, 9, 36 10.3390/cryst9010036.

[ref89] Jarzembska K. N.; Kamiński R.; Fournier B.; Trzop E.; Sokolow J. D.; Henning R.; Chen Y.; Coppens P. Shedding light on the photochemistry of coinage-metal phosphorescent materials: a time-resolved Laue diffraction study of an Ag^I^-Cu^I^ tetranuclear complex. Inorg. Chem. 2014, 53, 10594–10601. 10.1021/ic501696y. 25238405 PMC4315237

[ref90] Makal A.; Benedict J.; Trzop E.; Sokolow J.; Fournier B.; Chen Y.; Kalinowski J. A.; Graber T.; Henning R.; Coppens P. Restricted photochemistry in the molecular solid state: structural changes on photoexcitation of Cu(I) phenanthroline metal-to-ligand charge transfer (MLCT) complexes by time-resolved diffraction. J. Phys. Chem. A 2012, 116, 3359–3365. 10.1021/jp300313s. 22385365 PMC3545449

[ref91] Makal A.; Trzop E.; Sokolow J.; Kalinowski J.; Benedict J.; Coppens P. The development of Laue techniques for single-pulse diffraction of chemical complexes: time-resolved Laue diffraction on a binuclear rhodium metal-organic complex. Acta Crystallogr., Sect. A 2011, 67, 319–326. 10.1107/S0108767311011883. 21694470 PMC3121236

[ref92] Benedict J. B.; Makal A.; Sokolow J. D.; Trzop E.; Scheins S.; Henning R.; Graber T.; Coppens P. Time-resolved Laue diffraction of excited species at atomic resolution: 100 ps single-pulse diffraction of the excited state of the organometallic complex Rh_2_(μ-PNP)_2_(PNP)_2_•BPh_4_. Chem. Commun. 2011, 47, 1704–1706. 10.1039/c0cc04997b. PMC3129623 21210070

[ref93] Skelton J. M.; Crespo-Otero R.; Hatcher L. E.; Parker S. C.; Raithby P. R.; Walsh A. Energetics, thermal isomerisation and photochemistry of the linkage-isomer system [Ni(Et_4_dien)(η^2^-O,ON)(η^1^-NO_2_)]. CrystEngComm 2015, 17, 383–394. 10.1039/C4CE01411A.

[ref94] Warren M. R.; Easun T. L.; Brayshaw S. K.; Deeth R. J.; George M. W.; Johnson A. L.; Schiffers S.; Teat S. J.; Warren A. J.; Warren J. E.; Wilson C. C.; Woodall C. H.; Raithby P. R. Solid-state interconversions: Unique 100% reversible transformations between the ground and metastable states in single-crystals of a series of nickel(II) nitro complexes. Chem. Eur. J. 2014, 20, 5468–5477. 10.1002/chem.201302053. 24644042 PMC4164279

