# Supplementary material for: Exploring Photoswitchable Properties of Two Nitro Nickel(II) Complexes with (N,N,O)-Donor Ligands and Their Copper(II) Analogues
Source: Inorg Chem. 2022 Apr 16;61(17):6624–40. doi: 10.1021/acs.inorgchem.2c00526 (PMC9066408; doi:10.1021/acs.inorgchem.2c00526)
Supplement: Supplementary file 1 — ic2c00526_si_001.pdf [file ic2c00526_si_001.pdf]

SUPPORTING INFORMATION

Exploring photoswitchable properties of two nitro nickel(II) complexes with (N,N,O)-donor ligands and their copper(II) analogues

Patryk Borowski,[a,§] Sylwia E. Kutniewska,[a,§] Radosław Kamiński,[a]
Adam Krówczyński,[a] Dominik Schaniel,[b] Katarzyna N. Jarzembska [a]*

[a] Department of Chemistry, University of Warsaw, Żwirki i Wigury 101, 02-089 Warsaw, Poland

[b] Université de Lorraine, CNRS, CRM², 54000 Nancy, France

§ Both authors contributed equally

* Corresponding author: Katarzyna N. Jarzembska (katarzyna.jarzembska@uw.edu.pl)

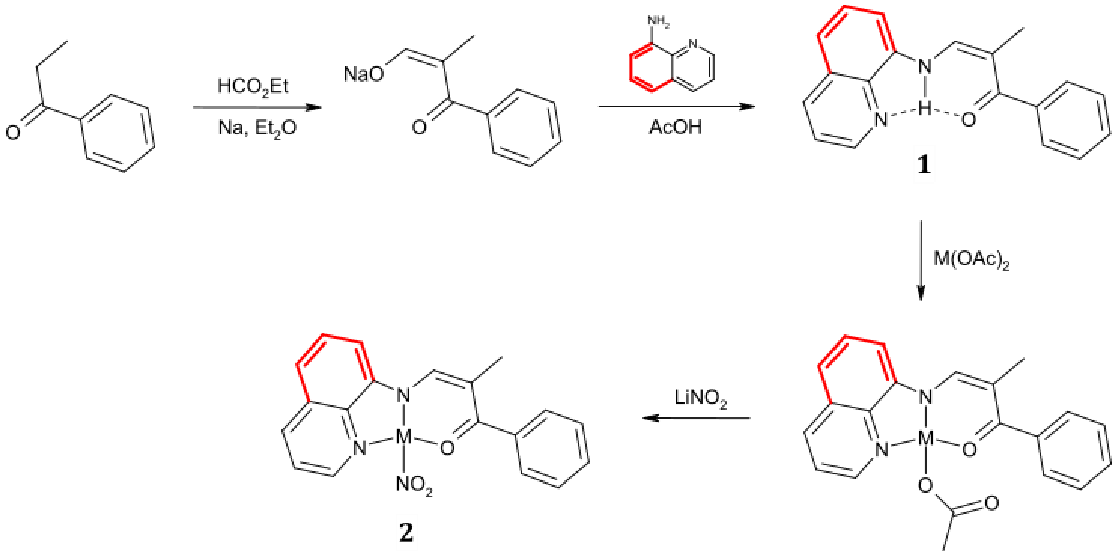

Scheme 1S. Schematic representation of the synthetic protocol applied to obtain the studied compounds (M = Ni or Cu). Bold red-color bonds denote that either 2-picolylamine (black only) or 8-aminoquinoline (black & red together) were used in the synthesis of the **1a** or **1b** ligands (and consequently – **M-2a** or **M-2b** complexes), respectively.

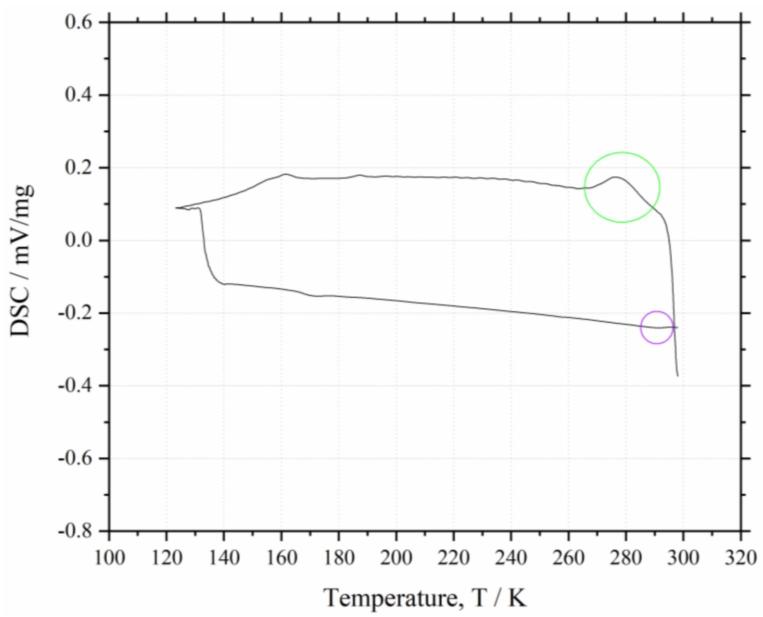

Figure 1S. DSC curves obtained for **Ni-2b** when scanning the 120–300 K temperature range. Green area shows the monoclinic-to-triclinic phase transition upon temperature decrease, while the purple circle indicates the reverse process.

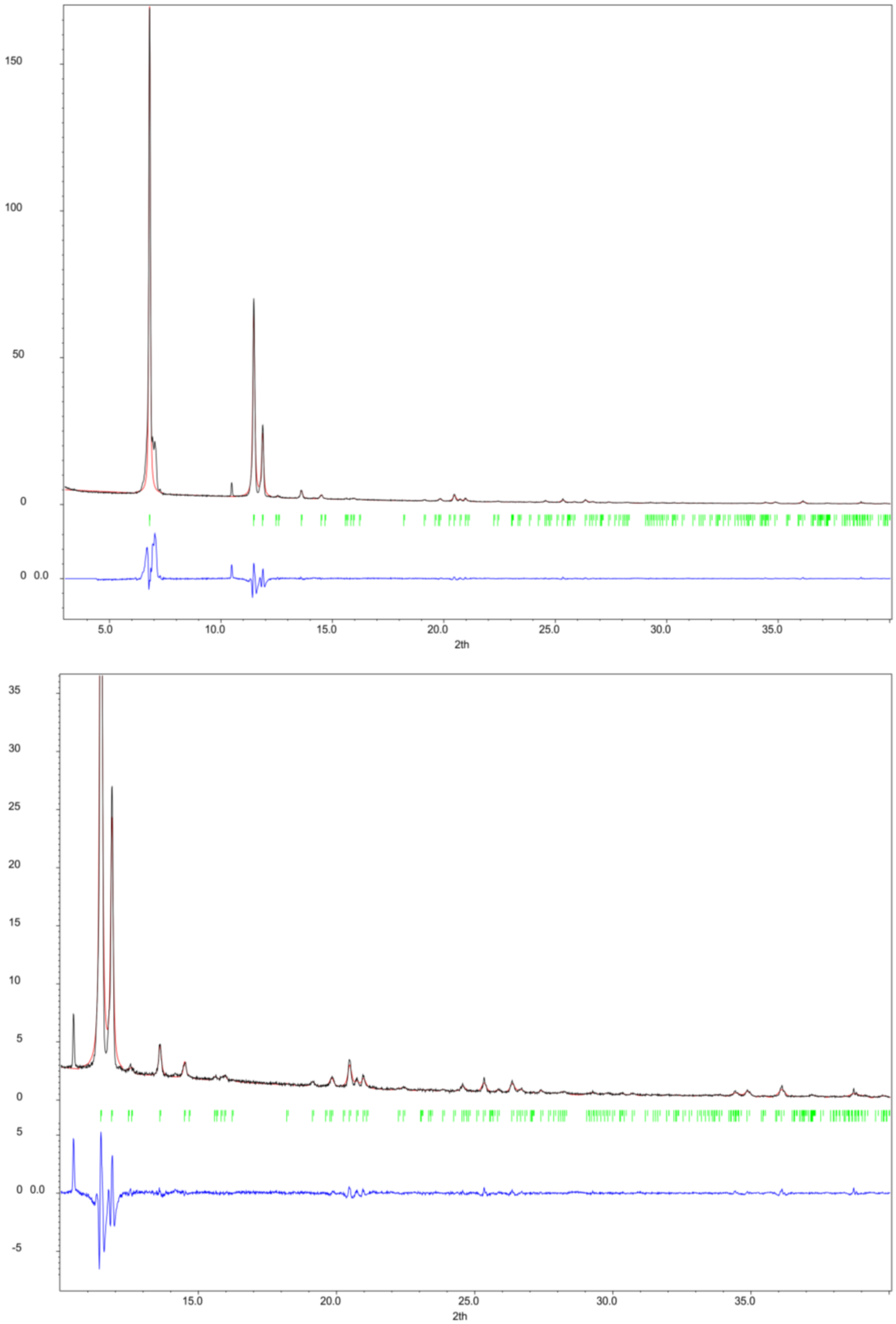

Figure 2S. PXRD patterns obtained for the **Ni-2a** bulk sample (top panel – full pattern, bottom – selected range magnification). Black curve – experimental pattern, red – Le Bail-fitted model, green bars – reflection positions (taking into account both $K_{\alpha1}$-$K_{\alpha2}$ splitting), blue – residual.

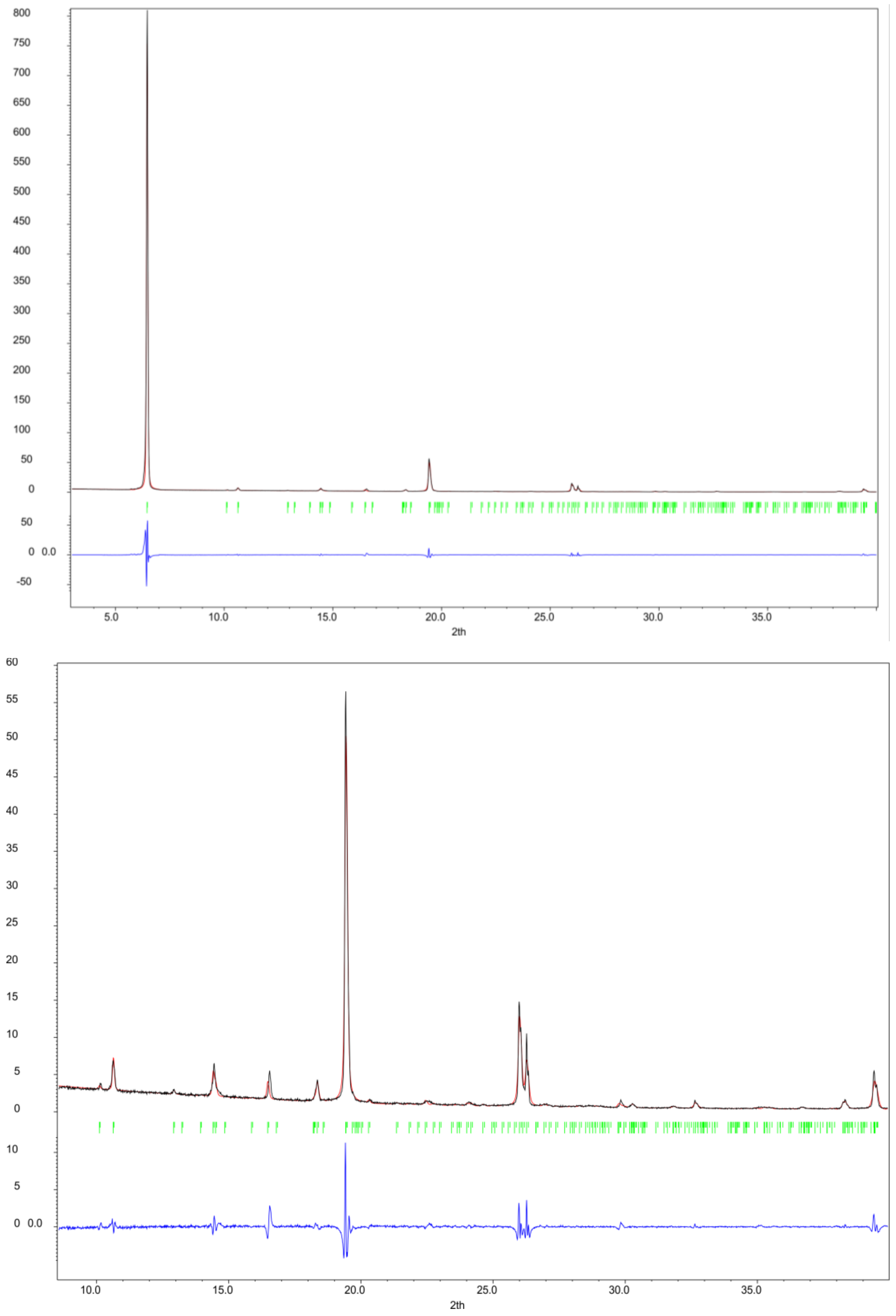

Figure 3S. PXRD patterns obtained for the **Ni-2b** bulk sample (top panel – full pattern, bottom – selected range magnification). Black curve – experimental pattern, red – Le Bail-fitted model, green bars – reflection positions (taking into account both $K_{\alpha1}$-$K_{\alpha2}$ splitting), blue – residual.

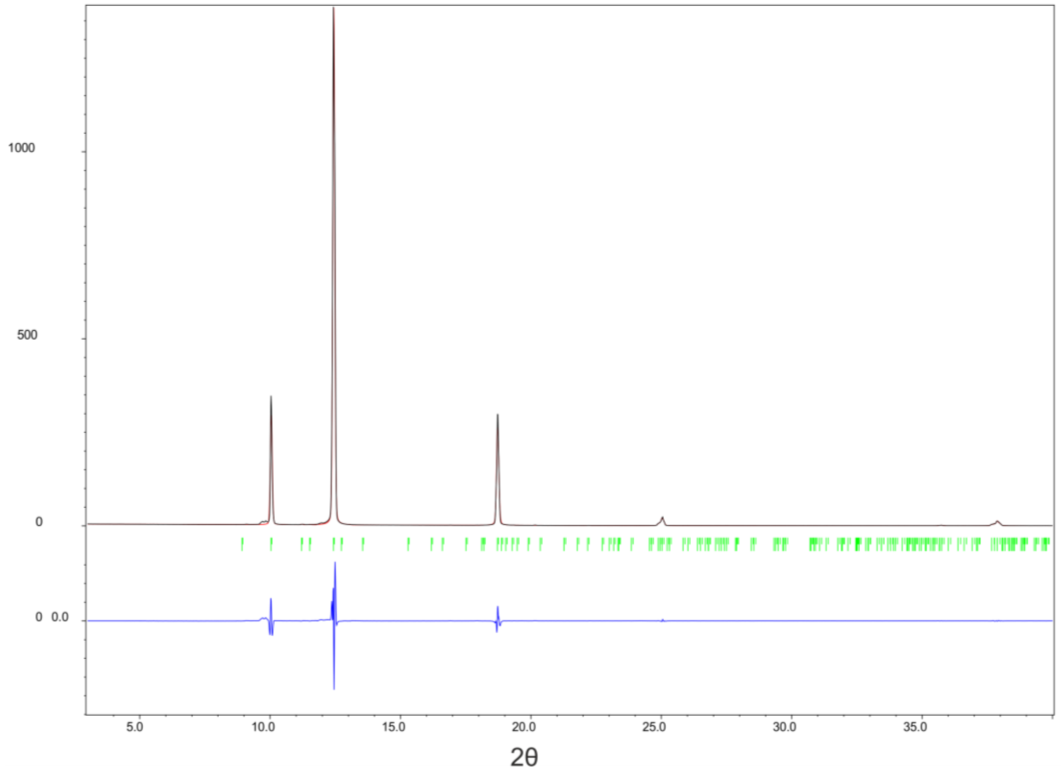

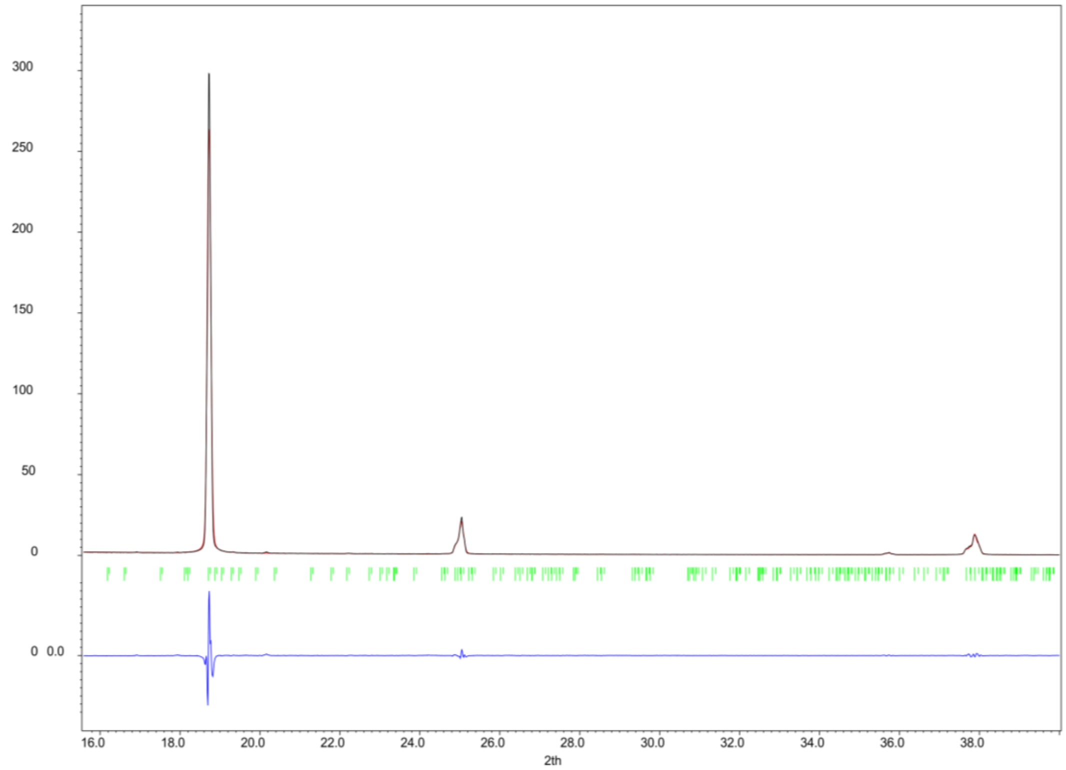

Figure 4S. PXRD patterns obtained for the **Cu-2a** bulk sample (top panel – full pattern, bottom – selected range magnification). Black curve – experimental pattern, red – Le Bail-fitted model, green bars – reflection positions (taking into account both $K_{\alpha 1}$-$K_{\alpha 2}$ splitting), blue – residual.

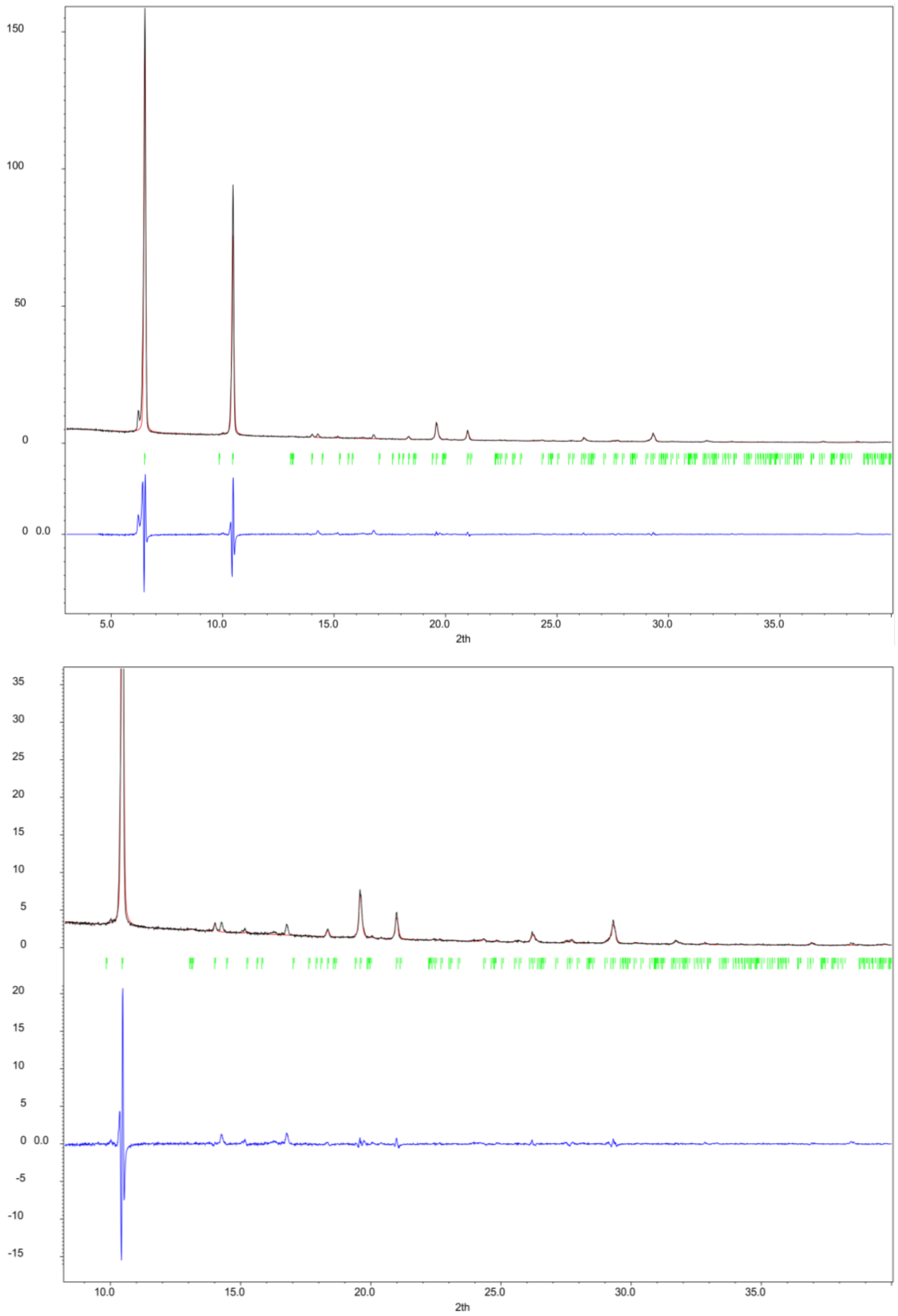

Figure 5S. PXRD patterns obtained for the **Cu-2b** bulk sample (top panel – full pattern, bottom – selected range magnification). Black curve – experimental pattern, red – Le Bail-fitted model, green bars – reflection positions (taking into account both $K_{\alpha 1}$-$K_{\alpha 2}$ splitting), blue – residual.

Table 1S. Summary of all collected X-ray diffraction datasets.

Complex	*Crystal No.*	*Temperature, T / K*	*Data collection code*	*Remarks*
**Ni-2a**	1st	290	**Ni-2a-290K-dark-cooling-xtal-1**	cooling cycle
		240	**Ni-2a-240K-dark-cooling-xtal-1**	cooling cycle
		200	**Ni-2a-200K-dark-cooling-xtal-1**	cooling cycle
		160	**Ni-2a-160K-dark-cooling-xtal-1**	cooling cycle
		140	**Ni-2a-140K-dark-cooling-xtal-1**	cooling cycle
		120	**Ni-2a-120K-dark-cooling-xtal-1**	cooling cycle
		100	**Ni-2a-100K-dark-cooling-xtal-1**	cooling cycle
		120	**Ni-2a-120K-dark-heating-xtal-1**	heating cycle
		140	**Ni-2a-140K-dark-heating-xtal-1**	heating cycle
		160	**Ni-2a-160K-dark-heating-xtal-1**	heating cycle
		180	**Ni-2a-180K-dark-heating-xtal-1**	heating cycle
		200	**Ni-2a-200K-dark-heating-xtal-1**	heating cycle
	2nd	140	**Ni-2a-140K-dark-xtal-2**	light-OFF data collection
		140	**Ni-2a-140K-irr-140K-xtal-2**	after irradiation at 140 K
		160	**Ni-2a-160K-irr-140K-xtal-2**	after irradiation at 140 K
		180	**Ni-2a-180K-irr-140K-xtal-2**	after irradiation at 140 K
		200	**Ni-2a-200K-irr-140K-xtal-2**	after irradiation at 140 K
**Ni-2b**	1st	290	**Ni-2b-290K-dark-cooling-xtal-1**	cooling cycle
		240	**Ni-2b-240K-dark-cooling-xtal-1**	cooling cycle
		200	**Ni-2b-200K-dark-cooling-xtal-1**	cooling cycle
		180	**Ni-2b-180K-dark-cooling-xtal-1**	cooling cycle
		160	**Ni-2b-160K-dark-cooling-xtal-1**	cooling cycle
	2nd	room temp.	**Ni-2b-RT-dark-xtal-2**	light-OFF data collection
		160	**Ni-2b-160K-dark-xtal-2**	light-OFF data collection
		160	**Ni-2b-160K-irr-160K-xtal-2**	after irradiation at 160 K
		440	**Ni-2b-240K-irr-160K-xtal-2**	after irradiation at 240 K

Table 2S. Selected X-ray data collection, processing and refinement parameters for all presented crystal structures.

Data set	**Ni-2a-290K-dark-cooling-xtal-1**	**Ni-2a-240K-dark - cooling-xtal-1**	**Ni-2a-200K-dark - cooling-xtal-1**	**Ni-2a-160K-dark - cooling-xtal-1**	**Ni-2a-140K-dark - cooling-xtal-1**	**Ni-2a-120K-dark - cooling-xtal-1**
Moiety formula	$C_{16}H_{15}N_3Ni_1O_3$					
Moiety formula mass, M_r / a.u.	356.00					
Crystal system	triclinic					
Space group	$P\bar{1}$ (no. 2)					
Z	2					
F_{000}	368					
Crystal colour & shape	orange plate					
Crystal size / mm^3	0.05×0.15×0.19					
T / K	290	240	200	160	140	120
a / Å	7.5324(3)	7.4868(3)	7.4560(4)	7.4308(4)	7.4166(4)	7.4036(5)
b / Å	8.1607(4)	8.1511(3)	8.1469(4)	8.1474(4)	8.1470(5)	8.1485(6)
c / Å	13.7040(7)	13.6670(6)	13.6490(7)	13.6372(7)	13.6222(8)	13.6104(9)
α / °	100.410(4)	100.337(3)	100.302(4)	100.277(4)	100.289(5)	100.310(6)
β / °	101.755(4)	101.551(3)	101.435(4)	101.262(4)	101.159(5)	101.086(6)
γ / °	102.921(4)	102.899(3)	102.851(4)	102.891(5)	102.899(5)	102.899(6)
V / Å^3	780.91(7)	774.28(6)	770.41(7)	767.74(7)	765.69(8)	764.01(10)
d_{calc} / g·cm^{-3}	1.5140	1.5270	1.5347	1.5400	1.5441	1.5475
θ range	3.39° – 76.17°	3.40° – 76.22°	3.40° – 75.98°	3.40° – 75.88°	3.40° – 75.73°	3.31° – 75.39°
Absorption coefficient, μ / mm^{-1}	1.956	1.973	1.983	1.990	1.995	2.000
No. of reflections collected / unique	9116 / 3168	9056 / 3145	8926 / 3128	8741 / 3112	8623 / 3102	8479 / 3083
R_{int}	3.00%	2.88%	3.29%	5.00%	5.40%	5.97%
No. of reflections with $I > 3\sigma(I)$	2483	2542	2517	2266	2218	2175
No. of parameters / restraints / constraints	209 / 0 / 60	209 / 0 / 60	209 / 0 / 60	216 / 2 / 70	219 / 0 / 67	219 / 0 / 67
$R[F]$ $(I > 3\sigma(I))$	3.71%	3.51%	3.54%	4.31%	4.48%	4.78%
$R[F]$ (all data)	4.95%	4.62%	4.61%	6.28%	6.60%	7.07%
$\varrho_{res}^{min/max}$ / e·Å^{-3}	−0.28 / +0.34	−0.31 / +0.38	−0.28 / +0.40	−0.31 / +0.42	−0.31 / +0.49	−0.36 / +0.53
CCDC code	2110810	2110811	2110818	2110820	2110819	2110812

Table 2S (continued). Selected X-ray data collection, processing and refinement parameters for all presented crystal structures.

Data set	**Ni-2a-100K-dark - cooling-xtal-1**	**Ni-2a-120K-dark - heating-xtal-1**	**Ni-2a-140K-dark - heating-xtal-1**	**Ni-2a-160K-dark - heating-xtal-1**	**Ni-2a-180K-dark - heating-xtal-1**	**Ni-2a-200K-dark - heating-xtal-1**
Moiety formula	$C_{16}H_{15}N_3Ni_1O_3$					
Moiety formula mass, M_r / a.u.	356.00					
Crystal system	triclinic					
Space group	$P\bar{1}$ (no. 2)					
Z	2					
F_{000}	368					
Crystal colour & shape	orange plate					
Crystal size / mm^3	0.05×0.15×0.19					
T / K	100	120	140	160	180	200
a / Å	7.4023(7)	7.4081(5)	7.4242(5)	7.4449(4)	7.4484(3)	7.4553(4)
b / Å	8.1523(7)	8.1507(6)	8.1555(6)	8.1616(5)	8.1532(4)	8.1473(4)
c / Å	13.6139(12)	13.6011(9)	13.6058(9)	13.6166(10)	13.6235(6)	13.6521(7)
α / °	100.305(7)	100.336(6)	100.383(6)	100.413(6)	100.394(4)	100.286(4)
β / °	101.078(8)	101.060(6)	101.096(5)	101.156(5)	101.263(3)	101.447(5)
γ / °	102.856(8)	102.871(6)	102.858(6)	102.845(5)	102.848(3)	102.865(5)
V / Å^3	764.64(13)	764.27(10)	766.41(10)	769.47(9)	769.10(6)	770.51(7)
d_{calc} / g·cm^{-3}	1.5462	1.5470	1.5427	1.5365	1.5373	1.5345
θ range	3.40° – 77.52°	3.40° – 76.21°	3.40° – 75.95°	3.40° – 75.75°	3.40° – 76.08°	3.40° – 76.00°
Absorption coefficient, μ / mm^{-1}	1.998	1.999	1.994	1.986	1.987	1.983
No. of reflections collected / unique	8471 / 3080	8479 / 3089	8603 / 3107	8705 / 3115	8808 / 3122	8912 / 3130
R_{int}	6.38%	5.97%	5.53%	5.25%	3.84%	3.20%
No. of reflections with $I > 3\sigma(I)$	2068	2173	2173	2215	2418	2508
No. of parameters / restraints / constraints	219 / 0 / 67	219 / 0 / 67	219 / 0 / 67	219 / 0 / 67	219 / 0 / 67	209 / 0 / 60
$R[F]$ $(I > 3\sigma(I))$	5.07%	4.83%	4.57%	4.55%	3.77%	3.64%
$R[F]$ (all data)	8.09%	7.36%	7.17%	6.83%	5.21%	4.80%
$\varrho_{res}^{min/max}$ / e·Å^{-3}	−0.47 / +0.65	−0.39 / +0.47	−0.34 / +0.43	−0.25 / +0.45	−0.25 / +0.45	−0.32 / +0.35
CCDC code	2110815	2110821	2110813	2110814	2110816	2110817

Table 2S (continued). Selected X-ray data collection, processing and refinement parameters for all presented crystal structures.

Data set	**Ni-2a-140K-dark-xtal-2**	**Ni-2a-140K-irr-140K-xtal-2**	**Ni-2a-160K-irr-140K-xtal-2**	**Ni-2a-180K-irr-140K-xtal-2**	**Ni-2a-200K-irr-140K-xtal-2**
Moiety formula	$C_{16}H_{15}N_3Ni_1O_3$				
Moiety formula mass, M_r / a.u.	356.00				
Crystal system	triclinic				
Space group	$P\bar{1}$ (no. 2)				
Z	2				
F_{000}	368				
Crystal colour & shape	orange plate				
Crystal size / mm³	0.05×0.15×0.25				
T / K	140	140	160	180	200
a / Å	7.4121(4)	7.4646(9)	7.4870(15)	7.426(4)	7.4497(3)
b / Å	8.1409(4)	8.1753(6)	8.1749(12)	8.166(3)	8.1435(3)
c / Å	13.6104(6)	13.5119(9)	13.5286(19)	13.623(8)	13.6386(5)
α / °	100.221(4)	100.600(6)	100.684(12)	100.88(4)	100.279(3)
β / °	101.157(4)	100.480(8)	100.490(14)	101.52(5)	101.386(4)
γ / °	102.885(5)	102.812(8)	102.696(15)	102.76(4)	102.913(4)
V / Å³	764.28(7)	768.69(13)	772.0(2)	765.7(7)	768.87(6)
d_{calc} / g·cm⁻³	1.5470	1.5381	1.5314	1.5441	1.5377
θ range	3.40° – 72.88°	3.42° – 72.96°	3.42° – 73.52°	3.41° – 73.45°	3.40° – 73.04°
Absorption coefficient, μ / mm⁻¹	1.999	1.988	1.979	1.995	1.987
No. of reflections collected / unique	11924/2996	11792/3017	10837/3028	10339/3004	11560/3021
R_{int}	4.33%	6.12%	7.62%	14.12%	4.50%
No. of reflections with $I > 3\sigma(I)$	2356	2078	1780	1037	2385
No. of parameters / restraints / constraints	209 / 0 / 60	219 / 0 / 67	216 / 0 / 70	208 / 0 / 60	208 / 0 / 60
$R[F]$ ($I > 3\sigma(I)$)	3.37%	6.52%	8.77%	13.05%	3.79%
$R[F]$ (all data)	4.79%	9.45%	13.89%	24.35%	5.05%
$\varrho_{res}^{min/max}$ / e·Å⁻³	−0.25 / +0.35	−0.49 / +0.87	−0.73 / +1.06	−1.20 / +3.14	−0.28 / +0.42
CCDC code	2110822	2110824	2110825	2110823	2110826

Table 2S (continued). Selected X-ray data collection, processing and refinement parameters for all presented crystal structures.

Data set	**Ni-2b-290K-dark-cooling-xtal-1**	**Ni-2b-240K-dark-cooling-xtal-1**	**Ni-2b-200K-dark-cooling-xtal-1**	**Ni-2b-180K-dark-cooling-xtal-1**	**Ni-2b-160K-dark-cooling-xtal-1**	**Ni-2b-RT-dark-xtal-2**
Moiety formula	$C_{19}H_{15}N_3Ni_1O_3$	$C_{19}H_{15}N_3Ni_1O_3$				$C_{19}H_{15}N_3Ni_1O_3$
Moiety formula mass, M_r / a.u.	392.04	392.04				392.04
Crystal system	monoclinic	triclinic				monoclinic
Space group	$P2_1/c$ (no. 14)	$P\bar{1}$ (no. 2)				$P2_1/c$ (no. 14)
Z	4	4				4
F_{000}	808	808				808
Crystal colour & shape	brown plate	brown plate				brown plate
Crystal size / mm^3	1.88×0.13×0.11	1.88×0.13×0.11				0.08×0.11×0.13
T / K	290	240	200	180	160	290
a / Å	14.1514(7)	14.1275(4)	14.1166(3)	14.1196(6)	14.1131(4)	14.1406(7)
b / Å	6.8027(3)	6.7861(12)	6.7699(8)	6.7595(13)	6.7551(10)	6.8044(3)
c / Å	17.9707(6)	17.9317(9)	17.8897(8)	17.8685(11)	17.8553(10)	17.9675(7)
α / °	90	89.017(6)	88.801(4)	88.760(7)	88.714(5)	90
β / °	103.885(4)	103.987(5)	104.046(4)	104.018(6)	104.113(5)	103.816(4)
γ / °	90	93.019(6)	93.747(4)	93.966(7)	94.140(5)	90
V / Å^3	1679.45(13)	1665.8(3)	1655.0(2)	1650.6(3)	1646.5(3)	1678.78(13)
d_{calc} / g·cm^{-3}	1.5505	1.5632	1.5734	1.5776	1.5818	1.5511
θ range	3.22° – 75.55°	2.54° – 75.56°	2.55° – 75.59°	2.55° – 80.79°	2.55° – 75.77°	3.22° – 75.61°
Absorption coefficient, μ / mm^{-1}	1.884	1.899	1.912	1.917	1.921	1.884
No. of reflections collected / unique	12943 / 3428	13035 / 6230	13044 / 6192	12983 / 6182	12887 / 6140	13530 / 3452
R_{int}	3.76%	6.07%	6.27%	8.93%	6.44%	2.72%
No. of reflections with $I > 3\sigma(I)$	2309	3179	3570	2782	3358	2375
No. of parameters / restraints / constraints	235 / 0 / 60	469 / 0 / 120	469 / 0 / 120	469 / 0 / 120	469 / 0 / 120	235 / 0 / 60
$R[F]$ $(I > 3\sigma(I))$	5.13%	9.61%	8.22%	11.07%	9.36%	4.36%
$R[F]$ (all data)	7.21%	14.44%	12.10%	16.53%	13.94%	6.54%
$\varrho_{res}^{min/max}$ / e·Å^{-3}	−0.66 / +0.65	−0.72 / +1.98	−0.64 / +1.95	−1.41 / +2.07	−0.91 / +2.27	−0.53 / +0.42
CCDC code	2110831	2110833	2110832	2110835	2110834	2110827

Table 2S (continued). Selected X-ray data collection, processing and refinement parameters for all presented crystal structures.

Data set	**Ni-2b-160K-dark-xtal-2**	**Ni-2b-160K-irr-160K-xtal-2**	**Ni-2b-240K-irr-160K-xtal-2**	**Cu-2a**	**Cu-2b**
Moiety formula	$C_{19}H_{15}N_3Ni_1O_3$			$C_{16}H_{15}N_3Cu_1O_3$	$C_{19}H_{15}N_3Cu_1O_3$
Moiety formula mass, M_r / a.u.	392.04			360.86	396.89
Crystal system	triclinic			triclinic	triclinic
Space group	$P\bar{1}$ (no. 2)			$P\bar{1}$ (no. 2)	$P\bar{1}$ (no. 2)
Z	4			2	2
F_{000}	808			370	406
Crystal colour & shape	brown plate			brown plate	green plate
Crystal size / mm^3	0.08×0.11×0.13			0.05×0.19×0.22	0.04×0.11×0.20
T / K	160	160	240	100	100
a / Å	14.1083(10)	14.1508(14)	14.1382(13)	8.435(2)	6.804(2)
b / Å	6.7505(3)	6.7652(5)	6.8015(8)	9.513(2)	9.088(2)
c / Å	17.8681(8)	17.8650(9)	17.9478(8)	10.739(2)	13.866(3)
α / °	88.724(3)	90.025(5)	89.744(7)	69.07(3)	75.87(3)
β / °	104.156(5)	103.718(6)	103.931(6)	69.65(3)	83.43(3)
γ / °	94.061(4)	90.165(7)	90.193(9)	72.48(3)	84.76(3)
V / Å^3	1645.88(16)	1661.5(2)	1675.1(3)	739.0(3)	824.2(4)
d_{calc} / g·cm^{-3}	1.5821	1.5673	1.5545	1.6216	1.5992
θ range	2.55° – 75.75°	2.55° – 75.59°	2.54° – 77.80°	4.58° – 75.56°	3.30° – 75.76°
Absorption coefficient, μ / mm^{-1}	1.922	1.904	1.889	2.260	2.091
No. of reflections collected / unique	18886 / 6654	10519 / 5836	19389 / 6800	17061 / 3031	17819 / 3393
R_{int}	6.15%	3.62%	8.49%	4.31%	10.28%
No. of reflections with $I > 3\sigma(I)$	3505	3136	2935	2791	2818
No. of parameters / restraints / constraints	469 / 0 / 120	459 / 0 / 196	469 / 0 / 120	208 / 0 / 60	235 / 0 / 60
$R[F]$ $(I > 3\sigma(I))$	11.73%	10.90%	6.89%	3.33%	5.46%
$R[F]$ (all data)	17.23%	16.51%	12.63%	3.60%	6.43%
$\varrho_{res}^{min/max}$ / e·Å^{-3}	−1.15 / +3.36	−1.22 / +1.66	−0.82 / +1.16	−0.38 / +0.48	−0.78 / +0.98
CCDC code	2110828	2110829	2110830	2110808	2110809

Table 3S. Selected interactions engaging the nitro group adopting both the nitro and *endo*-nitrito binding modes in the **Ni-2a** crystal structure (**Ni-2a-140K-irr-140K-xtal-2** data set).

nitro – nitro			*endo*-nitrito – *endo*-nitrito		
Motif	E_{int} / kJ·mol^{-1}	*Selected interactions*	*Motif*	E_{int} / kJ·mol^{-1}	*Selected interactions*
**Ni$_{O1}$**	−60.5	C1–H1···O1a<br>C2–H2···O1a	**Ni$_{O1}$**	−48.4	C1–H1···O1b<br>C2–H2···O1b
**Ni$_{O2}$**	−24.3	C7–H7···O2a	–	–	–
**Ni$_{S1}$**	−44.7	C14–H14···O1a<br>C11(π) ···C14(π)	**Ni$_{S1}$**	−45.9	C14–H14···O1b<br>C11(π) ···C14(π)
**Ni$_{S2}$**	−126.0	C4–H4···O1a<br>C6–H6b···O1a<br>C1(π)···C8(π)	**Ni$_{S2}$**	−132.4	C4–H4···O1b<br>C6–H6b···O1b<br>C1(π)···C8(π)
**Ni$_{S2'}$**	−142.1	C6–H6a···O2a<br>C1(π)···C8(π)	**Ni$_{S2'}$**	−128.1	C6–H6a···O2b<br>C1(π) ···C8(π)

Table 3S (continued). Selected interactions engaging the nitro group adopting both the nitro and *endo*-nitrito binding modes in the **Ni-2a** crystal structure (**Ni-2a-140K-irr-140K-xtal-2** data set).

nitro – nitro			*endo*-nitrito – *endo*-nitrito		
Motif	E_{int} / kJ·mol^{-1}	*Selected interactions*	*Motif*	E_{int} / kJ·mol^{-1}	*Selected interactions*
**Ni$_{O1}$**	−54.3	C1–H1···O1a<br>C2–H2···O1a	**Ni$_{O1}$**	−48.4	C1–H1···O1b<br>C2–H2···O1b
**Ni$_{O2}$**	−23.9	C7–H7···O2a	–	–	–
**Ni$_{S1}$**	−45.3	C14–H14···O1a<br>C11(π)···C14(π)	**Ni$_{S1}$**	−45.3	C14–H14···O1b<br>C11(π)···C14(π)
**Ni$_{S2}$**	−129.2	C4–H4···O1a<br>C6–H6b···O1a<br>C1(π)···C8(π)	**Ni$_{S2}$**	−129.2	C4–H4···O1b<br>C6–H6b···O1b<br>C1(π)···C8(π)
**Ni$_{S2'}$**	−129.2	C6–H6a···O2a<br>C1(π)···C8(π)	**Ni$_{S2'}$**	−128.1	C6–H6a···O2b<br>C1(π)···C8(π)

Table 4S. Selected intermolecular interactions for both nitro and *endo*-nitrito isomers for the **Ni-2b** complex (**Ni-2b-160K-irr-160K-xtal-2** data set).

nitro – nitro			*endo*-nitrito – *endo*-nitrito		
Motif	E_{int} / kJ·mol^{-1}	*Selected interactions*	*Motif*	E_{int} / kJ·mol^{-1}	*Selected interactions*
**Ni$_{O1}$**	−39.6	C2–H2···O4<br>C2'–H2'···O2	**Ni$_{O1}$**	−37.0	C2–H2···O4<br>C2'–H2'···O2
**Ni$_{O3}$**	−44.6	C13–H13···O3'<br>C15–H15···O4	**Ni$_{O3}$**	−43.5	C13–H13···O3<br>C15–H15···O4
**Ni$_{S2}$**	−95.1	C5(π)···C6(π)	**Ni$_{S2}$**	−93.1	C5(π)···C6(π)
**Ni$_{S2'}$**	−94.6	C5'(π)···C6'(π)	**Ni$_{S2'}$**	−91.0	C5'(π)···C6'(π)

Table 4S (continued). Selected intermolecular interactions for both nitro and *endo*-nitrito isomers for the **Ni-2b** complex (**Ni-2b-160K-irr-160K-xtal-2** data set).

nitro – nitro			*endo*-nitrito – *endo*-nitrito		
Motif	E_{int} / kJ·mol^{-1}	*Selected interactions*	*Motif*	E_{int} / kJ·mol^{-1}	*Selected interactions*
**Ni$_{O1}$**	−38.2	C2–H2···O4<br>C2'–H2'···O2	**Ni$_{O1}$**	−38.1	C2–H2···O4<br>C2'–H2'···O2
**Ni$_{O3}$**	−44.3	C13–H13···O3'<br>C15–H15···O4	**Ni$_{O3}$**	−43.8	C13–H13···O3<br>C15–H15···O4
**Ni$_{S2}$**	−94.1	C5(π)···C6(π)	**Ni$_{S2}$**	−94.1	C5(π)···C6(π)
**Ni$_{S2'}$**	−92.8	C5'(π)···C6'(π)	**Ni$_{S2'}$**	−87.7	C5'(π)···C6'(π)

Table 5S. Selected intermolecular interactions for the **Ni-2b** complex at RT (*i.e.* before phase transition; **Ni-2b-RT-dark-xtal-2** data set).

Motif	E_{int} / kJ·mol^{-1}	*Selected interactions*
**Ni$_{O1}$**	−41.3	C2–H2···O1
**Ni$_{O3}$**	−43.1	C13–H13···O1<br>C15–H15···O3
**Ni$_{S2}$**	−95.9	C5(π)···C6(π)

Table 6S. Percentage contribution of selected interatomic contacts to the generated Hirshfeld surface.

Compound	*Temperature* / K	O–H	C–H	N–H	H–O	H–C	H–N	C–C	H–H
**Ni-2a** [a]	100	11.3	7.3	2.5	10.1	5.6	2.5	5.4	46.3
**Cu-2a**	100	10.0	14.1	6.7	8.1	11.9	5.3	1.9	37.6
**Ni-2b** (high *T*) [b]	290	13.5	9.5	0.3	11.5	8.1	0.2	4.9	41.7
**Ni-2b** (*mol. A*) (low *T*) [c]	160	13.3	9.4	0.4	11.2	8.0	0.3	5.2	41.4
**Ni-2b** (*mol. B*) (low *T*) [c]	160	13.4	9.6	0.5	11.4	8.0	0.5	5.4	41.0
**Cu-2b**	100	10.3	8.2	4.3	8.2	6.5	3.9	7.0	41.7

[a] **Ni-2a-100K-dark-cooling-xtal-1** data set. [b] **Ni-2b-290K-dark-cooling-xtal-1** data set. [c] **Ni-2b-160K-dark-cooling-xtal-1**.

Table 7S. Hirshfeld surface fingerprint plots generated for the **Ni-2b** complex; all and selected X···H contacts (data sets the same as in Table 6S).

Molecule	*all···all*	O···H	N···H	C···H
**Ni-2b** (high *T*) [b]	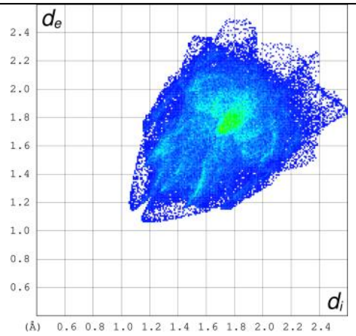	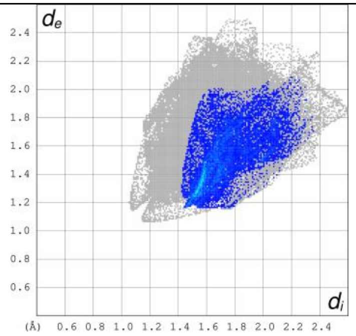	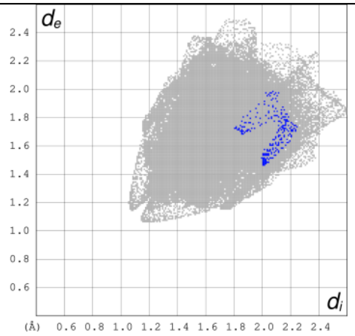	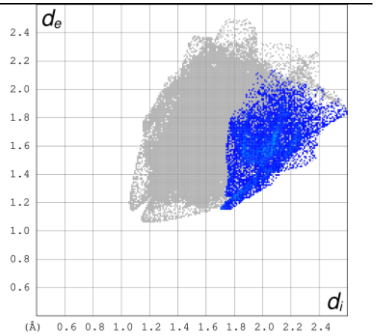
**Ni-2b** (*mol. A*) (low *T*) [c]	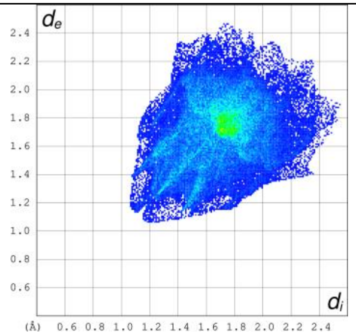	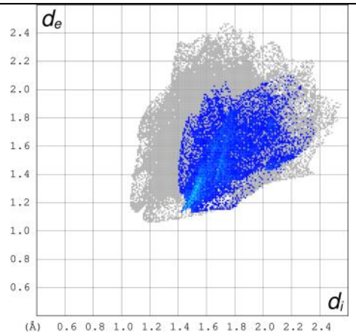	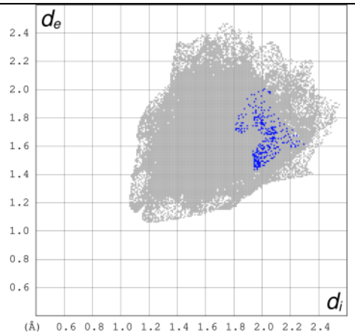	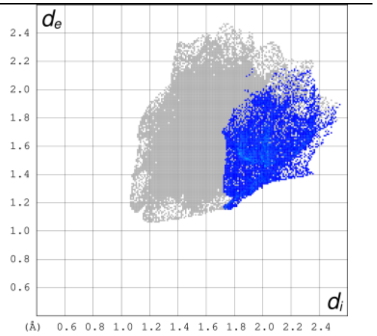
**Ni-2b** (*mol. B*) (low *T*) [c]	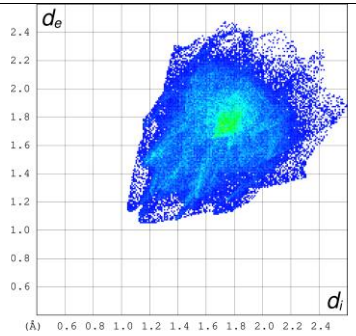	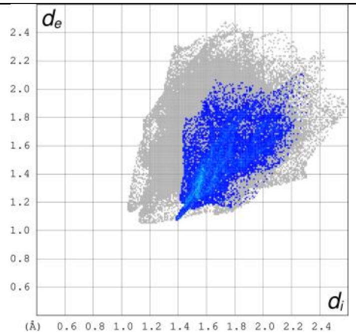	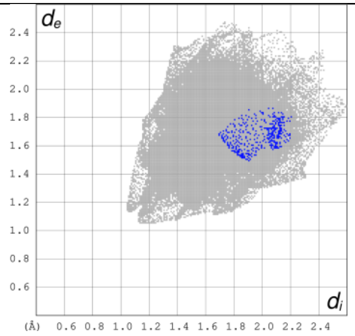	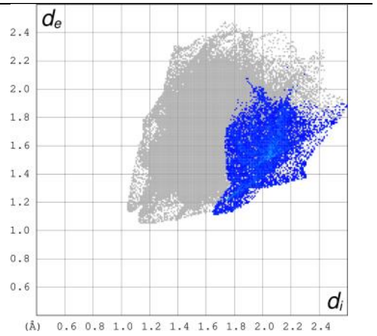

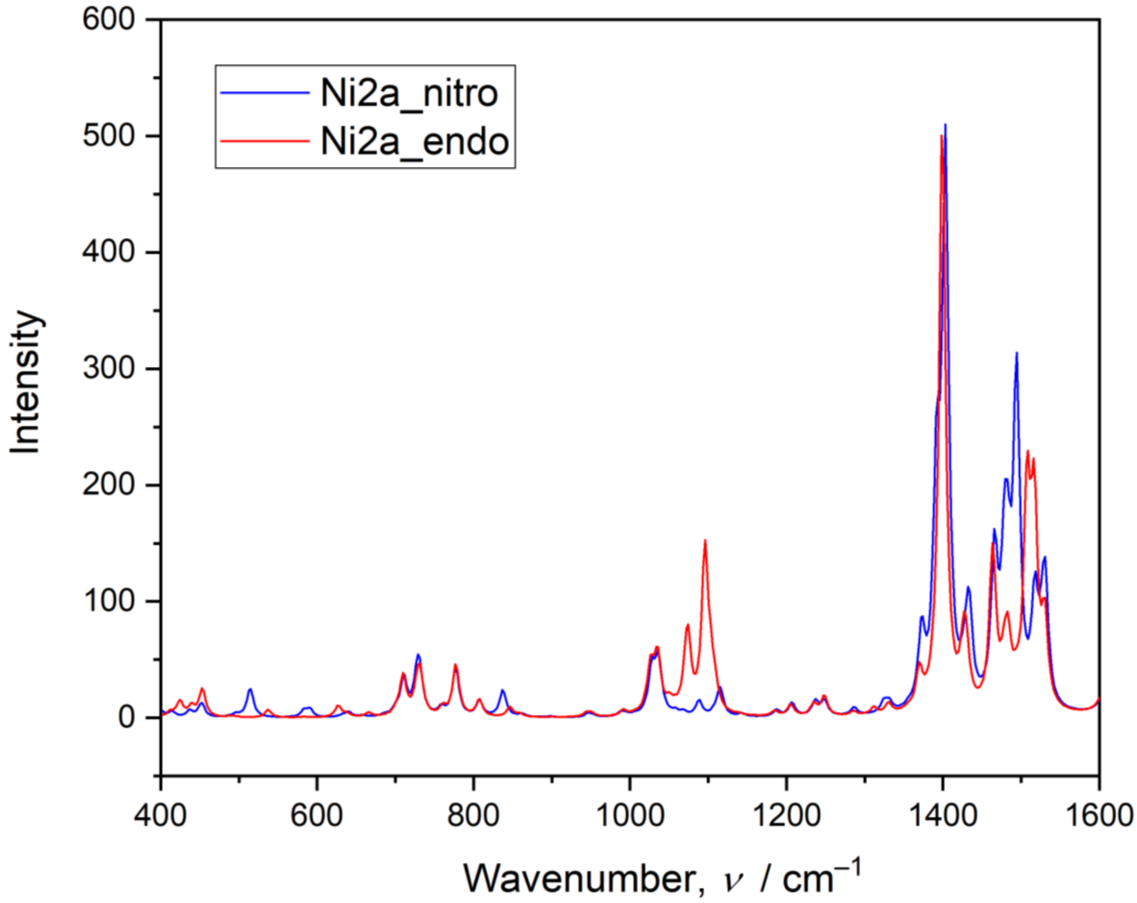

Figure 6S. Theoretical IR spectra computed at the DFT(B3LYP)/6-311++G** level of theory for the analysed **Ni-2a** linkage isomers plotted using the *GAUSSSUM* program (full width at half maximum set to 10).

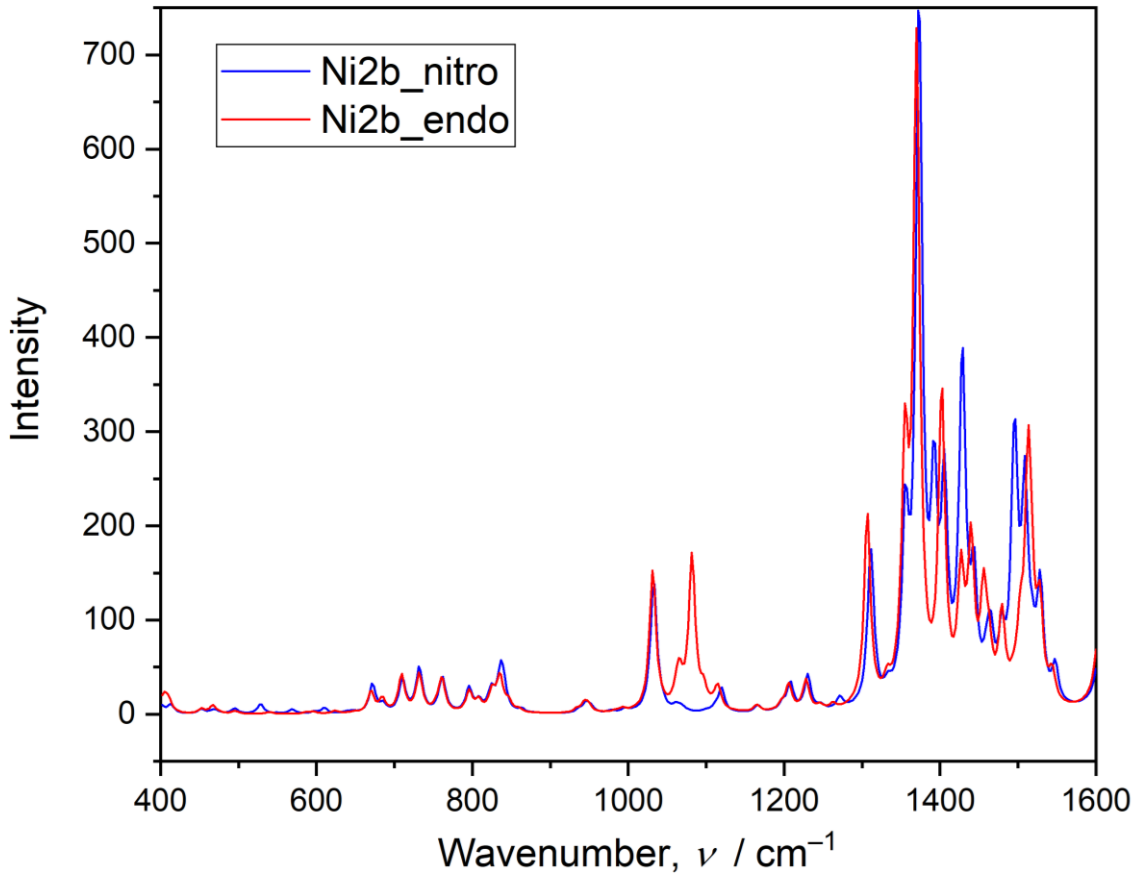

Figure 7S. Theoretical IR spectrum computed at the DFT(B3LYP)/6-311++G** level of theory for the analysed **Ni-2b** linkage isomers plotted using the *GAUSSSUM* program (full width at half maximum set to 10).

Table 8S. Key vibrational modes characteristic for the examined linkage isomers of the nitro group based on the theoretical data. Visualization of selected modes is attached in the form of GIF files.

Mode	**Ni-2a** (nitro)	**Ni-2b** (nitro)
$\omega(NO_2)$	581.02	566.70
$\delta(NO_2)$	835.94	838.41
$\nu_s(NO_2)$	1391.62	1391.96
$\nu_{as}(NO_2)$	1493.96	1495.36
	**Ni-2a** (*endo*-nitrito)	**Ni-2b** (*endo*-nitrito)
$\delta(ONO)$	845.05	845.04
$\nu(N-O)$	1094.74	1080.76
$\nu(N=O)$	1507.30	1513.02
	**Cu-2a** (κ-nitrito)	**Cu-2b** (κ-nitrito)
$\omega(ONO)$	347.65	350.89
$\delta(ONO)$	855.42	856.02
$\nu(N-O)$	1154.95	1150.47
$\nu(N=O)$	1454.98	1454.97
	**Cu-2a** (nitro)	**Cu-2b** (nitro)
$\delta(NO_2)$	820.15	820.36
$\nu_s(NO_2)$	1366.82	1369.82
$\nu_{as}(NO_2)$	1481.48	1487.37

Table 9S. IR spectral features characteristic for selected binding modes of the NO_2 group assigned based on the literature-reports of nickel(II) and copper(II) compounds. Note no information about $\omega(NO_2)$ were given.

Compound	*Isomer*	$\nu_a(NO_2)$	$\nu_s(NO_2)$	$\rho_w(ONO)$	ν(N=O)	ν(N–O)
$[Ni(py)_4(ONO)_2]$ [1]	ONO	1393	1114	825	–	–
$NiL_2(NO_2)_2$ L=[4-(2-aminoethyl)morpholine] [2]	NO_2	1333	1310	808	–	–
$[NiL_2(ONO)_2$ L=[4-(2-aminoethyl)morpholine] [2]	ONO	1365, 1331	1318, 1304	828	–	–
Ni[1-(2-aminoethyl)piperidine]$_2(NO_2)_2$ [3]	NO_2	1360	1285	815	–	–
Ni[1-(2-aminoethyl)piperidine]$_2(ONO)_2$ [3]	ONO	1385	1138	820	–	–
$Ni(C_8H_{18}N_4O_2)(NO_2)_2]\cdot H_2O$ [4]	NO_2	1395	1274	814	–	–
$Ni(C_8H_{18}N_4O_2)(ONO)_2]\cdot H_2O$ [4]	ONO	1270	1269	798	1412	1208
$[Cu(ONO)(tpa)]PF_6$ tpa= tris[(2-pyridyl)methyl]amine [5]	ONO	–	–	–	1426	1082
$[Cu(NO2)(tpa)]PF_6$ tpa = tris[(2-pyridyl)methyl]amine [5]	NO_2	1387	1333	–	–	–
(^{i}Pr-TPM)$Cu(NO_2)$ TPM= tris(3,5-diisopropyl-1-pyrazolyl)methane [6]	NO_2	1311	1282	814	–	–
(Et-TPM)$Cu(NO_2)$ TPM= tris(3,5-diethyl-1-pyrazolyl)methane [6]	NO_2	1310	1283	813	–	–
(Me-TPM)$Cu(NO_2)$ TPM= tris(3,5-dimethyl-1-pyrazolyl)methane [6]	NO_2	1314	1280	822	–	–
(^{i}Pr-TACN)$Cu(NO_2)$ TAC= 1,4,7-triisopropyl-1,4,7-triazacyclononane [6]	NO_2	1306	1268	809	–	–

References

[1] D. M. L. Goodgame, L. M. Venanzi, *J. Chem. Soc.* **1963**, 616-627.

[2] T. Chattopadhyay, M. Ghosh, A. Majee, M. Nethaji, D. Das, *Polyhedron* **2005**, 24, 1677-1681.

[3] D. Das, I. R. Laskar, A. Ghosh, A. Mondal, K.-i. Okamoto, N. R. Chaudhuri, *J. Chem. Soc., Dalton Trans.* **1998**, 3987-3990.

[4] M. S. Chao, H. H. Lu, M. L. Tsai, C. M. Lin, M. P. Wu, *Inorg. Chem. Comm.* **2012**, 24, 254–258.

[5] K. Nobutoshi, N. Hirotaka, K. Yoshinori, A. Gin-ya, S. Masatatsu, U. Akira, T. Koji, *Bull. Chem. Soc. Jpn* **1995**, 68, 581-589.

[6] M. Kujime, C. Izumi, M. Tomura, M. Hada, H. Fujii, J. Am. Chem. Soc. **2008**, 130, 6088-6098.

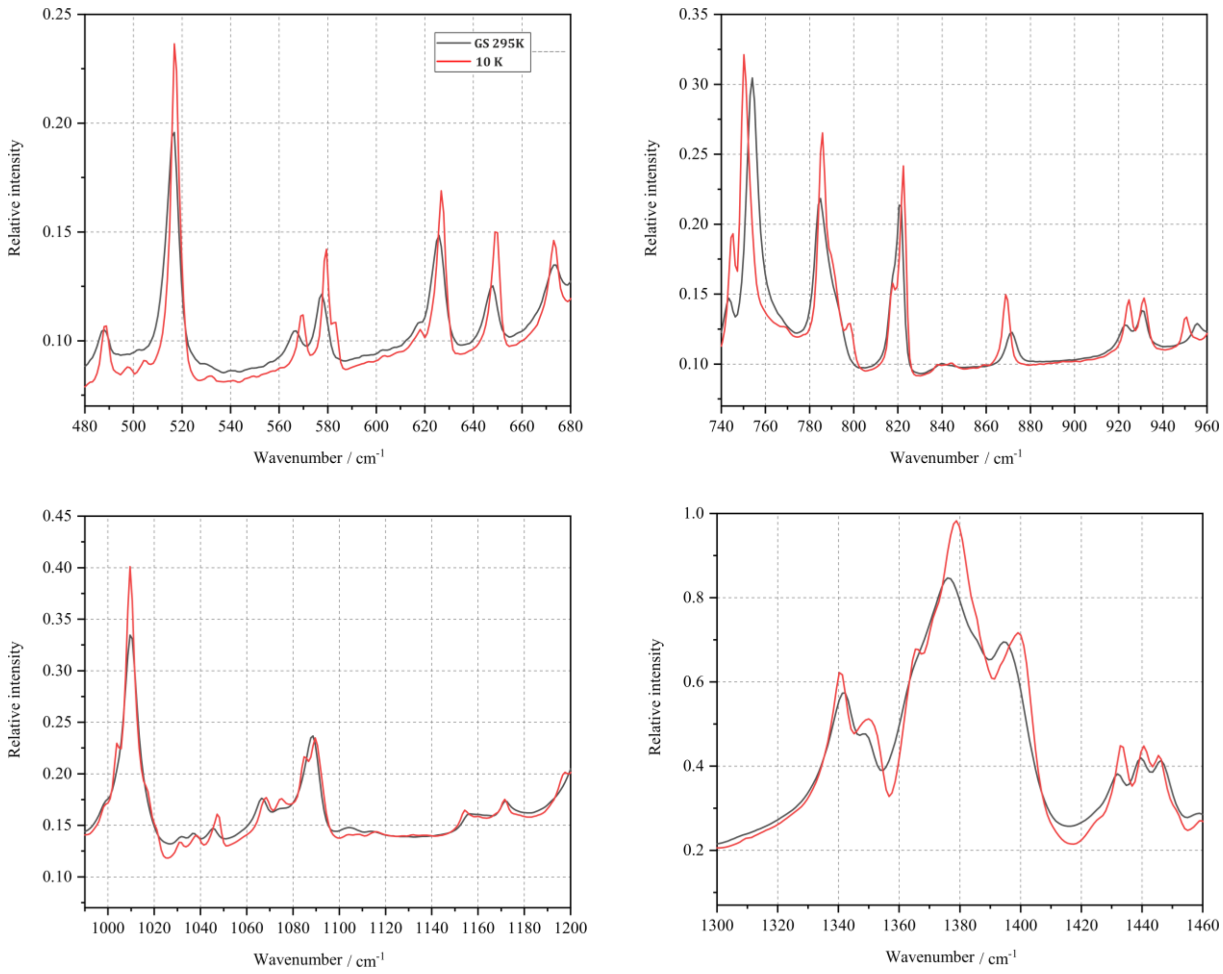

Figure 8S. IR absorption spectra for the **Ni-2a** complex in the solid state recorded while cooling from 295 K to 10 K. As indicted in the legend the black solid line denotes the 295 K measurement, while red lines present spectra recorded after sample cooling down to 10 K.

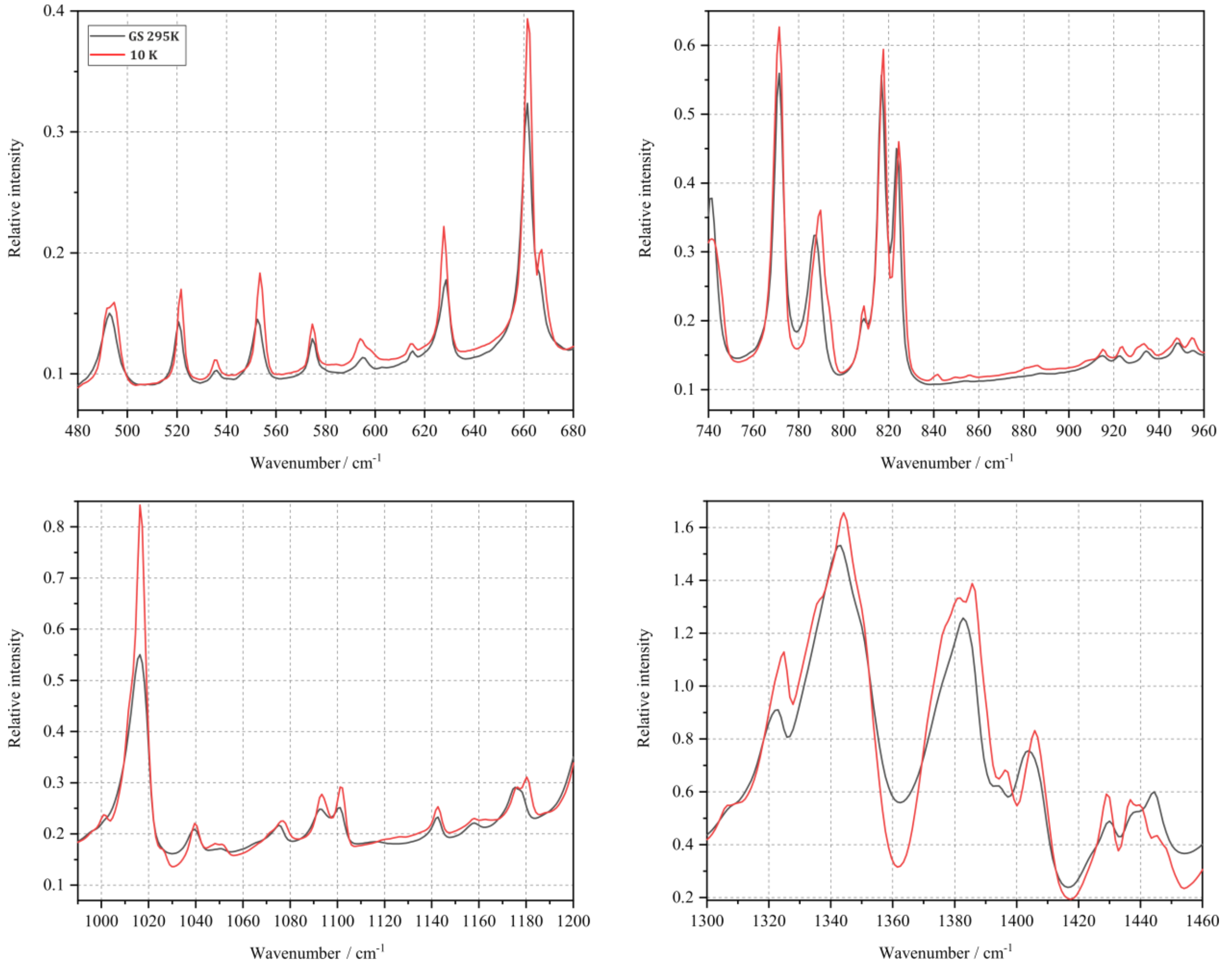

Figure 9S. IR absorption spectra for the **Ni-2b** complex in the solid state recorded while cooling from 295 K to 10 K. As indicted in the legend the black solid line denotes the 295 K measurement, while red lines present spectra recorded after sample cooling down to 10 K.

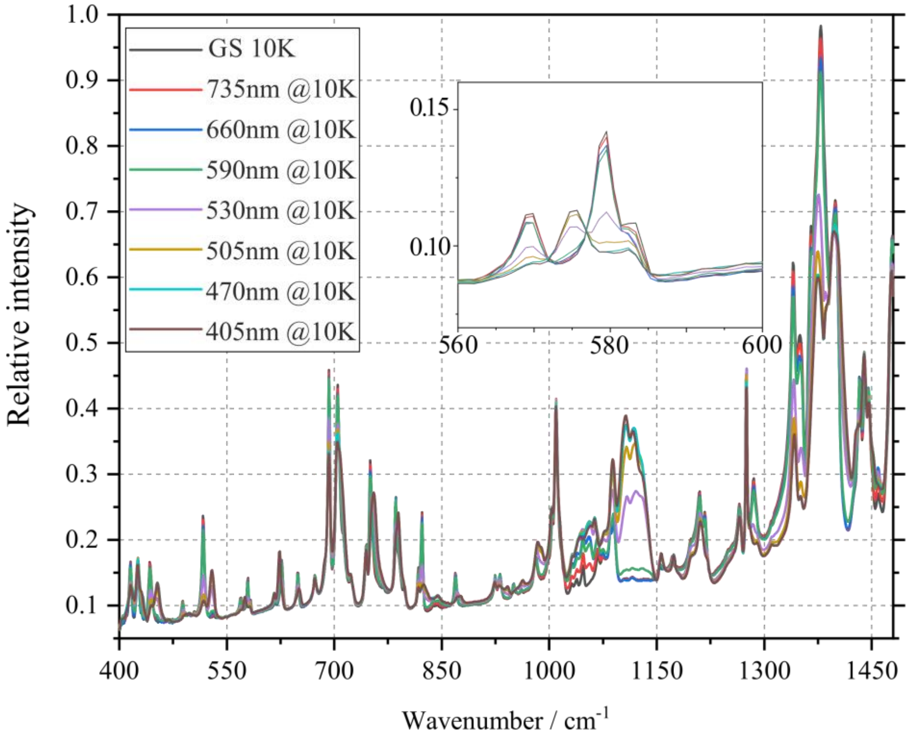

(*a*)

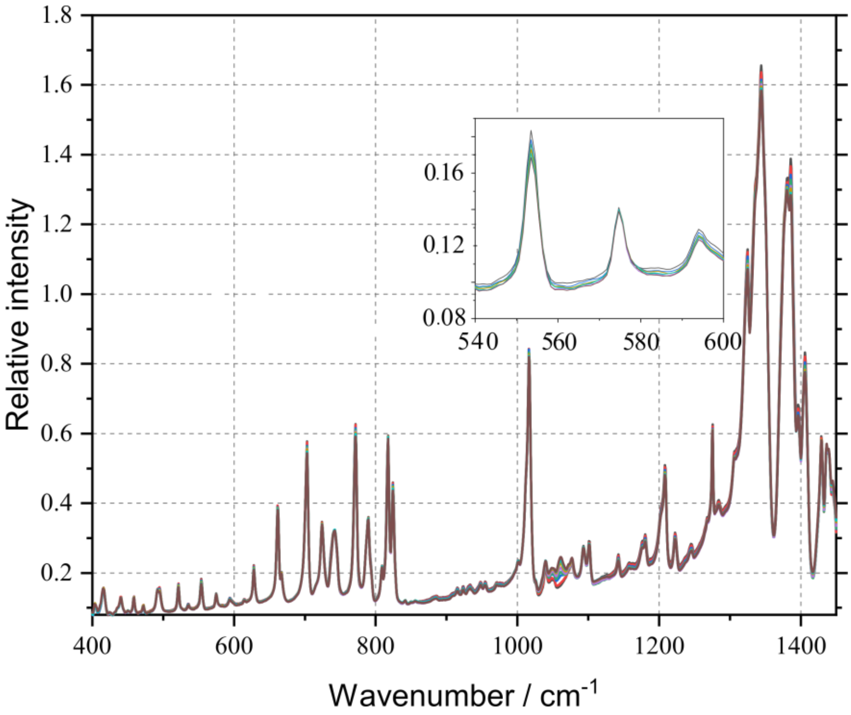

(*b*)

Figure 10S. IR absorption spectra for the (*a*) **Ni-2a** and (*b*) **Ni-2b** complexes in the solid state recorded at 10 K after irradiation with the LED light (405–735 nm).

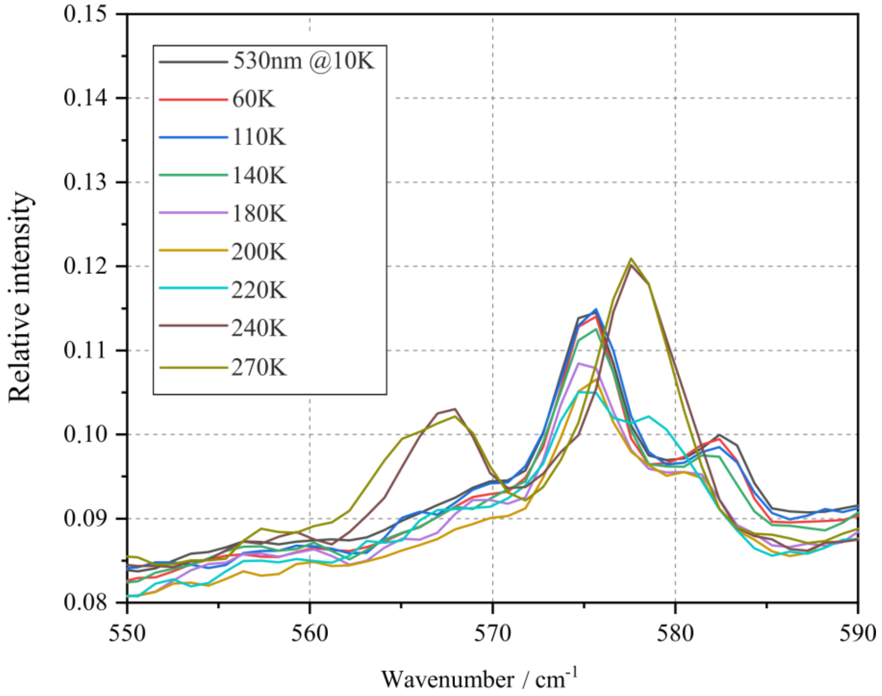

Figure 11S. Multi-temperature IR spectrum recorded after irradiation at 530 nm at 10 K and at selected temperature points during heating of the **Ni-2a** sample; intensity changes of the 567 cm^{-1} peak are shown.

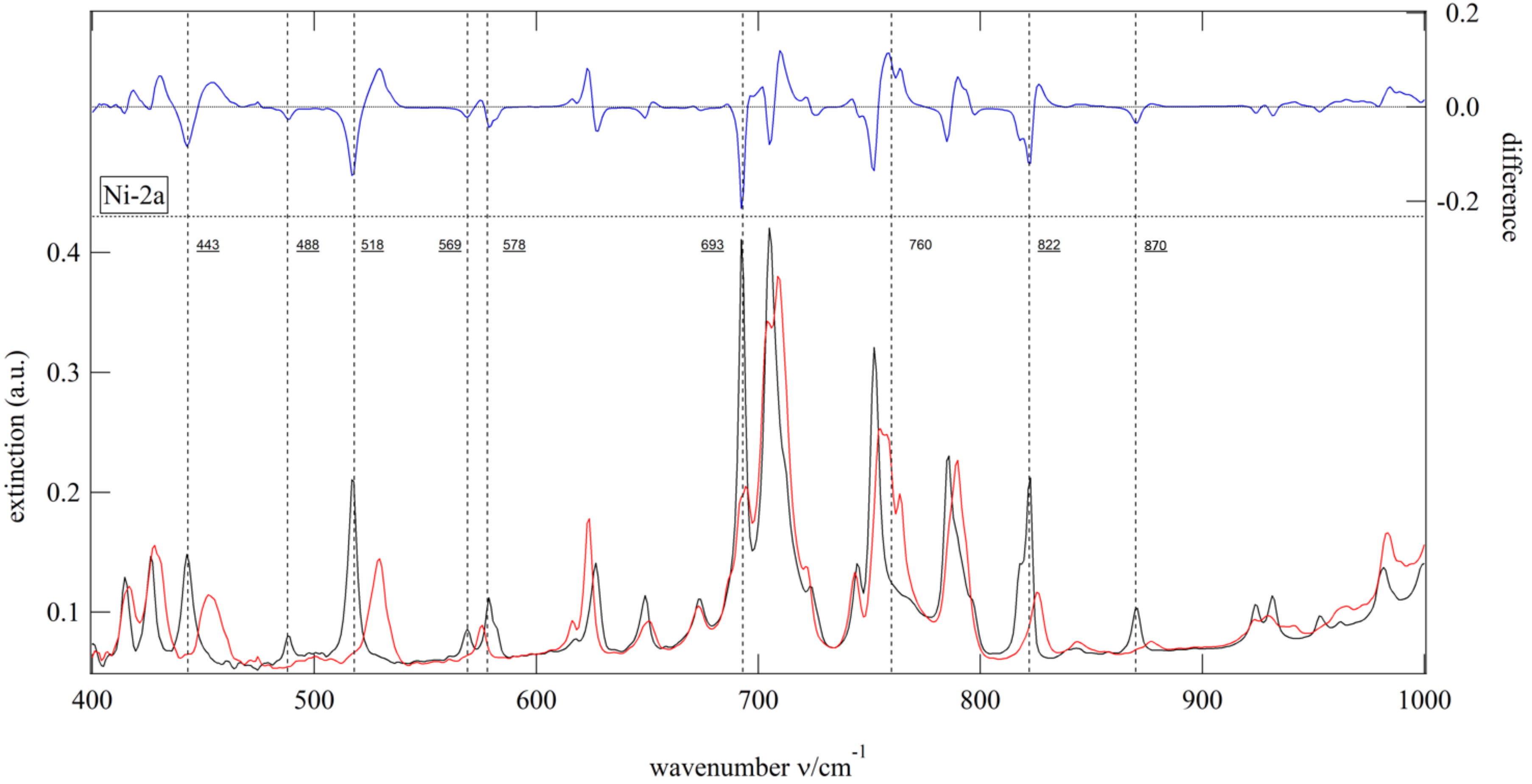

Figure 12S. Expansion of the 400–1000 cm^{-1} region of the **Ni-2a** complex IR absorption spectra. Black solid line denotes the ground state spectrum, red line presents the spectrum collected after 10 min of LED irradiation, while blue line shows the difference spectrum. Observations with respect to the computational results (Table 8S): decrease of the bands at 569/578 cm^{-1} and 822/870 cm^{-1} corresponding to the $\omega(NO_2)$ and $\delta(NO_2)$ modes of the nitro configuration. Identification of genuinely new bands due to the *endo*-nitrito form is not obvious, as many bands might simply shift as a consequence of the NO_2 isomerisation, *e.g.* from 518 cm^{-1} to 529 cm^{-1}.

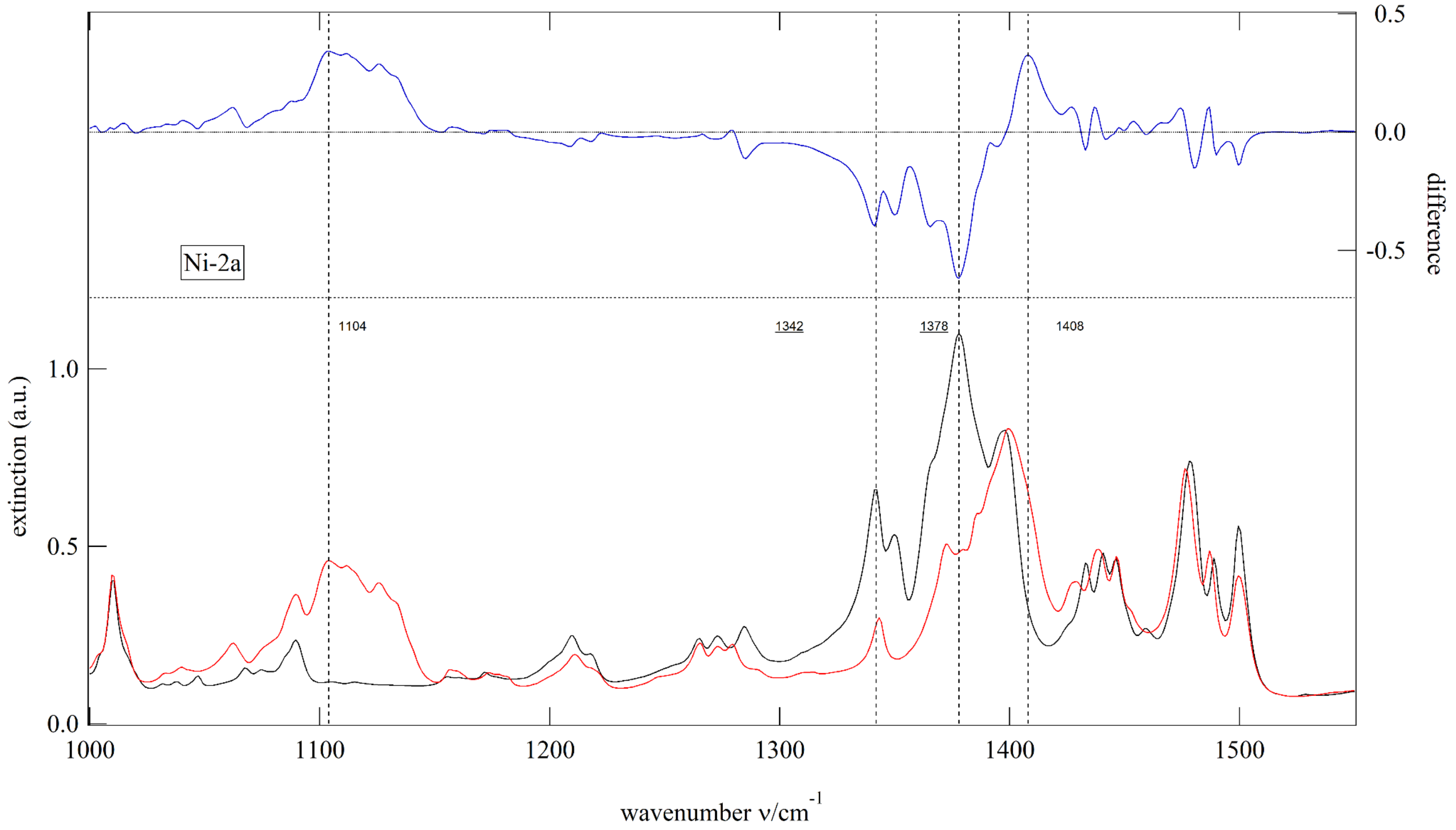

Figure 13S. Expansion of the 1000–1550 cm^{-1} region of the **Ni-2a** complex IR absorption spectra. Black solid line denotes the ground state spectrum, red line presents the spectrum collected after 10 min of LED irradiation, while blue line shows the difference spectrum. Observations with respect to the computational results (Table 8S): decrease of the bands at 1342 and 1378 cm^{-1} corresponding to the $\nu_s(NO_2)$ and $\nu_{as}(NO_2)$ modes of the nitro configuration. Appearance of a new band at 1104 cm^{-1} corresponding to the ν(N–O) mode of the *endo*-nitrito configuration.

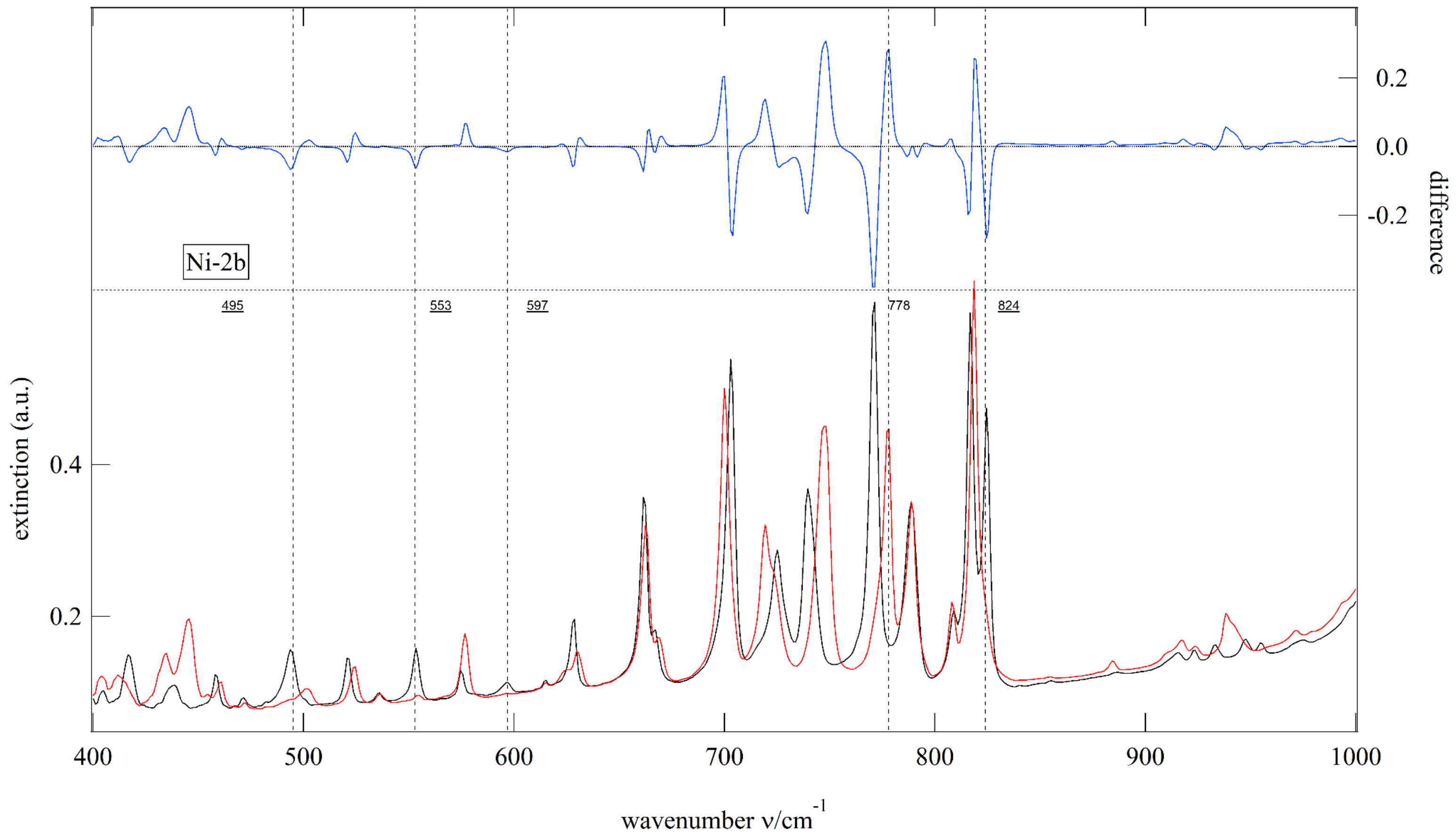

Figure 14S. Expansion of the 400–1000 cm^{-1} region of the IR for **Ni-2b** complex IR absorption spectra. Black solid line denotes the ground state spectrum, red line presents the spectrum collected after 10 min of LED irradiation, while blue line shows the difference spectrum. Observations with respect to computational results (Table 8S): decrease of the bands at 553/597 cm^{-1} and 824 cm^{-1} corresponding to the $\omega(NO_2)$ and $\delta(NO_2)$ modes of the nitro configuration. Identification of genuinely new bands due to the *endo*-nitrito form is not obvious, as many bands might simply shift as a consequence of the NO_2 isomerisation, *e.g.* from 771 cm^{-1} to 778 cm^{-1}.

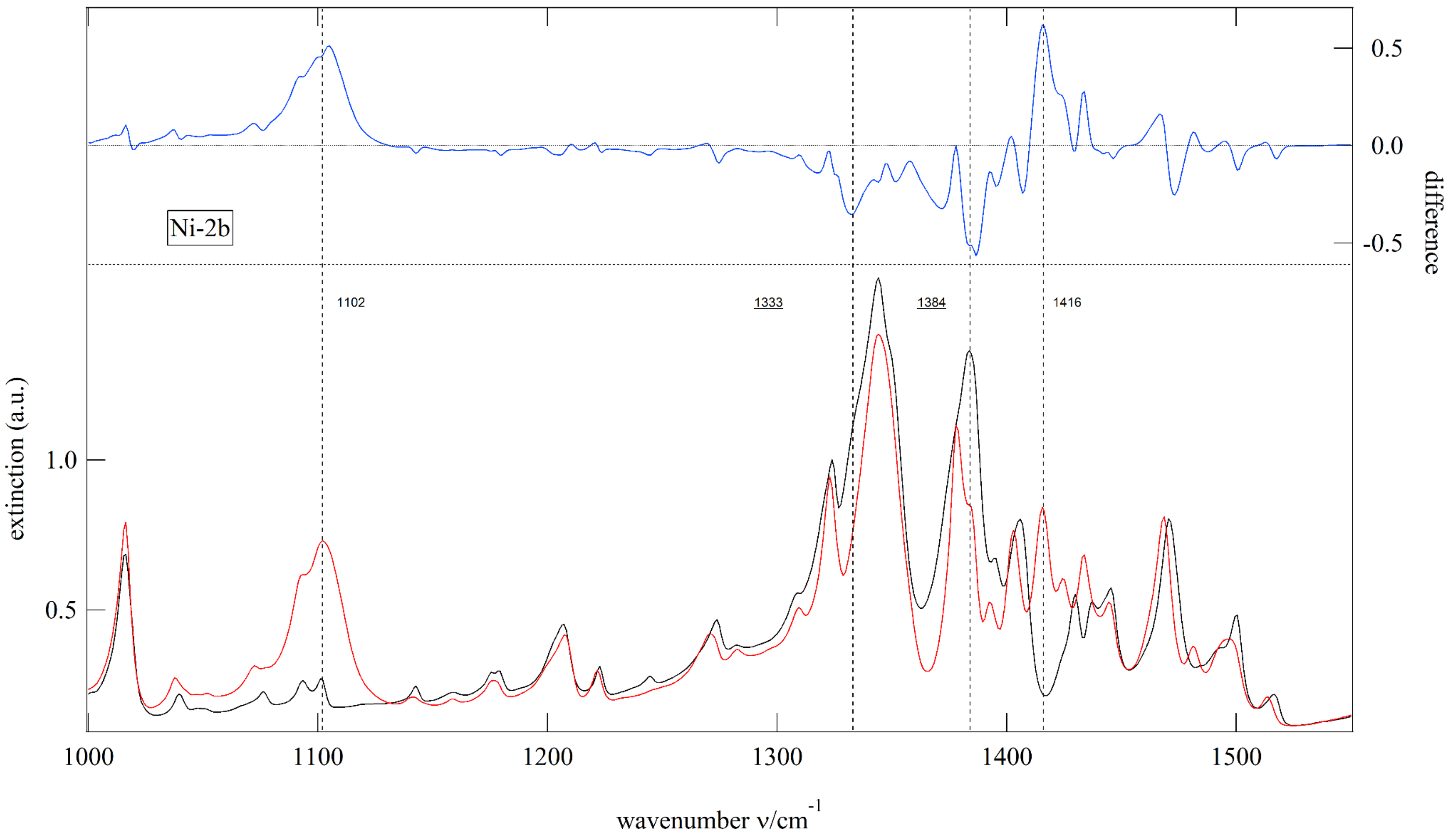

Figure 15S. Expansion of the 1000–1550 cm^{-1} region of the **Ni-2b** complex IR absorption spectra. Black solid line denotes the ground state spectrum, red line presents the spectrum collected after 10 min of LED irradiation, while blue line shows the difference spectrum. Observations with respect to the computational results (Table 8S): decrease of the bands at 1333 and 1384 cm^{-1} corresponding to the $\nu_s(NO_2)$ and $\nu_{as}(NO_2)$ modes of the nitro configuration. Appearance of a new band at 1102 cm^{-1} corresponding to the ν(N–O) mode of the *endo*-nitrito configuration.

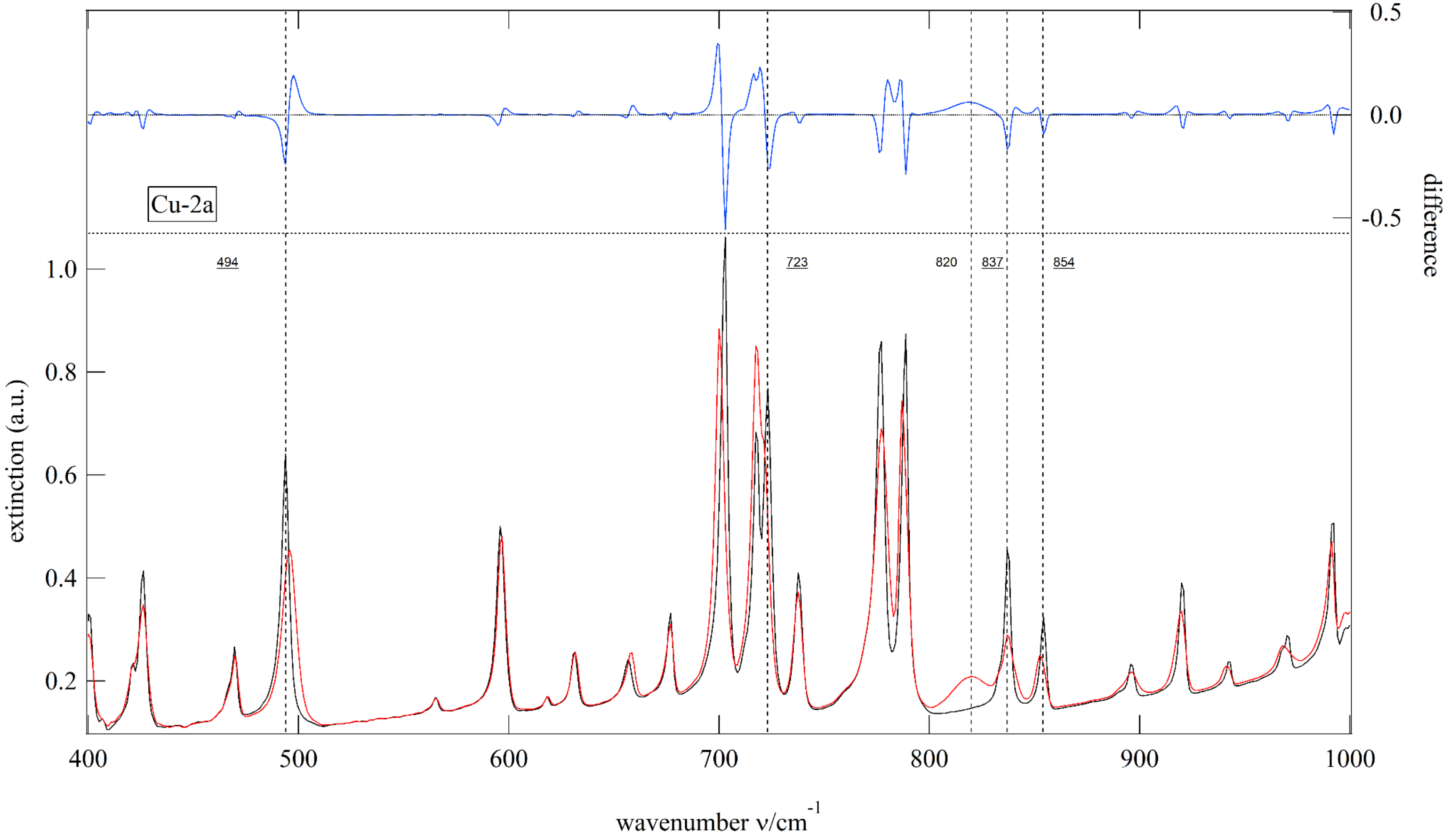

Figure 16S. Expansion of the 400−1000 cm^{-1} region of the **Cu-2a** complex IR absorption spectra. Black solid line denotes the ground state spectrum, red line presents the spectrum collected after 10 min of LED irradiation, while blue line shows the difference spectrum. Observations with respect to the computational results (Table 8S): decrease of the bands at 837 and 854 cm^{-1} corresponding to the δ(ONO) mode of the κ-nitrito configuration and appearance of a new band at 820 cm^{-1} corresponding to the δ(NO_2) mode of the nitro configuration. The other observable changes correspond to shifts induced by the ONO isomerisation on the other ligand groups.

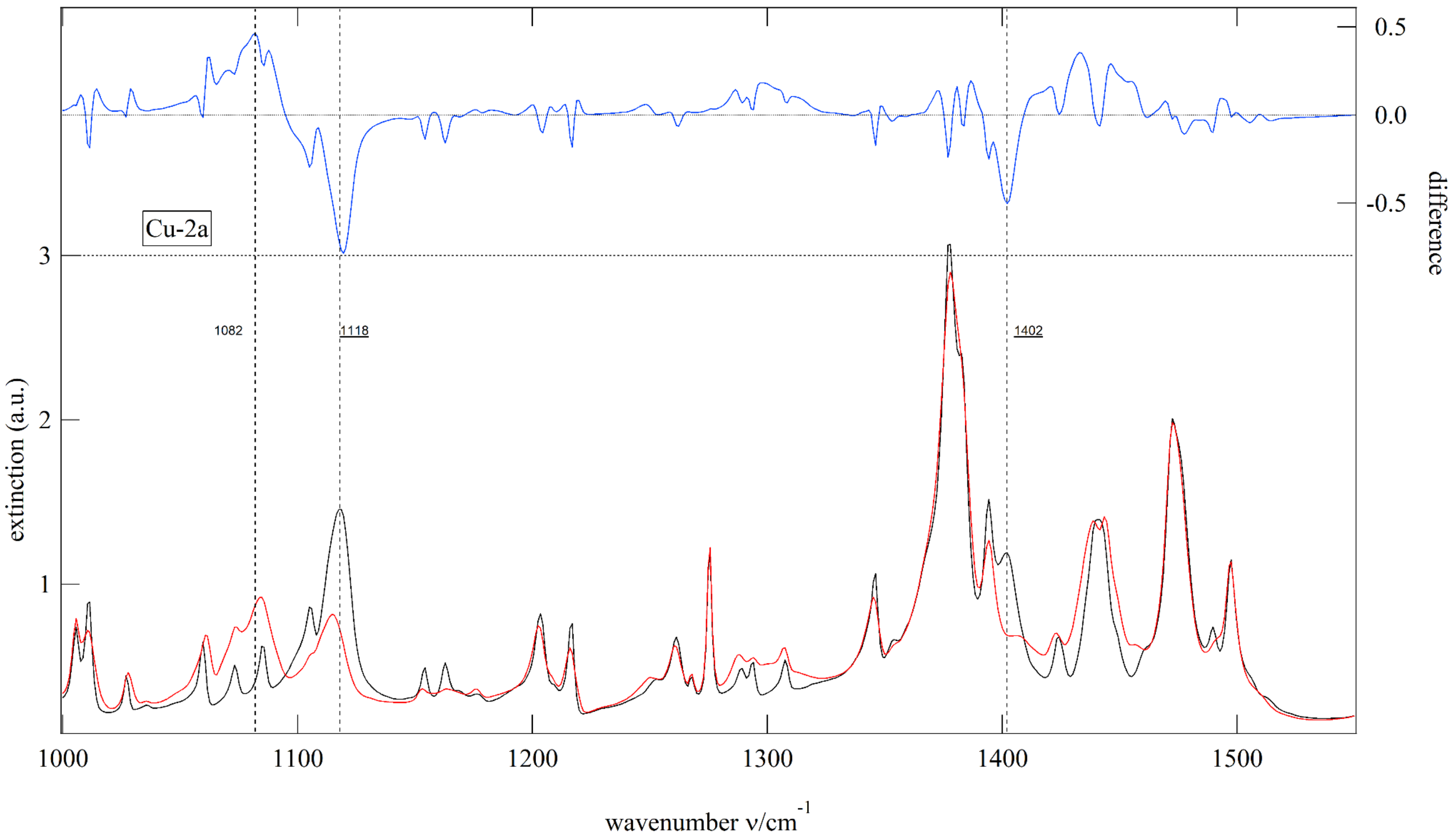

Figure 17S. Expansion of the 1000−1550 cm^{-1} region of the **Cu-2a** complex IR absorption spectra. Black solid line denotes the ground state spectrum, red line presents the spectrum collected after 10 min of LED irradiation, while blue line shows the difference spectrum. Observations with respect to the computational results (Table 8S): decrease of the bands at 1118 and 1402 cm^{-1} corresponding to the ν(N−O) and ν(N=O) mode of the κ-nitrito configuration, respectively, and appearance of a new band at 1082 cm^{-1} which might correspond to the ν(N−O) mode of an *endo*-nitrito configuration, for which also the increase around 1430 cm^{-1} could be a signature (ν(N=O)).

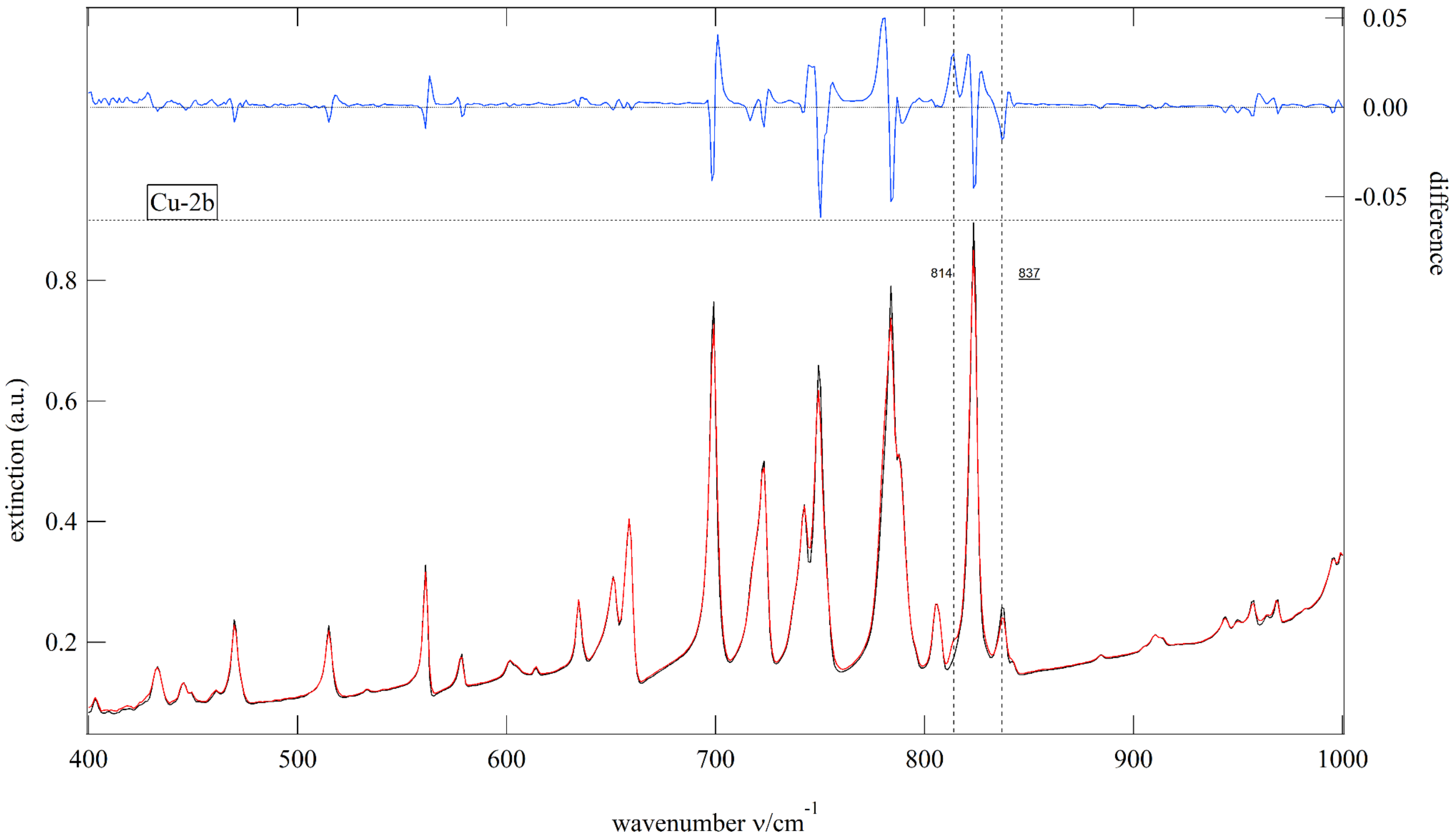

Figure 18S. Expansion of the 400–1000 cm^{-1} region of the **Cu-2b** complex IR absorption spectra. Black solid line denotes the ground state spectrum, red line presents the spectrum collected after 10 min of LED irradiation, while blue line shows the difference spectrum. Observations with respect to the computational results (Table 8S): decrease of the band at 837 cm^{-1} corresponding to the δ(ONO) mode of the κ-nitrito configuration and appearance of a new band at 814 cm^{-1} corresponding to the δ(NO_2) mode of the nitro configuration.

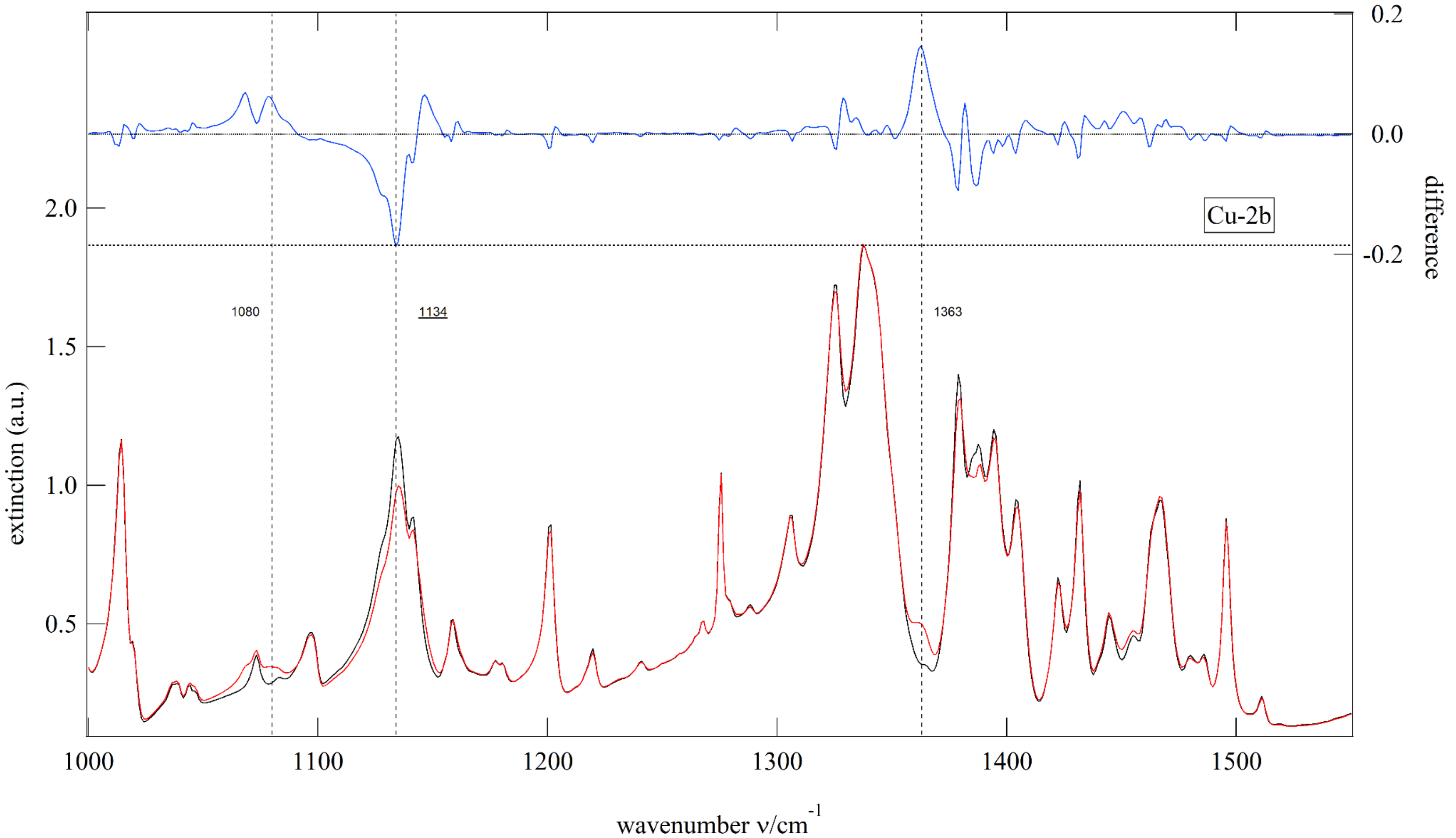

Figure 19S. Expansion of the 1000–1550 cm^{-1} region of the **Cu-2b** complex IR absorption spectra. Black solid line denotes the ground state spectrum, red line presents the spectrum collected after 10 min of LED irradiation, while blue line shows the difference spectrum. Observations with respect to the computational results (Table 8S): decrease of the band at 1134 cm^{-1} corresponding to the ν(N–O) mode of the κ-nitrito configuration and appearance of a new band at 1363 cm^{-1} corresponding to the ν_s(N–O) mode of the nitro configuration. The new band at 1080 cm^{-1} might correspond to the ν(N–O) mode of an *endo*-nitrito configuration.

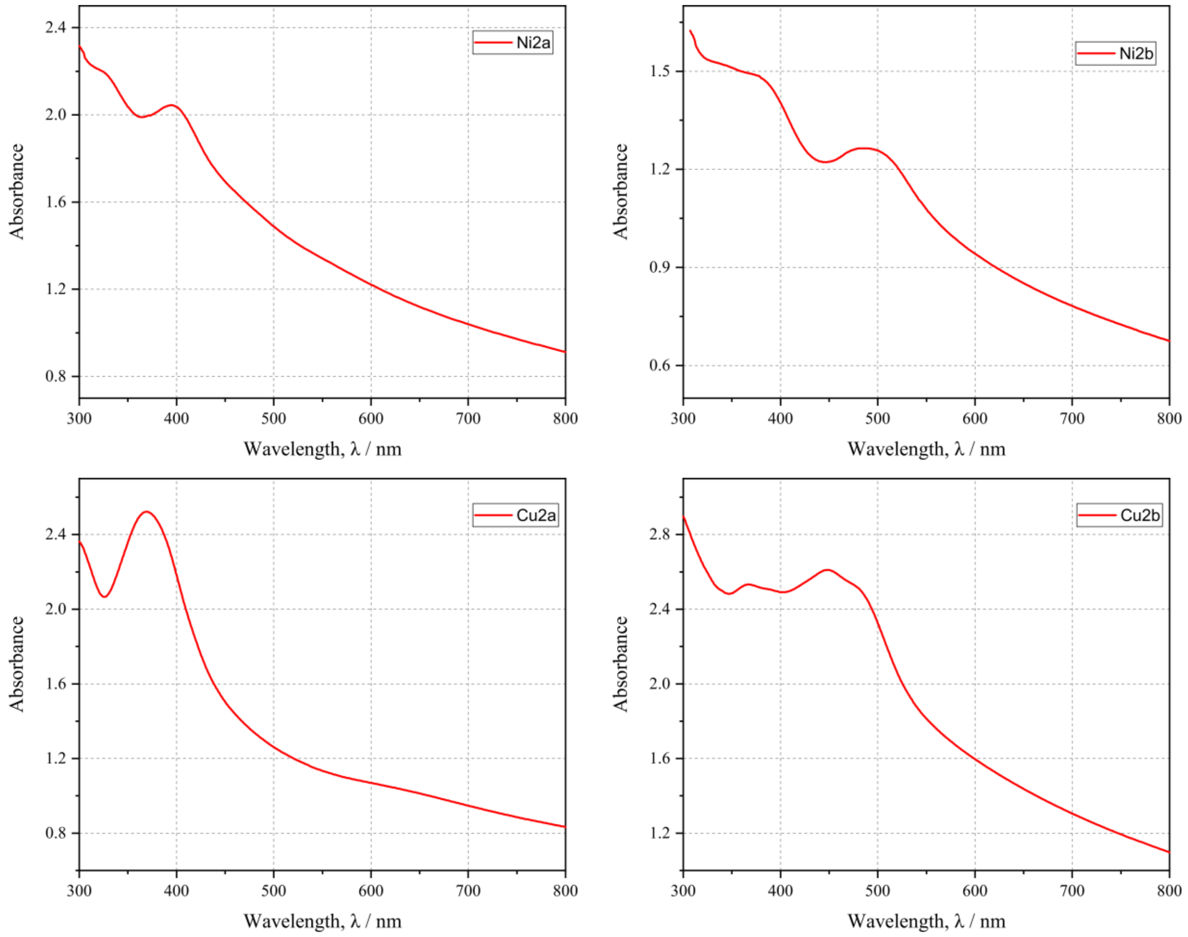

Figure 20S. Solid-state UV-Vis spectra collected for all studied samples at ambient conditions.

Table 10S. Energy differences (ΔE_{rel}) between the ground-state κ-nitrito and metastable nitro linkage isomers computed based on the optimized isolated-molecule geometries. Computations were performed at the DFT(B3LYP)/6-311++G** level of theory.

		ΔE_{rel} / kJ·mol^{-1}
Complex	*Form*	*Isolated mol.*
**Cu-2a**	κ-nitrito	0.0
	nitro	+12.6
**Cu-2b**	κ-nitrito	0.0
	nitro	+7.8
