# Supplementary figures and images for: Exploring Photoswitchable Properties of Two Nitro Nickel(II) Complexes with (N,N,O)-Donor Ligands and Their Copper(II) Analogues
Source: Inorg Chem. 2022 Apr 16;61(17):6624–40. doi: 10.1021/acs.inorgchem.2c00526 (PMC9066408; doi:10.1021/acs.inorgchem.2c00526)

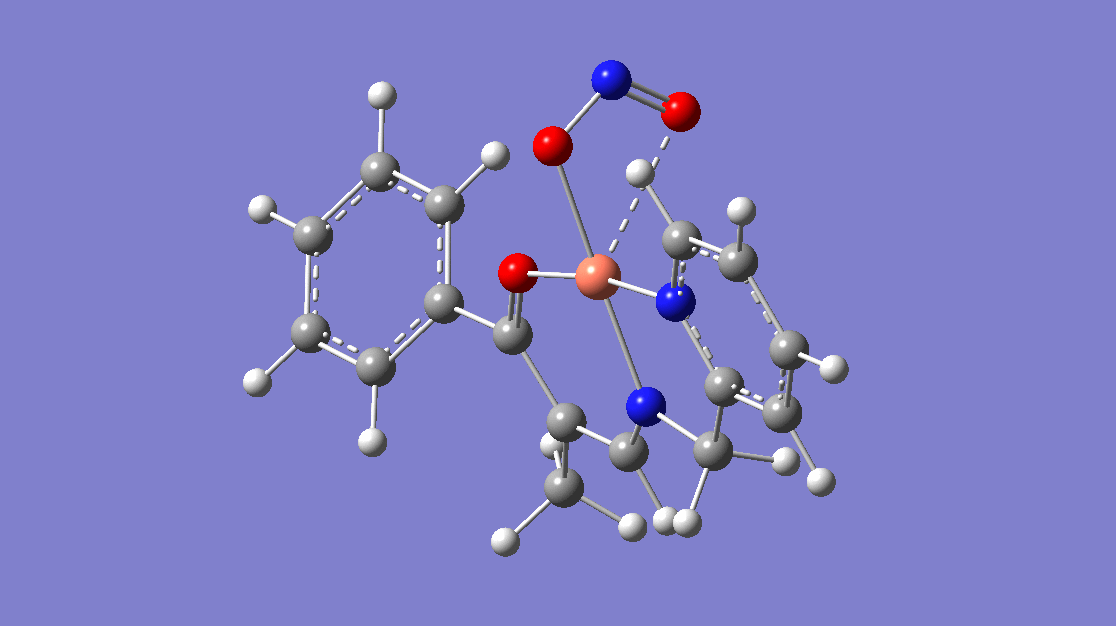

Supplement: Supplementary file 2 — ic2c00526_si_002.zip [file ic2c00526_si_002.zip › gifs/Cu-2a/nitrito/NdoubleO-1454.98.gif]

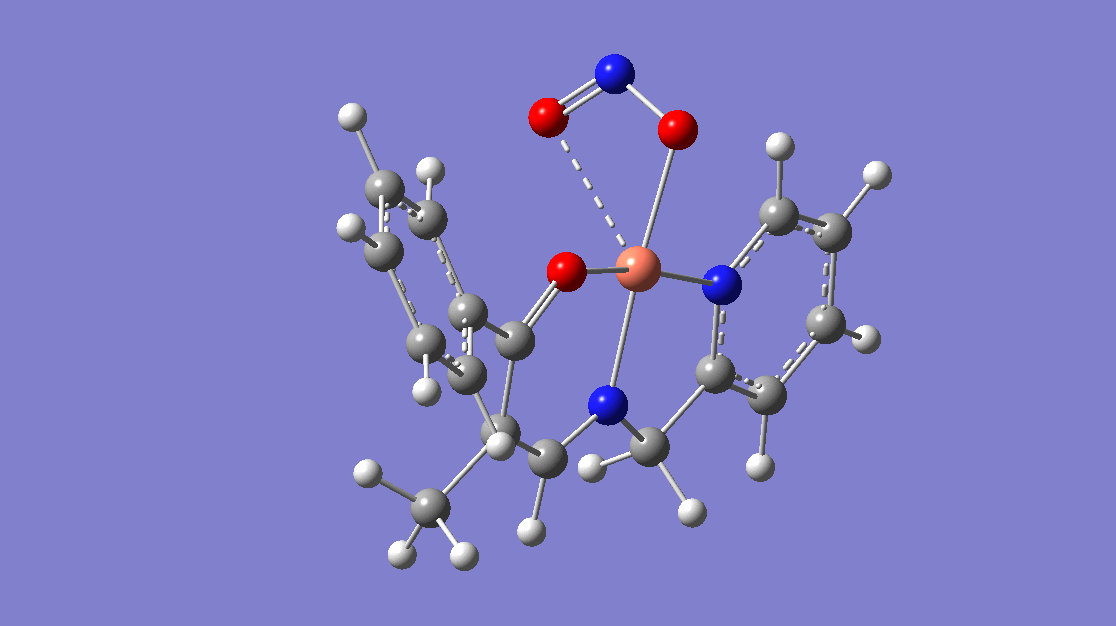

Supplement: Supplementary file 2 — ic2c00526_si_002.zip [file ic2c00526_si_002.zip › gifs/Cu-2a/nitrito/NsingleO-1154.95.gif]

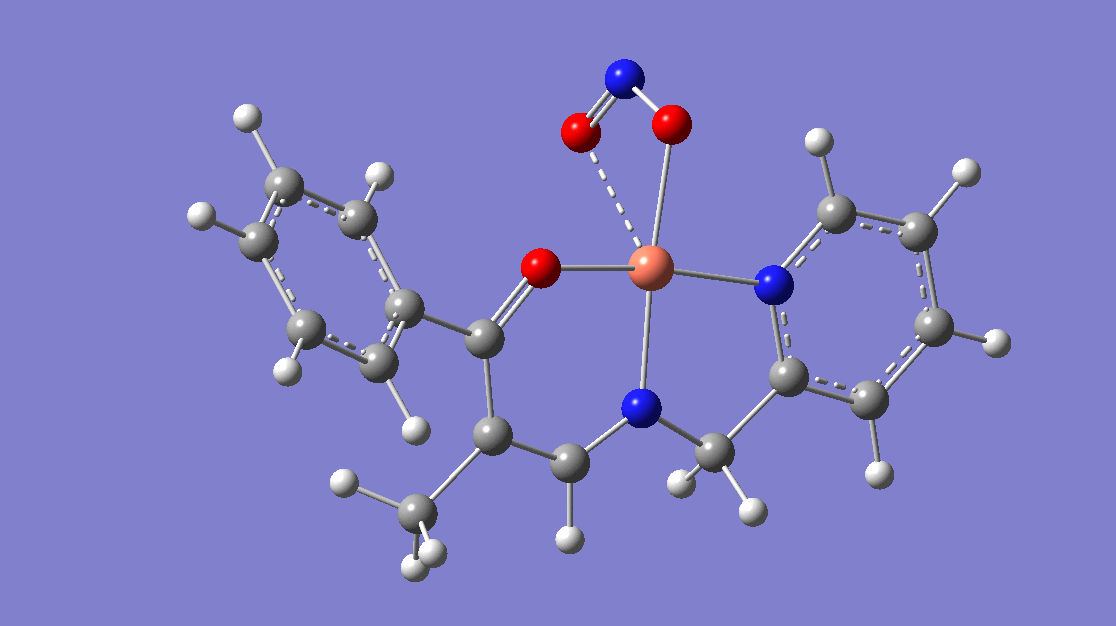

Supplement: Supplementary file 2 — ic2c00526_si_002.zip [file ic2c00526_si_002.zip › gifs/Cu-2a/nitrito/scissoring-855.42.gif]

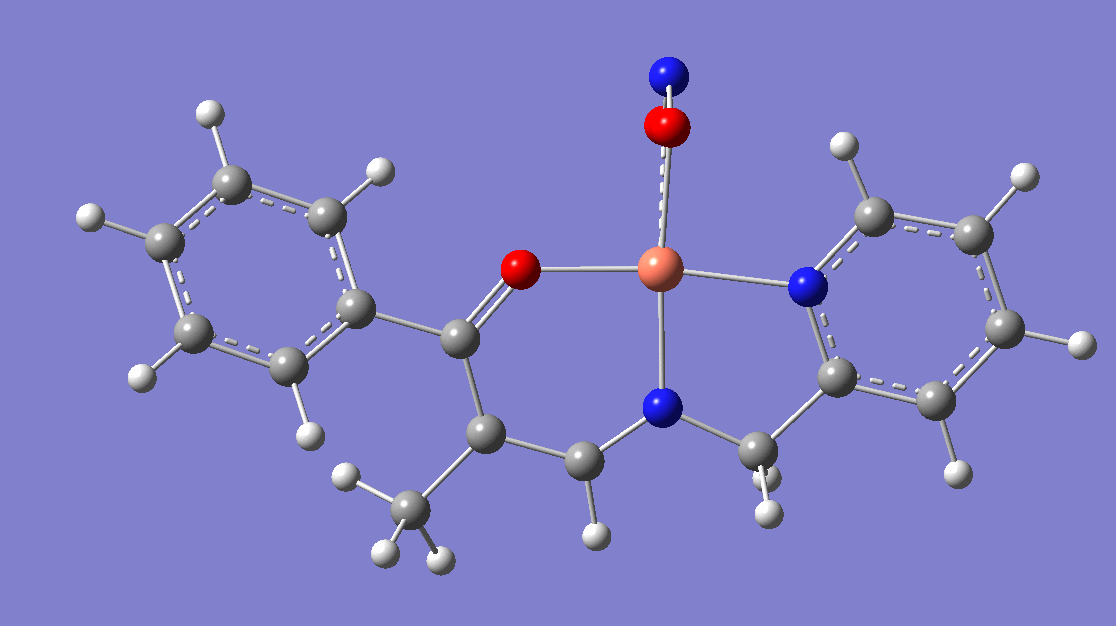

Supplement: Supplementary file 2 — ic2c00526_si_002.zip [file ic2c00526_si_002.zip › gifs/Cu-2a/nitrito/wagging-347.65.gif]

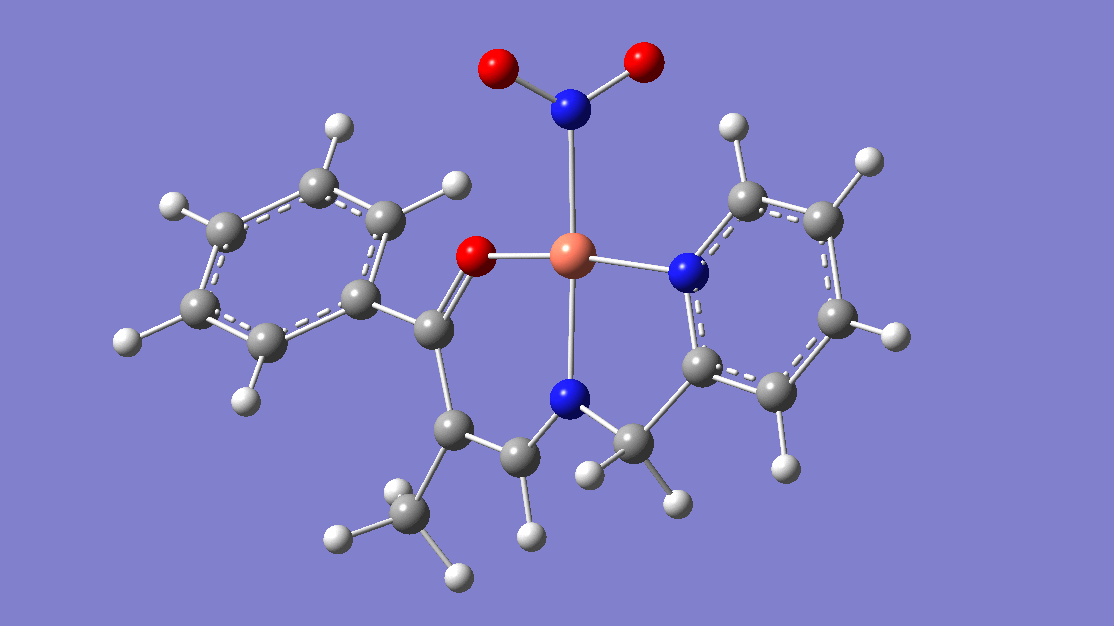

Supplement: Supplementary file 2 — ic2c00526_si_002.zip [file ic2c00526_si_002.zip › gifs/Cu-2a/nitro/asym-1488.41.gif]

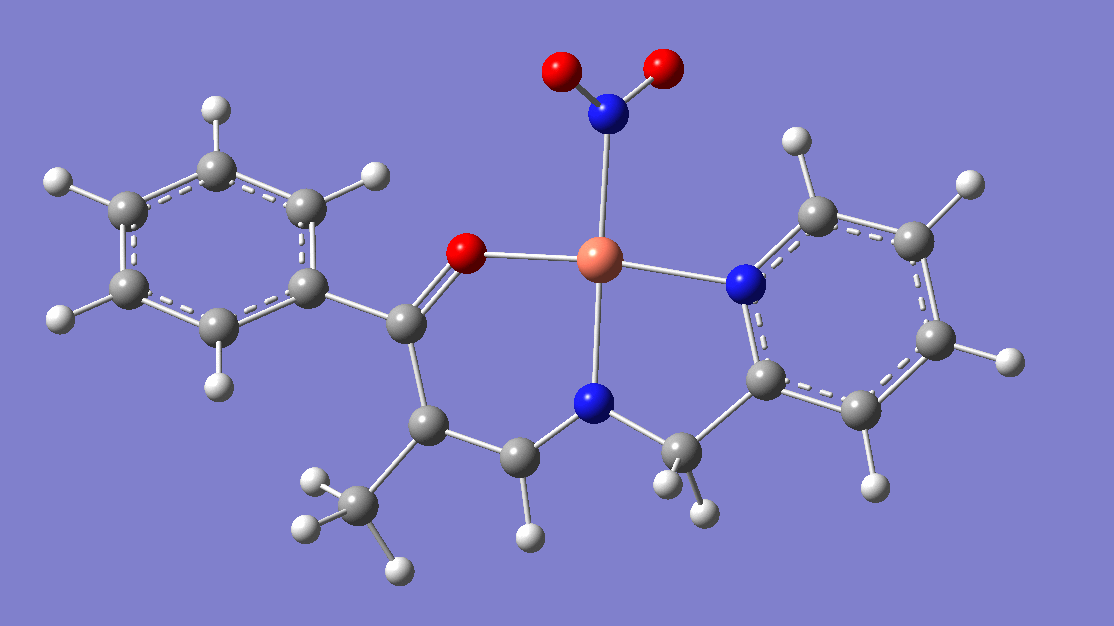

Supplement: Supplementary file 2 — ic2c00526_si_002.zip [file ic2c00526_si_002.zip › gifs/Cu-2a/nitro/scissoring-820.15.gif]

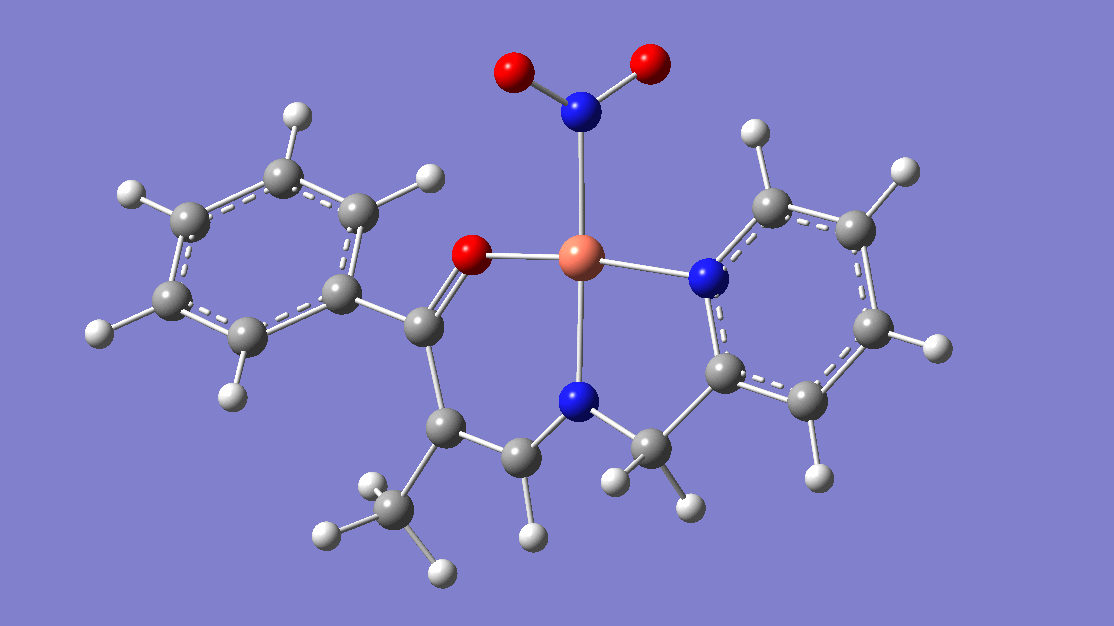

Supplement: Supplementary file 2 — ic2c00526_si_002.zip [file ic2c00526_si_002.zip › gifs/Cu-2a/nitro/sym-1366.82.gif]

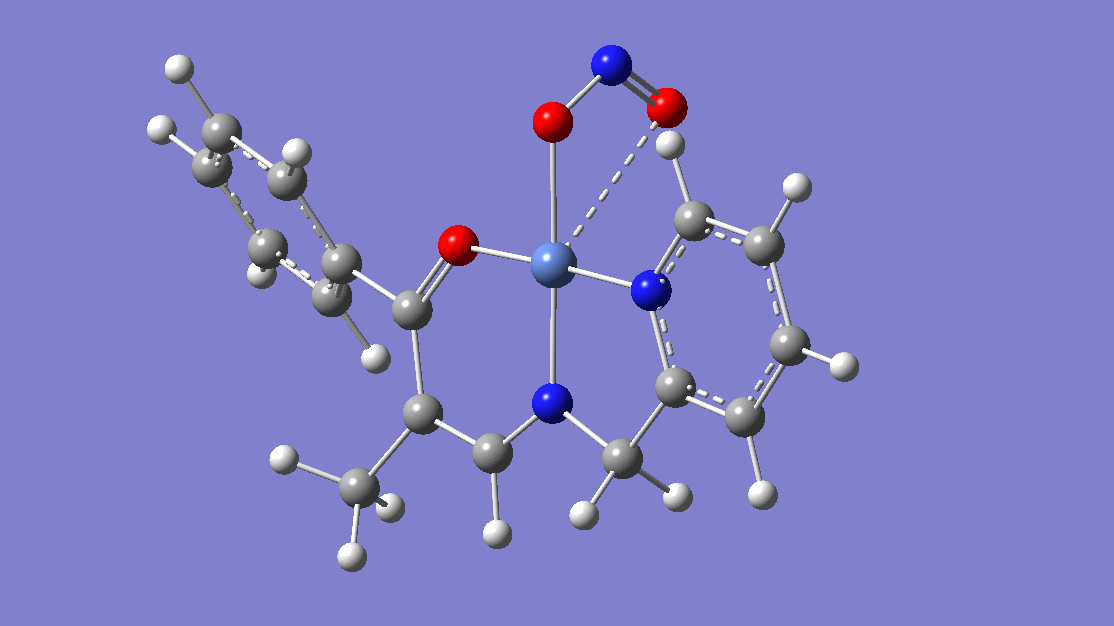

Supplement: Supplementary file 2 — ic2c00526_si_002.zip [file ic2c00526_si_002.zip › gifs/Ni-2a/nitrito/NdoubleO-1507.30.gif]

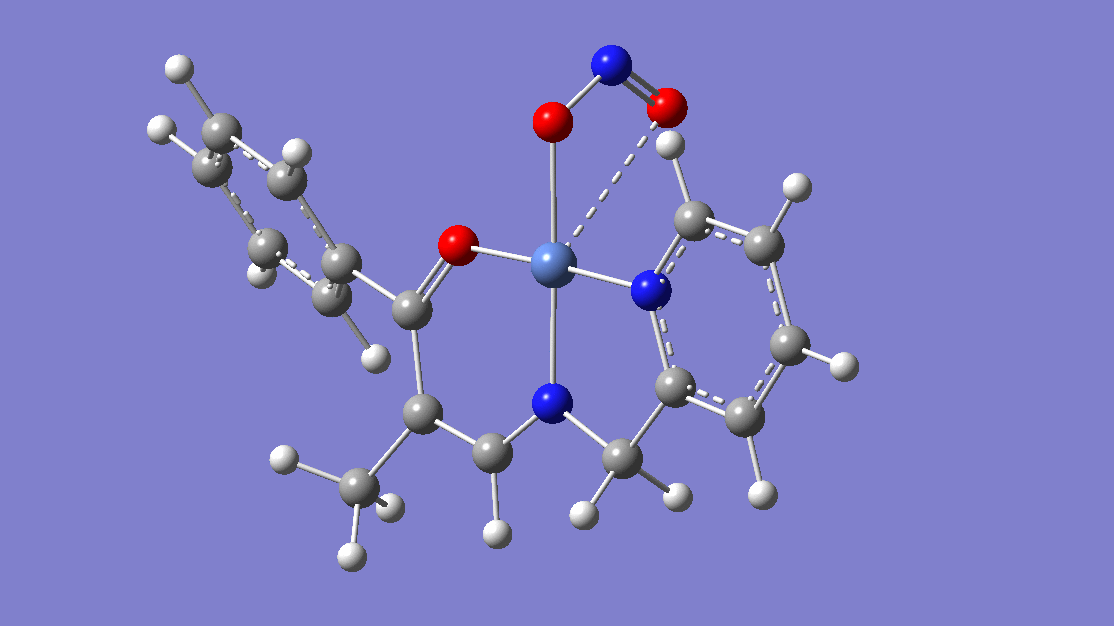

Supplement: Supplementary file 2 — ic2c00526_si_002.zip [file ic2c00526_si_002.zip › gifs/Ni-2a/nitrito/NsingleO-1094.74.gif]

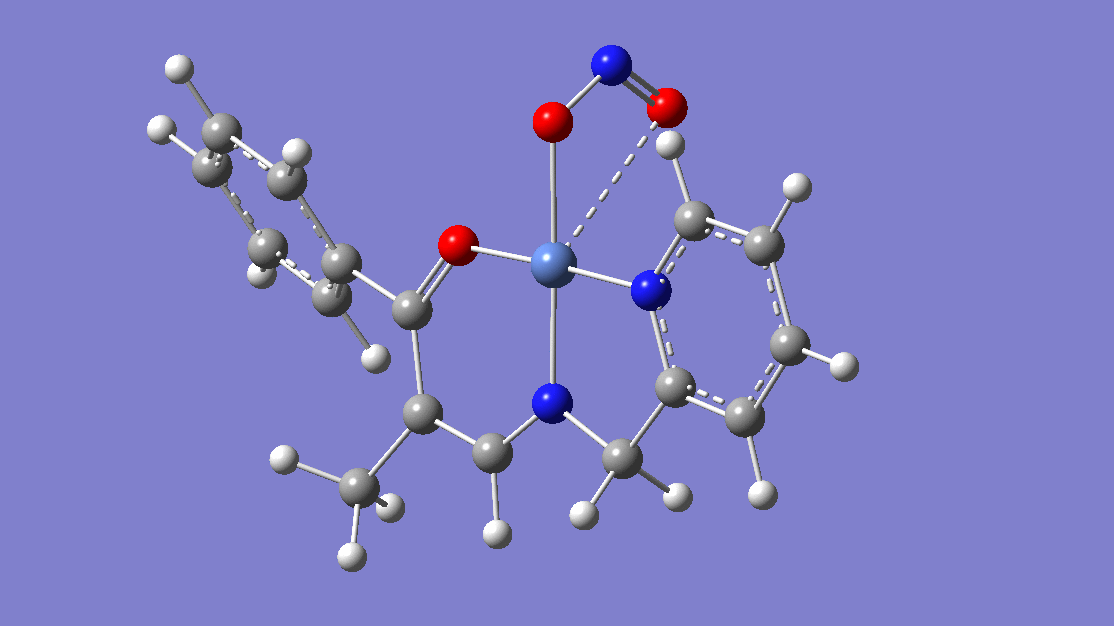

Supplement: Supplementary file 2 — ic2c00526_si_002.zip [file ic2c00526_si_002.zip › gifs/Ni-2a/nitrito/scissoring-845.05.gif]

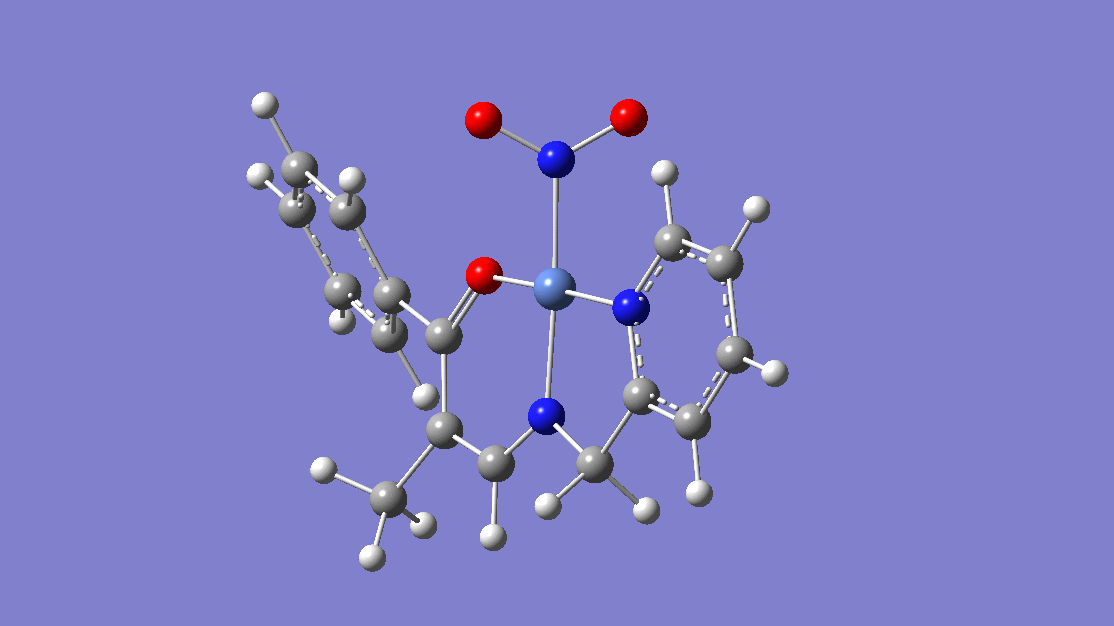

Supplement: Supplementary file 2 — ic2c00526_si_002.zip [file ic2c00526_si_002.zip › gifs/Ni-2a/nitro/asym-1493.96.gif]

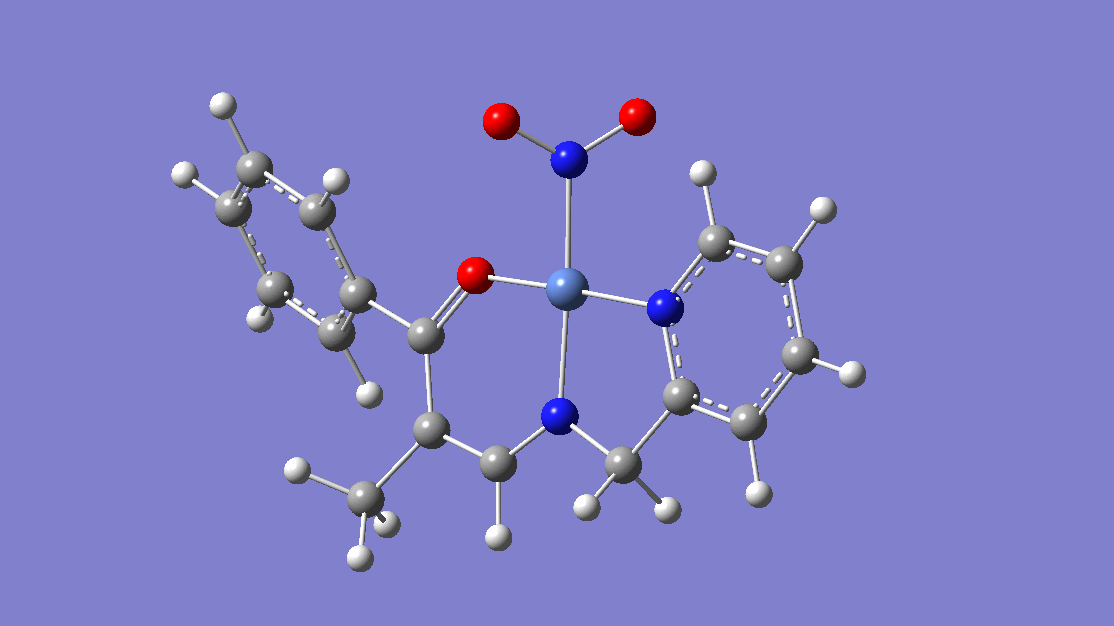

Supplement: Supplementary file 2 — ic2c00526_si_002.zip [file ic2c00526_si_002.zip › gifs/Ni-2a/nitro/scissoring-835.94.gif]

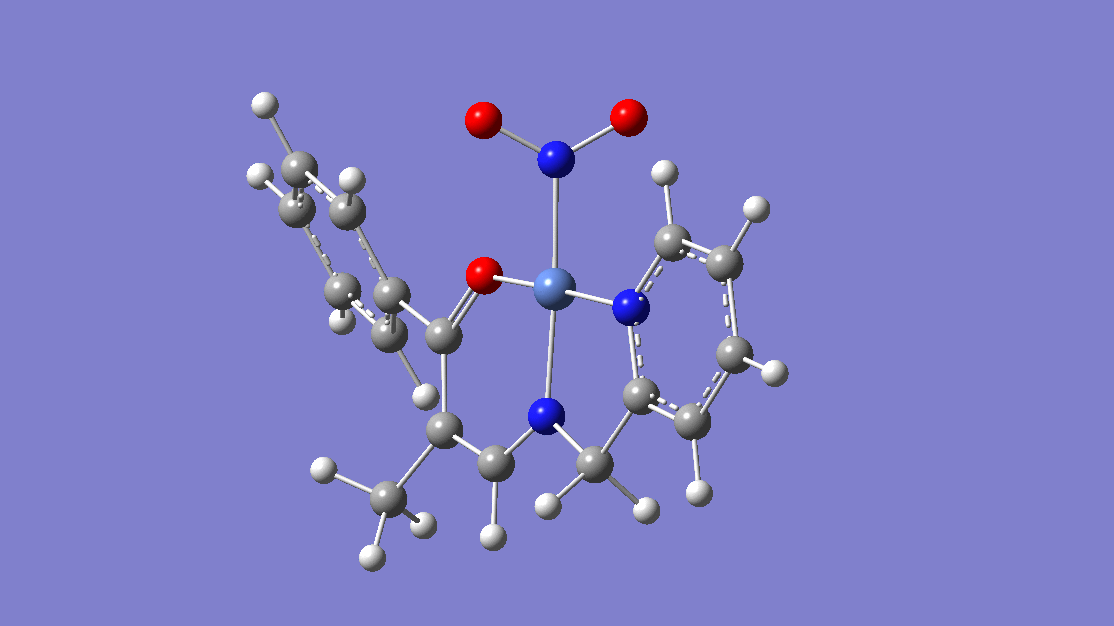

Supplement: Supplementary file 2 — ic2c00526_si_002.zip [file ic2c00526_si_002.zip › gifs/Ni-2a/nitro/sym-1391.62.gif]

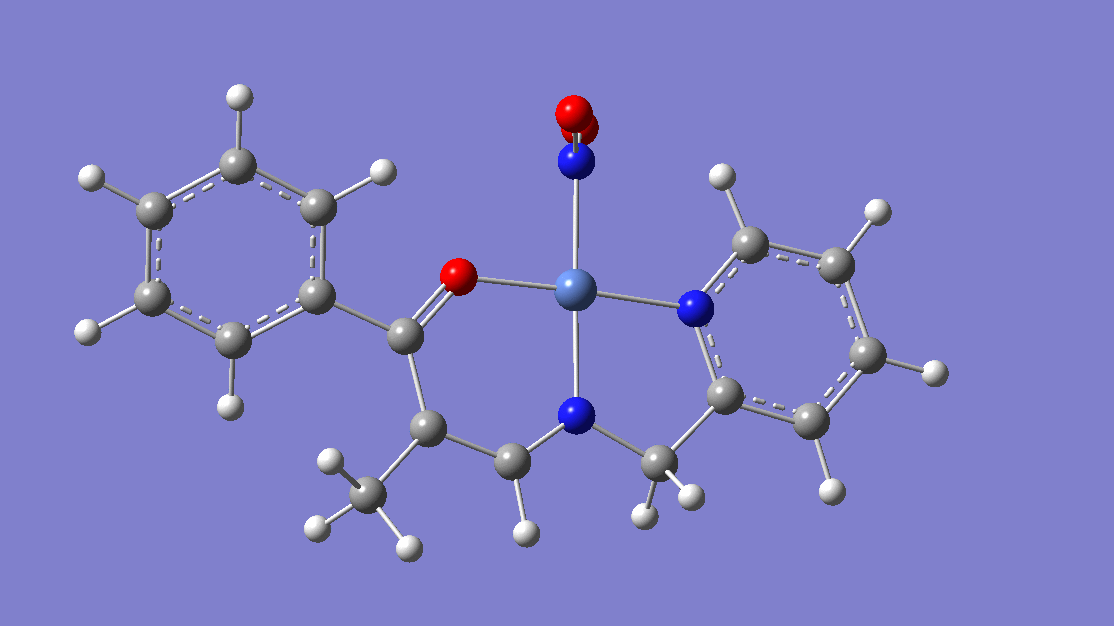

Supplement: Supplementary file 2 — ic2c00526_si_002.zip [file ic2c00526_si_002.zip › gifs/Ni-2a/nitro/wagging-581.02.gif]
